# Supplementary material for: Know-do gap for sick child care and drivers of knowledge and practice among health extension workers in four regions of Ethiopia: a cross-sectional study
Source: BMJ Open. 2023 Aug 23;13(8):e069698. doi: 10.1136/bmjopen-2022-069698 (PMC10450039; doi:10.1136/bmjopen-2022-069698)
Supplement: Supplementary data [file bmjopen-2022-069698supp001.pdf]

S1 Table. Medical equipment, job aids, and medicine availability at the health posts of providers in four regions of Ethiopia, December 2018 to February 2019 (N=147)

Medical equipment, job aids, and Medicines	n(%)
Medical equipment	
Thermometer	121(82.3)
Infant scale	115(78.2)
Weighing sling	116(78.9)
Tape measures	79(53.7)
Blood pressure apparatus	69(46.9)
Stethoscopes	98(66.7)
Watch or clock (timer)	16(10.9)
Examination couch	102(69.4)
Mid-upper-arm circumference tape	147(100.0)
Basic equipment availability index[a]	44(29.9)
Job aids	
iCCM register (2-59 months old children)	145(98.6)
Stock/bin cards to track medicines	97(66.0)
Medicines request and resupply forms	95(64.6)
Chart booklets	138(93.9)
Medicines	
Amoxicillin	115(78.2)
Oral rehydration solution	137(93.2)
Zinc	125(85.0)
Oral rehydration solution and zinc	117(79.6)
Paracetamol	94(63.9)
Amoxicillin, oral rehydration solution and zinc	96(65.3)

[a] *Basic equipment availability index: Availability of all of the following for the same provider: thermometer, infant scale, weighing sling, stethoscope, and BP apparatus*
