# Supplementary material for: Know-do gap for sick child care and drivers of knowledge and practice among health extension workers in four regions of Ethiopia: a cross-sectional study
Source: BMJ Open. 2023 Aug 23;13(8):e069698. doi: 10.1136/bmjopen-2022-069698 (PMC10450039; doi:10.1136/bmjopen-2022-069698)
Supplement: Supplementary data [file bmjopen-2022-069698supp002.pdf]

S2 Table. Providers knowledge of childhood pneumonia and diarrhea management in four regions of Ethiopia, December 2018 to February 2019 (N=274)

Knowledge of-	n	%(95% CI)
Pneumonia signs and symptoms		
Good knowledge	97	35.4(29.9-41.2)
Poor knowledge	177	64.6(58.8-70.1)
Pneumonia management approaches		
Good knowledge	67	24.5(19.6-29.8)
Poor knowledge	207	75.5(70.2-80.4)
Pneumonia signs and symptoms and management approaches		
Good knowledge	108	39.4(33.7-45.3)
Poor knowledge	166	60.6(54.7-66.3)
Diarrhea signs and symptoms		
Good knowledge	57	20.8(16.3-25.9)
Poor knowledge	217	79.2(74.1-83.7)
Diarrhea management approaches		
Good knowledge	66	24.1(19.3-29.4)
Poor knowledge	208	75.9(70.6-80.7)
Diarrhea signs and symptoms and management approaches		
Good knowledge	78	28.5(23.4-34.0)
Poor knowledge	196	71.5(66.0-76.6)
Both illnesses sign and symptoms and management approaches		
Good knowledge	74	27.0(22.0-32.5)
Poor knowledge	200	73.0(67.5-78.0)
