# Supplementary material for: Know-do gap for sick child care and drivers of knowledge and practice among health extension workers in four regions of Ethiopia: a cross-sectional study
Source: BMJ Open. 2023 Aug 23;13(8):e069698. doi: 10.1136/bmjopen-2022-069698 (PMC10450039; doi:10.1136/bmjopen-2022-069698)
Supplement: Author's manuscript [file bmjopen-2022-069698.draft_revisions.pdf]

BMJ Open

BMJ Open is committed to open peer review. As part of this commitment we make the peer review history of every article we publish publicly available.

When an article is published we post the peer reviewers' comments and the authors' responses online. We also post the versions of the paper that were used during peer review. These are the versions that the peer review comments apply to.

The versions of the paper that follow are the versions that were submitted during the peer review process. They are not the versions of record or the final published versions. They should not be cited or distributed as the published version of this manuscript.

BMJ Open is an open access journal and the full, final, typeset and author-corrected version of record of the manuscript is available on our site with no access controls, subscription charges or pay-per-view fees (http://bmjopen.bmj.com).

If you have any questions on BMJ Open's open peer review process please email info.bmjopen@bmj.com

BMJ Open

Know-do gap for sick child care among health extension workers in Ethiopia

Journal:	*BMJ Open*
Manuscript ID	bmjopen-2022-069698
Article Type:	Original research
Date Submitted by the Author:	29-Oct-2022
Complete List of Authors:	Daka, Dawit; Jimma University<br>Wordofa, Muluemebet; Jimma University, Department of Population & Family Health<br>Woldie, Mirkuzie; Jimma University; Jimma University, Health Policy and Management
Keywords:	Community child health < PAEDIATRICS, Quality in health care < HEALTH SERVICES ADMINISTRATION & MANAGEMENT, INFECTIOUS DISEASES, Public health < INFECTIOUS DISEASES

SCHOLARONE™
Manuscripts

I, the Submitting Author has the right to grant and does grant on behalf of all authors of the Work (as defined in the below author licence), an exclusive licence and/or a non-exclusive licence for contributions from authors who are: i) UK Crown employees; ii) where BMJ has agreed a CC-BY licence shall apply, and/or iii) in accordance with the terms applicable for US Federal Government officers or employees acting as part of their official duties; on a worldwide, perpetual, irrevocable, royalty-free basis to BMJ Publishing Group Ltd ("BMJ") its licensees and where the relevant Journal is co-owned by BMJ to the co-owners of the Journal, to publish the Work in this journal and any other BMJ products and to exploit all rights, as set out in our licence.

The Submitting Author accepts and understands that any supply made under these terms is made by BMJ to the Submitting Author unless you are acting as an employee on behalf of your employer or a postgraduate student of an affiliated institution which is paying any applicable article publishing charge ("APC") for Open Access articles. Where the Submitting Author wishes to make the Work available on an Open Access basis (and intends to pay the relevant APC), the terms of reuse of such Open Access shall be governed by a Creative Commons licence – details of these licences and which Creative Commons licence will apply to this Work are set out in our licence referred to above.

Other than as permitted in any relevant BMJ Author's Self Archiving Policies, I confirm this Work has not been accepted for publication elsewhere, is not being considered for publication elsewhere and does not duplicate material already published. I confirm all authors consent to publication of this Work and authorise the granting of this licence.

For peer review only

STROBE Statement—Checklist of items that should be included in reports of ***cross-sectional studies***

	Item No	Recommendation	Page No
**Title and abstract**	1	(*a*) Indicate the study's design with a commonly used term in the title or the abstract	2
		(*b*) Provide in the abstract an informative and balanced summary of what was done and what was found	2&3
**Introduction**			
Background/rationale	2	Explain the scientific background and rationale for the investigation being reported	3-5
Objectives	3	State specific objectives, including any prespecified hypotheses	5
**Methods**			
Study design	4	Present key elements of study design early in the paper	5
Setting	5	Describe the setting, locations, and relevant dates, including periods of recruitment, exposure, follow-up, and data collection	5-6
Participants	6	(*a*) Give the eligibility criteria, and the sources and methods of selection of participants	6
Variables	7	Clearly define all outcomes, exposures, predictors, potential confounders, and effect modifiers. Give diagnostic criteria, if applicable	7-8
Data sources/ measurement	8*	For each variable of interest, give sources of data and details of methods of assessment (measurement). Describe comparability of assessment methods if there is more than one group	6-7
Bias	9	Describe any efforts to address potential sources of bias	7
Study size	10	Explain how the study size was arrived at	6
Quantitative variables	11	Explain how quantitative variables were handled in the analyses. If applicable, describe which groupings were chosen and why	9
Statistical methods	12	(*a*) Describe all statistical methods, including those used to control for confounding	9
		(*b*) Describe any methods used to examine subgroups and interactions	
		(*c*) Explain how missing data were addressed	
		(*d*) If applicable, describe analytical methods taking account of sampling strategy	
		(*e*) Describe any sensitivity analyses	
**Results**			
Participants	13*	(a) Report numbers of individuals at each stage of study—eg numbers potentially eligible, examined for eligibility, confirmed eligible, included in the study, completing follow-up, and analysed	10
		(b) Give reasons for non-participation at each stage	
		(c) Consider use of a flow diagram	
Descriptive data	14*	(a) Give characteristics of study participants (eg demographic, clinical, social) and information on exposures and potential confounders	10-11
		(b) Indicate number of participants with missing data for each variable of interest	
Outcome data	15*	Report numbers of outcome events or summary measures	13-16
Main results	16	(*a*) Give unadjusted estimates and, if applicable, confounder-adjusted estimates and their precision (eg, 95% confidence interval). Make clear which confounders were adjusted for and why they were included	16-18

For peer review only

		(*b*) Report category boundaries when continuous variables were categorized	
		(*c*) If relevant, consider translating estimates of relative risk into absolute risk for a meaningful time period	
Other analyses	17	Report other analyses done—eg analyses of subgroups and interactions, and sensitivity analyses	
**Discussion**			
Key results	18	Summarise key results with reference to study objectives	18
Limitations	19	Discuss limitations of the study, taking into account sources of potential bias or imprecision. Discuss both direction and magnitude of any potential bias	20-21
Interpretation	20	Give a cautious overall interpretation of results considering objectives, limitations, multiplicity of analyses, results from similar studies, and other relevant evidence	18-21
Generalisability	21	Discuss the generalisability (external validity) of the study results	18-21
**Other information**			
Funding	22	Give the source of funding and the role of the funders for the present study and, if applicable, for the original study on which the present article is based	22

*Give information separately for exposed and unexposed groups.

Note: An Explanation and Elaboration article discusses each checklist item and gives methodological background and published examples of transparent reporting. The STROBE checklist is best used in conjunction with this article (freely available on the Web sites of PLoS Medicine at http://www.plosmedicine.org/, Annals of Internal Medicine at http://www.annals.org/, and Epidemiology at http://www.epidem.com/). Information on the STROBE Initiative is available at www.strobe-statement.org.

For peer review only

For peer review only - http://bmjopen.bmj.com/site/about/guidelines.xhtml

Know-do gap for sick child care among health extension workers in Ethiopia

Dawit Wolde Daka[1*], Muluemebet Abera Wordofa[2], and Mirkuzie Woldie[1,3]

[1]Faculty of Public Health, Department of Health Policy and Management, Jimma University, Jimma, Ethiopia

[2]Faculty of Public Health, Department of Population and Family Health, Jimma University, Jimma, Ethiopia

[3] Fenot Project, University of British Columbia, School of Public Health and Population, Addis Ababa, Ethiopia

*Corresponding Author

E-mail: dave86520@gmail.com

https://orcid.org/0000-0001-5465-6345

For peer review only

Abstract

Objective: Though efforts were made to expand community-based sick child healthcare in Ethiopia, the quality of care provided to sick under-five children remained low. This study examined the gap between what health extension workers knew and did during the management of sick children visiting the health posts in Ethiopia.

Design: Cross-sectional study was conducted from December 2018 to February 2019 among 150 health extension workers in four regional states in Ethiopia. We have used data from health workers' interviews, observations of actual practices, and facility assessments.

Outcome: A know-do gap is difference between knowledge scores and actual practice scores of the HEWs, and logistic regression analysis was used to identify predictors of knowledge and actual practice.

Results: The providers' knowledge and actual practices on pneumonia and diarrhea assessment were 38%, and 21.3% respectively. The providers' correct knowledge ranged from 27.8% to 76.0% for signs and symptoms of pneumonia, and 32.0% to 84% for diarrhea signs. Their actual practices ranged from 15.1% to 47.3% for pneumonia, and 27.0% to 42.6% for diarrhea. Similarly, the correct knowledge and actual practices for pneumonia and dehydration management were 88.3% vs 15.6% and 93.9% vs 51.3%, respectively. There was significant know-do gap in assessments (16.7%, p=0.002) and management of both conditions (68.5%, p<0.0001). Clinical mentorships were positively associated with providers' knowledge of clinical management, while the availability of medicines to treat both conditions was associated with their management practice.

For peer review only

Conclusions: While knowledge and actual practice for management of pneumonia and diarrhea ranged from very low to high, what is more concerning is the huge know-do gap among the HEWs. Our findings suggest that knowledge-based training is necessary but not sufficient for ensuring the correct assessment and management of sick children by the HEWs. Continuous support through mentorships and the supply of commodities are critically needed.

Keywords: Knowledge, actual practice, Know-do gap, pneumonia, diarrhea, assessment, management, health extension workers, Ethiopia

Strength and limitations

- This is the first study that examined the know-do gap of sick childcare among health extension workers in Ethiopia
- Observations of sick child consultations by health extension workers were performed by clinicians who had received refresher training on integrated community case management (iCCM)
- Though the findings are limited by sample size, the study has covered the four largest regions of the country and is representative of the different settings in rural Ethiopia.

Introduction

Pneumonia and diarrhea remain the two major childhood illnesses that contributed to the larger portion of under-five mortality worldwide (1,2). Globally, over 1.23 million children died of pneumonia and diarrhea before reaching their fifth birthday in 2018. Seventy percent of all pneumonia and diarrhea deaths in children under the age of five years have occurred in 15 countries of the world alone, and Ethiopia is one among them (3,4).

For peer review only - http://bmjopen.bmj.com/site/about/guidelines.xhtml

The integrated community case management (iCCM) approach is a key strategy for improving the treatment of potentially life-threatening illnesses in children (5–7). The iCCM guideline recommends that all children 2-59 months of age are first assessed for common childhood illnesses and classified based on assessment results and treated according to the standard of care(8). Adherence to guidelines by front-line providers is critical to improving the quality of care and improving treatment outcomes (9). However, providers' adherence to clinical standards of sick children management is low in most low- and middle-income settings (10–12) and significant know-do gap of clinical practices was common among the health care providers(13,14).

The know-do gap, failure to apply existing knowledge to improve health, has huge impact on the effort made towards reducing under-five mortalities. Though much is known about the evidence-based interventions related to child healthcare, it is not practiced as intended and the practices vary across the different settings (15). Most of the know-do gap is a result of inadequate skilled human, material, and financial resources(16).

Often providers express that they know about the assessment and management of sick children, but only few of them perform those tasks according to the standard. A study conducted in Ethiopia among health facility-level providers revealed that there was a gap between the knowledge of providers and their actual performance in child healthcare (14). Similarly, a study done in rural India revealed a large know-do gap in pneumonia and diarrhea management by healthcare providers (17) and a know-do gap in neonatal care among informal healthcare providers(18). The knowledge and actual practices of providers are related to factors including background characteristics of providers, presence of medical commodities, community support, and the presence of trainings, mentoring, and supportive supervision(11,13,19–21). Adequately supported

For peer review only - http://bmjopen.bmj.com/site/about/guidelines.xhtml

CHWs are able to manage sick children according to the protocol in the majority of the cases (22–24).

Narrowing the know-do gap is critical to attaining child survival goals and the targets set under the Global Action Plan for the Prevention and Control of Pneumonia and Diarrhea (GAPPD) in 2025. One of the core interventions outlined in the action plan was the appropriate management of sick children who are at risk of serious illnesses or death due to these two diseases (pneumonia and diarrhea) (4).

In Ethiopia, iCCM implementation was started in 2010 as part of the health extension program(22,25). Currently, iCCM is implemented in more than 18,000 health posts nationally (26). Despite huge investments and high coverage of clinical training (22,27), the quality of sick childcare is low in the health posts of Ethiopia (28–30). On the other hand, little is known about the know-do gap of sick child assessment and management, and the factors that cause it among HEWs in Ethiopia. Therefore, this paper aimed to examine the level of agreement between the knowledge the health extension workers had about assessment and management and their actual practice of childhood pneumonia and diarrhea.

Methods

Study design and setting

Facility-based cross-sectional study was conducted among HEWs from December 2018 to February 2019. The study was conducted in the health posts of 52 districts across four populous regions of Ethiopia: Oromia, Amhara, SNNP, and Tigray. This paper was part of the larger Dagu end-line survey, a survey that aimed to estimate the effectiveness of the optimizing health

For peer review only

For peer review only - http://bmjopen.bmj.com/site/about/guidelines.xhtml

extension program (OHEP) in Ethiopia. The protocol for OHEP evaluation was published elsewhere (31).

Study participants and selection procedure

Two-stage stratified sampling strategy was used to select the study participants from the districts. In the first stage enumeration areas (EAs) were selected from the study districts using the list of EAs from the 2007 Ethiopian Housing and Population Census as the sampling frame. The cumulative population size to clusters across the study areas was calculated and 200 EAs were selected with a probability proportional to population size (PPS). Each EA formed a cluster and each cluster constituted a primary sampling unit. In the second stage, all health posts serving the catchment population of the selected EAs were included in the study. Similarly, all HEWs who were present during the survey period were included in the study.

Measurement

Structured interviews, observation, and health post-assessment questionnaires were used to collect data. The questionnaires were developed based on the World Health Organization (WHO) tool for IMCI sick child quality assessment in primary healthcare facilities(32) and the national iCCM guidelines(33). The health provider's interview questions included items such as background characteristics, iCCM training, supportive supervision, clinical mentorships, and knowledge of assessment and management of sick children 2-59 months. The observation questionnaire captured data on the reason for visits, assessment, classification, treatment, referral, and counseling. The facility assessment tool had sections of health posts infrastructure, medical equipment, medicines, and job aids. All the questionnaires were uploaded on tablets (CSPro 7.1) for data collection.

For peer review only

For peer review only - http://bmjopen.bmj.com/site/about/guidelines.xhtml

Data collectors with a qualification of bachelor's degree in health science were recruited and involved in data collection. Likewise, supervisors with a health background and qualifications with a bachelor of science and above have participated. Data collectors and supervisors were trained for 10 days on the questionnaires, and data collection process. The supervisors have monitored the daily data collection process at the field level and provided support.

Study variables

The outcome variable was know-do gap of child care related to pneumonia and diarrhea management. Know-do gap was the difference between knowledge of HEWs about the signs and symptoms and management of pneumonia and diarrhea and their ability to correctly assess those signs and symptoms when children are presented with the illnesses. A provider was regarded as having a know-do gap in the following conditions: (a) if correctly named the signs and symptoms of the illnesses but failed to actually identify them; (b) if unable to correctly named the signs and symptoms of the illnesses but able to identify them, and (c) if unable to correctly named the signs and symptoms of the illnesses as well as unable to correctly identify them. If the provider correctly named signs and symptoms, and also correctly identified them, then he/she was regarded as with no know-do gap. Similarly, the above conditions were applied to the know-do gap of pneumonia and diarrhea management.

A total of 11 knowledge items were used, each having 'yes=1' and 'no=0' responses. Providers were asked to respond to questions without prompts and the responses were recorded from pre-defined list. Then the interviewer recorded the responses of the providers. The knowledge questions were: what are the main signs and symptoms of pneumonia in children 2-59 months (3 responses)? When a child presents with signs and symptoms of pneumonia, what initial measures do you take (2 responses)? What are the main signs and symptoms of diarrhea in children 2-59

For peer review only - http://bmjopen.bmj.com/site/about/guidelines.xhtml

months (4 responses)? When a child presents with signs and symptoms of diarrhea, what initial measures do you take (2 responses)?

Knowledge index was constructed based on the Ethiopian university students' grading system with a few modifications. The Ethiopian university grading system is excellent [85-100%], very good [75 to 85%), good [60-75%), satisfactory [50-60%), unsatisfactory [45,50%), and very poor (below 45%). The providers who scored ≥60% were categorized as having 'Good knowledge/knowledgeable=1', and otherwise 'poor knowledge/not knowledgeable=0'.

Moreover, providers who correctly assessed children presented with respiratory and diarrhea complaints were categorized as 'correct practice=1' and otherwise 'incorrect practice=0'. The providers that correctly managed (treated) children who were classified as having the illnesses at the outset were categorized as 'correct practice=1' and otherwise 'incorrect practice =0'. The providers were observed consulting more than one case and hence, correct assessment and treatment for both conditions were defined as being able to correctly assess and treat all children according to the iCCM guideline.

The tasks of pneumonia assessment according to iCCM guidelines were 'ask for the presence of cough or difficult breathing, check chest-indrawing, and listen for stridor'. The tasks for diarrhea assessment were 'ask for presence of diarrhea, check for presence of blood in the stool, check child restlessness or irritability, and check whether skin pinch goes back slowly or very slowly'.

The explanatory variables were provider characteristics, iCCM training status, presence of supportive supervision and mentorships, and presence of medicines and supplies.

For peer review only - http://bmjopen.bmj.com/site/about/guidelines.xhtml

Data processing and analysis

Primarily, the HEWs module data set and the observation module data set were linked using individual ID and then linked to the health facility assessment data set. Data values were cleaned for inconsistency and incompleteness.

Descriptive statistics were performed to analyze and describe characteristics of providers, other explanatory variables, and the outcome variable. Significant difference between the proportion of providers with the correct knowledge and correct practice was assessed using two-sample z-tests. Logistic regression was used to analyze the association between explanatory variables with knowledge, and actual management practice of providers. Those variables with p-value <0.05 were considered statistically significant and interpreted. All analysis was performed using SPSS version 20 (IBM corporation, New York, USA).

For peer review only

Ethical consideration

Research ethical approval was obtained from EPHI (protocol number SERO-012-8-2016; 001 August 2016), LSHTM (protocol number 11235, June 2016), and Jimma University (protocol number IHRPGD/472/2018, August 2018). Support letter was secured from regional health bureaus and zonal health offices. Informed written consent was taken from providers and for observation, consent was also taken from caregivers. The confidentiality of providers and patient information was maintained through use of codes. Data is used for the intended research purpose and will not share with a third party.

Patient and public involvement

Patient and/or the public were not involved in the design or conduct, or reporting or dissemination plans of this research.

For peer review only - http://bmjopen.bmj.com/site/about/guidelines.xhtml

Results

Characteristics of providers

From the total of 274 HEWs interviewed, 150 have consulted sick children and were included in the know-do gap and actual practice analysis. While, 274 HEWs were used in the knowledge analysis.

Greater than two-thirds (68.2%) of HEWs were less than or equal to 30 years of age and over half had level-3 training (56.2%). Six out of ten (61.3%) of them had work experience of six years and more. The majority (78.8%) of providers had received iCCM training and out of which the larger proportion had received training within 12 months preceding survey date (187 or 86.6%). Though the majority of HEWs had received supervision within the 3 months preceding the survey date (71.9%), only 33.9% had been visited regularly. Supervisions that were focused on the management of pneumonia and diarrhea have been given to greater than two-thirds of the HEWs, and for half (50.3%) the supervision included a component of observing client consultation with the HEW. Furthermore, less than half (46%) of HEWs participated in the performance review clinical mentoring meetings (PRCMM) that were held 6 months preceding survey date (Table 1).

For peer review only

Table 1. Background characteristics of the health extension workers

Variables	Frequency	Percent
Age in years		
≤30	187	68.2
>30	87	31.8
Level of Training[a]		
Level-3	154	56.2
Level-4	120	43.8
Work experience as an HEW in years		
≤5	106	38.7
6-10	63	23.0
>10	105	38.3
iCCM training		

For peer review only - http://bmjopen.bmj.com/site/about/guidelines.xhtml

Yes	216	78.8
No	58	21.2
Received supportive supervision in the last 3 months		
Yes	197	71.9
No	77	28.1
Number of visits (in the last 3 months)		
No visit	77	28.1
One to two	104	38.0
Three and more	93	33.9
Contents of supportive supervision[b]		
Discussing diagnosis and treatment of suspected pneumonia	132	67.0
Discussing diagnosis and treatment of suspected diarrhea	139	70.6
Observing record keeping	187	94.9
Checking the register for consistency and completeness	183	92.9
Checking supplies including manuals, job aids, and request forms	146	74.1
Delivering supplies including manuals, job aids and request forms	123	62.4
Observing client consultation with providers	99	50.3
Providing written feedback	131	66.5
Participated in PRCMM in the last 6 months		
Yes	126	46.0
No	148	54.0
Region		
Amhara	130	47.4
Oromia	71	25.9
SNNP	37	13.5
Tigray	36	13.1

Abbreviations: iCCM, integrated community case management; PRCMM, performance review clinical mentoring meeting; SNNP, southern nations, nationalities, and people

[a]Level of training: Health extension workers (HEW) level of training categories: Level-3 HEWs are those who graduated with a certificate after a one-year pre-service training on the 17 packages of the Health Extension Program. Level-4 HEWs are those who graduated with a diploma after one year of additional education at a technical college.

[b]Out of the total health extension workers that have received supervision in the last 3 months (N=197)

For peer review only - http://bmjopen.bmj.com/site/about/guidelines.xhtml

The HEWs have consulted a total of 685 sick children, with an average of 5 sick children consultations per HEW (SD 1.6). The minimum and maximum consultations were 1 and 6, and the majority 127(84.7%) of HEWs have consulted three and more sick children. Out of the total sick children who visited the HEWs 628(91.7%) were older than 6 months. Three hundred sixteen (46%) sick children had respiratory complaints and 302(44%) had diarrhea related complaints.

Medical equipment, job aids, and medicines availability

Medical equipment and job aids

A total of 121 (82.3%) providers had thermometers, 115(78.2%) had infant scales, 116(78.9%) had weighing slings, 79(53.7%) had tape measures, 69(46.9%) had BP apparatus, 98(66.7%) had stethoscopes, 16(10.9%) had watch or clock, and 102(69.4%) had examination couch. Whereas all of the providers reported that they had MUAC tape, which is used to assess malnutrition.

Regarding job aids, 145(98.6%) had iCCM register for 2-59 months, and 138(93.9%) had chart booklets.

Medicines

Overall, 115 HEWs (71.4%) out of 147 had access to amoxicillin on the date of assessment. Amoxicillin was out of stock for 24 HEWs with an average stock-out day of 37 (SD 31.1). The minimum and maximum days of stock-out were 2 and 95 days, respectively.

Oral rehydration solution (ORS) medicines were available to 137 (93.2%) providers and zinc to 125(85%) providers. ORS was out of stock for 6 providers with an average stock outage day of 22 (SD 34.2). Minimum and maximum stock out days were 1 and 90 days, respectively. Whereas, zinc was out of stock for 19 providers with an average stock outage day of 38 (SD 44.9). The minimum and maximum days for zinc were 1 and 180, respectively. Both ORS and zinc medicines

For peer review only

For peer review only - http://bmjopen.bmj.com/site/about/guidelines.xhtml

were available to 117(79.6%) providers. Paracetamol tablets were available to 94 (63.9%) providers. Paracetamol was out of stock for 31 providers with an average stock outage day of 40 (SD 31.6) with a range of 1-120 days (Table 2).

Table 2. Medical equipment, job aids, and medicine availability (N=147)

Medical equipment, job aids, and Medicines	Frequency (%)
Medical equipment	
Thermometer	121(82.3)
Infant scale	115(78.2)
Weighing sling	116(78.9)
Tape measures	79(53.7)
Blood pressure apparatus	69(46.9)
Stethoscopes	98(66.7)
Watch or clock (timer)	16(10.9)
Examination couch	102(69.4)
Mid-upper-arm circumference tape	147(100.0)
Basic equipment availability index[a]	44(29.9)
Job aids	
iCCM register (2-59 months old children)	145(98.6)
Stock/bin cards to track medicines	97(66.0)
Medicines request and resupply forms	95(64.6)
Chart booklets	138(93.9)
Medicines	
Amoxicillin	115(78.2)
Oral rehydration solution	137(93.2)
Zinc	125(85.0)
Oral rehydration solution and zinc	117(79.6)
Paracetamol	94(63.9)
Amoxicillin, oral rehydration solution and zinc	96(65.3)

[a] Basic equipment availability index: Availability of all of the following for the same provider: thermometer, infant scale, weighing sling, stethoscope, and BP apparatus

Providers knowledge

Out of 274 providers analyzed (Table 3), less than four out of ten had good knowledge of signs and symptoms of pneumonia (35.4%) and diarrhea (20.8%), and the management approaches of

For peer review only - http://bmjopen.bmj.com/site/about/guidelines.xhtml

pneumonia (24.5%), and diarrhea (24.1%). Slightly over a quarter (27%) of providers were knowledgeable about signs and symptoms, and management approaches of both illnesses.

Table 3. Providers knowledge of childhood pneumonia and diarrhea management (N=274)

Knowledge of-	Frequency	%(95% CI)
Pneumonia signs and symptoms		
Good knowledge	97	35.4(29.9-41.2)
Poor knowledge	177	64.6(58.8-70.1)
Pneumonia management approaches		
Good knowledge	67	24.5(19.6-29.8)
Poor knowledge	207	75.5(70.2-80.4)
Pneumonia signs and symptoms and management approaches		
Good knowledge	108	39.4(33.7-45.3)
Poor knowledge	166	60.6(54.7-66.3)
Diarrhea signs and symptoms		
Good knowledge	57	20.8(16.3-25.9)
Poor knowledge	217	79.2(74.1-83.7)
Diarrhea management approaches		
Good knowledge	66	24.1(19.3-29.4)
Poor knowledge	208	75.9(70.6-80.7)
Diarrhea signs and symptoms and management approaches		
Good knowledge	78	28.5(23.4-34.0)
Poor knowledge	196	71.5(66.0-76.6)
Both illnesses sign and symptoms and management approaches		
Good knowledge	74	27.0(22.0-32.5)
Poor knowledge	200	73.0(67.5-78.0)

For peer review only

Know-do gap in child healthcare

There was significant difference between the knowledge of HEWs and their actual practices in the assessment of pneumonia and diarrhea ($p<0.05$ for individual and overall know-do gap scores as in table 4). Greater than one-third (35.7%) of HEWs had good knowledge of pneumonia signs and

For peer review only - http://bmjopen.bmj.com/site/about/guidelines.xhtml

symptoms and only one-fifth of them (19.8%) had actually assessed those symptoms. Though three-fourth of providers know cough or fast breathing as the sign and symptoms of pneumonia, only 47.3% of them have actually asked about cough or difficult breathing. Likewise, 34.9% of providers have described chest-indrawing as the symptom of pneumonia while only 28.6% have assessed chest-indrawing in children.

Six out of ten (61.5%) of providers have good knowledge of diarrhea signs and symptoms, however only 40.2% were able to actually assess those signs and symptoms in children who presented with diarrhea complaints.

Table 4. Know-do gap in the assessment of pneumonia and diarrhea

Assessment	Knowledge (n, %)	Practice (n, %)	Gap (%)	Z test p value
Pneumonia				
Cough or fast breathing	114(76.0)	71(47.3)	28.7	p<0.0001
Chest-indrawing[a]	44(34.9)	36(28.6)	6.3	0.20
Stridor[a]	35(27.8)	19(15.1)	12.7	0.006
Pneumonia index[a]	45(35.7)	25(19.8)	15.9	0.005
Diarrhea				
Presence of diarrhea	126(84.0)	62(41.3)	42.7	p<0.0001
Blood in the stool[b]	39(32.0)	52(42.6)	(10.6)	0.17
Child restlessness or irritability[b]	39(32.0)	46(37.7)	(5.7)	0.26
Pinch the skin of the abdomen[b]	56(45.9)	33(27.0)	18.9	0.001
Diarrhea index[b]	75(61.5)	49(40.2)	21.3	0.001
Overall index[c]	41(38.0)	23(21.3)	16.7	0.002

[a]Out of the health extension workers that have consulted children who were labeled to have a cough or difficult breathing (n=126)
[b] Out of the health extension workers that have consulted children who were labeled to have diarrhea (n=122)
[c]Out of the health extension workers that have consulted children who were labeled to have both cough and diarrhea (n=108)

For peer review only - http://bmjopen.bmj.com/site/about/guidelines.xhtml

Similarly, significant difference was observed between the knowledge and actual practice of providers regarding the treatment of childhood pneumonia and diarrhea ($p<0.05$). Most of the providers (96.2%) have reported that they knew treatment recommendations for pneumonia and diarrhea, but only 27.7% have actually practiced the recommendations for children who were classified as having either pneumonia or diarrhea. On the contrary, large proportion of providers practiced counseling about medications for children who were provided treatments for pneumonia and diarrhea though only a few of them know it as a component of management (Table 5).

Table 5. The know-do gap in the management of pneumonia and diarrhea

Treatment and counseling	Knowledge (n, %)	Practice (n, %)	Gap (%)	Z test p value
Pneumonia				
Prescribe antibiotics[a]	68(88.3)	12(15.6)	72.7	p<0.0001
Counsel on medications[b]	2(16.7)	10(83.3)	(66.6)	0.001
Diarrhea				
Prescribe ORS and zinc[c]	108(93.9)	59(51.3)	42.6	p<0.0001
Counsel on medications[d]	18(30.5)	30(50.8)	(20.3)	0.03
Treatment for pneumonia and diarrhea[e]	126(96.2)	36(27.7)	68.5	p<0.0001

[a]*Out of the health extension workers who have consulted children classified as having pneumonia (N=77)*
[b]*Out of the health extension worker who has provided treatment to pneumonia (N=12)*
[c]*Out of the health extension workers who have consulted children classified as having diarrhea (N=115)*
[d]*Out of the health extension workers who have provided treatment to diarrhea (N=59)*
[e] *Out of the health extension workers who have consulted children who were classified as having either pneumonia or diarrhea (N=130)*

Drivers of providers' knowledge and actual practice

Drivers of knowledge

In the regression analysis, only the participation in PRCMM showed statistically significant associations with the knowledge of the providers (aOR=2.00, 95% CI: 1.09,3.67) (Table 6).

Table 6. Correlates of providers' knowledge

Variables	Knowledge		aOR (95% CI)
	Good(n,%)	Poor(n,%)	
Observations	74	200	
Age in years			

For peer review only - http://bmjopen.bmj.com/site/about/guidelines.xhtml

≤30	43(58.1)	144(72.0)	Ref
>30	31(41.9)	56(28.0)	1.41(0.75-2.64)
Service experience as HEWs in years			
≤5	19(25.7)	87(43.5)	Ref
>5	55(74.3)	113(56.5)	1.78(0.81,3.92)
Level of HEWs			
Level-3	34(45.9)	120(60.0)	Ref
Level-4	40(54.1)	80(40.0)	1.33(0.74,2.39)
iCCM training			
No	12(16.2)	46(23.0)	Ref
Yes	62(83.8)	154(77.0)	0.71(0.29,1.71)
Supervision with discussion on diagnosis and treatment of pneumonia and diarrhea			
No	29(39.2)	103(51.5)	Ref
Yes	45(60.8)	97(48.5)	1.12(0.60,2.11)
Supervision with a component of observing client consultations			
No	38(51.3)	131(65.5)	Ref
Yes	36(48.7)	69(34.5)	1.36(0.73,2.53)
Being participated in PRCMM			
No	29(39.2)	119(59.5)	Ref
Yes	45(60.8)	81(40.5)	2.00(1.09,3.67)**

** P<0.05*

Abbreviations: CI, confidence interval; iCCM, integrated community case management; PRCMM, performance review clinical mentoring meeting; HEW, health extension worker; aOR, adjusted odds ratio

Drivers of actual practice

The logistic regression analysis shown that pneumonia and diarrhea medicines availability was associated with actual management practices of providers (aOR=4.04, 95% CI: 1.48,11.06). The rest of the variables didn't show associations (Table 7).

Table 7. Correlates of the providers' actual practice

Variables	Practice		aOR (95% CI)
	Good(n,%)	Poor(n,%)	
Observations	36	94	
Age in years			
≤30	28(77.8)	70(74.5)	Ref
>30	8(22.2)	24(25.5)	0.61(0.22-1.75)
Service experience as HEWs in years			
≤5	14(38.9)	39(41.5)	Ref
>5	22(61.1)	55(58.5)	1.54(0.59-4.02)
Level of HEWs			
Level-3	20(55.6)	59(62.8)	Ref
Level-4	16(44.4)	35(37.2)	1.52(0.62-3.68)
iCCM training			

For peer review only

For peer review only - http://bmjopen.bmj.com/site/about/guidelines.xhtml

No	30(83.3)	75(79.8)	Ref
Yes	6(16.7)	19(20.2)	1.45(0.39-5.30)
Supervision with discussion on diagnosis and treatment of pneumonia and diarrhea			
No	15(41.7)	42(44.7)	Ref
Yes	21(58.6)	52(55.3)	1.13(0.44-2.87)
Supervision with a component of observing client consultations			
No	23(63.9)	58(61.7)	Ref
Yes	13(36.1)	36(38.3)	0.91(0.37-2.19)
Being participated in PRCMM			
No	19(52.8)	46(48.9)	Ref
Yes	17(47.2)	48(51.1)	0.60(0.24-1.50)
Knowledge index			
Poor knowledge	25(69.4)	67(71.3)	Ref
Good knowledge	11(30.6)	27(28.7)	1.04(0.41-2.64)
Amoxicillin, ORS and zinc availability[a]			
No	6(16.7)	38(40.4)	Ref
Yes	30(83.3)	53(59.6)	4.04(1.48-11.06)*

*** P<0.05*

Abbreviations: CI, confidence interval; iCCM, integrated community case management; PRCMM, performance review clinical mentoring meeting; HEW, health extension worker; aOR, adjusted odds ratio

[a]Out of providers who were assessed for medications availability in the health posts(n=127)

Discussion

The study revealed that low proportion of providers had knowledge of signs and symptoms and management of pneumonia and diarrhea. Similarly, low proportion of providers executed the recommended actions for the assessment and management of the illnesses. There was a significant difference between the knowledge and actual practice of providers. This finding is consistent with a result among other health professionals in Ethiopia (14) and frontline health workers in India (13,17).

Slightly greater than one-third (35.7%) of HEWs had good knowledge of pneumonia signs and symptoms and only one-fifth of them (19.8%) had actually assessed consulting children for those signs and symptoms. The widest gap was observed between knowledge and actual practice of providers related to cough or difficult breathing (76% vs. 47.3%) and stridor (27.8% vs. 15.1%). Besides, the incongruity between the knowledge and actual practices of the providers; the level of

For peer review only - http://bmjopen.bmj.com/site/about/guidelines.xhtml

For peer review only

concordance with the clinical guidelines have remained low among providers. This has a huge impact on clinical care outcomes. Poor adherence to clinical guidelines by providers in the assessment of sick child was also reported in earlier studies from low-and-middle-income countries (34,35) and in Ethiopia(28–30).

Though six out of ten (61.5%) of the providers had good knowledge of signs and symptoms of diarrhea, only four out of ten (40.2%) actually assessed for those symptoms in children who presented with diarrhea complaints. A wide and significant gap was observed between the knowledge and actual practice of the providers related to overall diarrhea assessment (84% vs. 41.3%) and pinch skin of the abdomen (45.9% vs. 27%). On the contrary, a large proportion of providers have practiced assessing the presence of blood in the stool (42.6% vs. 32%), and child restlessness or irritability (37.7% vs. 32%) without reporting on knowledge of the symptoms at the outset. Though clinical guidelines recommend assessment of all symptoms of dehydration, the providers didn't actually practice them (33). This could lead to incorrect diagnosis of sick children and results in missed opportunity to provide life-saving treatment for those in need of it.

Most providers (96.2%) have reported that they know recommended treatment options for pneumonia and diarrhea, but only a quarter of them (27.7%) have actually provided treatments. Earlier studies from Ethiopia and Uganda reported that CHWs had poor knowledge and practice of childhood illnesses management (36–38).

Many factors counter play the lack of knowledge and good practice related to management of sick children. The quality of sick childcare is a function of the providers' knowledge, the effort exerted by the provider to apply the knowledge, and the readiness of facilities to provide quality care in terms of medicines and supplies. In the present study, providers' knowledge and actual practice of pneumonia and diarrhea management were associated with participation in PRCMM and the

For peer review only - http://bmjopen.bmj.com/site/about/guidelines.xhtml

336 availability of medicines at the health posts. Previous study also revealed that clinical coaching is
337 positively associated with good clinical practice among CHWs (39). The odds of good knowledge
338 of pneumonia and diarrhea assessment and management was two times more likely in providers
339 who participated in PRCMM. While, the odds of better correct practice was four times more likely
340 in providers who had medicines for the treatment of childhood pneumonia and diarrhea. Other
341 variables including the providers' characteristics, receiving clinical training, and supportive
342 supervision did not affect knowledge and practice of HEWs. Though most of providers were
343 trained in iCCM, they scored low in the knowledge and practice for assessment and treatment of
344 childhood pneumonia and diarrhea implying that in-serving trainings are necessary but not
345 sufficient drivers of correct practice (40). In another study, providers' level of education, work
346 experience, presence of supervision, and guidelines were associated with CHWs knowledge of
347 childhood illness management (37,38).

348 The present study finding have policy and practice implications. Clinical training interventions
349 that lacked adequate practical skill component and absence of refresher trainings are cause for poor
350 practitioner's performance. While high-quality in-service training coupled with mentoring,
351 supervisions, medicines, and material support are crucial to improve quality of care(41–43).
352 Though training had high coverage in the study area, providers had shortage of medicines,
353 supplies, and technical support. Supervisions and mentoring focused on management of childhood
354 pneumonia and diarrhea were limited. In-service training and supervision were not associated with
355 providers' knowledge and actual practices signaling a look at other approaches that improves
356 quality of training and supervisions.

357 This is the first study that examined the know-do gap in childhood pneumonia and diarrhea care
358 among health extension workers in Ethiopia. Though our findings are limited by a small sample

For peer review only - http://bmjopen.bmj.com/site/about/guidelines.xhtml

size, the study has covered the four most populous regions of the country and is representative of the different settings in rural Ethiopia.

Conclusions

We have reported a huge know-do gap in the assessment and management of childhood pneumonia and diarrhea among the health extension workers in Ethiopia. The finding affirm that knowledge-based training is necessary but not sufficient to influence the clinical performance (actual practice) of providers. The know-do gap of pneumonia and diarrhea management was determined by the presence of clinical mentoring and performance reviews and the availability of medicines. Therefore, an effort should be made to close the gap between theoretical knowledge and practice. The health extension workers should be supported with mentoring, performance reviews, and the supply of medicines and commodities.

Acknowledgments

The authors acknowledge the administrative hierarchies and the research participants in the study area for their cooperation throughout the research process, and the time they devoted to the study in providing required information.

Authors' contributions

All authors made a significant contribution to the work reported, whether, that is, in the conception, study design, execution, acquisition of data, analysis, and interpretation, or in all these areas. DWD wrote the first draft of the manuscript, and MAW and MW revised the manuscript. All authors gave final approval of the version to be published, have agreed on the journal to which the article has been submitted, and agreed to be accountable for all aspects of the work. All authors read and approved the final manuscript.

For peer review only - http://bmjopen.bmj.com/site/about/guidelines.xhtml

Funding

This research project was funded by Bill & Melinda Gates Foundation with a grant to London School of Hygiene & Tropical Medicine (INV-009691). The funder has no role in the study design, collection, management, analysis or interpretation of data.

Data availability

The datasets used and/or analyzed for the current study are not openly available because data are part of the ongoing research project and available from the corresponding author upon reasonable request (E-mail: dave86520@gmail.com or dawit.daka@ju.edu.et)

Competing interests

All authors declare that they have no competing interests.

Patient consent for publication

Not required

References

1. United Nations Children's Fund (UNICEF). Pneumonia and diarrhoea: Tackling the deadliest diseases for the world's poorest children [Internet]. United Nations Children's Fund. 2012. 1–86 p. Available from: http://www.unicef.org/eapro/Pneumonia_and_Diarrhoea_Report_2012.pdf

2. United Nations Children's Fund (UNICEF). One is too many: Ending child deaths from pneumonia and diarrhoea [Internet]. 2016. 25 p. Available from: https://data.unicef.org/wp-content/uploads/2016/11/UNICEF-Pneumonia-Diarrhoea-report2016-web-version_final.pdf

For peer review only

For peer review only - http://bmjopen.bmj.com/site/about/guidelines.xhtml

3. IVAC at Johns Hopkins Bloomberg School of Public Health. Pneumonia and Diarrhea Progress Report 2018 [Internet]. 2018. Available from: http://link.springer.com/10.1023/A:1020102612073%0Ahttp://apps.who.int/gb/ebwha/pdf_files/WHA66/A66_R10-en.pdf%0Ahttp://www.who.int/diabetes/country-profiles/nga_en.pdf?ua=1

4. International Vaccine Access Center (IVAC). Pneumonia and Diarrhea Progress Report 2020 [Internet]. John Hopkins Bloomberg School of Public Health. 2020. Available from: https://resourcecentre.savethechildren.net/pdf/ivac_pdpr_2020.pdf/

5. Nanyonjo A, Ssekitooleko J, Counihan H, Makumbi F, Tomson G, Källander K. Impact of an integrated community case management programme on uptake of appropriate diarrhoea and pneumonia treatments in Uganda: A propensity score matching and equity analysis study. Int J Equity Health [Internet]. 2015;14:74. Available from: http://dx.doi.org/10.1186/s12939-015-0202-y

6. Winskill P, Mousa A, Oresanya O, Counihan H, Okell LC, Walker PG. Does integrated community case management (iCCM) target health inequities and treatment delays? Evidence from an analysis of Demographic and Health Surveys data from 21 countries in the period 2010 to 2018. J Glob Health. 2021;11:040113.

7. Young M, Wolfheim C, Marsh DR, Hammamy D. World health organization/United Nations children’s fund joint statement on integrated community case management: An equity-focused strategy to improve access to essential treatment services for children. Am J Trop Med Hyg. 2012;87(SUPPL.5):6–10.

8. World Health Organization. Integrated Management of Childhood Illness (IMCI): Chart

For peer review only

Booklet. Geneva, Switzerland: World Health Organization. 2014.

9. Kallander K, Burgess DH, Qazi SA. Early identification and treatment of pneumonia: A call to action. Lancet Glob Heal. 2016;4(1):e12–3.

10. HEFDC Group. Integrated community case management (iCCM) in sub-Saharan Africa: Successes and challenges with access, speed and quality. Thematic Review Report. 2018.

11. Bagonza J, Kibira SPS, Rutebemberwa E. Performance of community health workers managing malaria , pneumonia and diarrhoea under the community case management programme in central Uganda : a cross sectional study. Malar J. 2014;13:367.

12. Chinbuah MA, Abbey M, Kager PA, Gyapong M, Nonvignon J, Ashitey P, et al. Assessment of the adherence of community health workers to dosing and referral guidelines for the management of fever in children under 5 years: a study in Dangme West District, Ghana. Int Health. 2013;5:148–56.

13. Ray Saraswati L, Baker M, Mishra A, Bhandari P, Rai A, Mishra P, et al. 'Know-Can' gap: gap between knowledge and skills related to childhood diarrhoea and pneumonia among frontline workers in rural Uttar Pradesh, India. Trop Med Int Heal. 2020;25(4):454–66.

14. Gage AD, Kruk ME, Girma T, Lemango ET. The know-do gap in sick child care in Ethiopia. PLoS One. 2018;13(12):e0208898.

15. Tielsch JM. The Know, Do, and Quality Gaps in International Maternal and Child Health Care Interventions. JAMA Pediatr [Internet]. 2015 Apr 1;169(4):313–4. Available from: https://doi.org/10.1001/jamapediatrics.2014.3741

For peer review only - http://bmjopen.bmj.com/site/about/guidelines.xhtml

16. Kebede D, Zielinski C, Mbondji PE, Sanou I, Asamoah-odei E, Soumbey-alley EW, et al. Improving the availability, quality and use of health information, research evidence and knowledge to strengthen health systems. World Heal Organ. 2010;(1 2):53–64.

17. Mohanan M, Vera-Hernández M, Das V et al. The Know-Do Gap in Quality of Health Care for Childhood Diarrhea and Pneumonia in Rural India. Jama Pediatr. 2015;169(4):349–57.

18. Mungai IG, Baghel SS, Soni S, Vagela S, Sharma M, Diwan V, et al. Identifying the know-do gap in evidence-based neonatal care practices among informal health care providers—a cross-sectional study from Ujjain, India. BMC Health Serv Res. 2020;20:966.

19. Wanduru P, Tetui M, Tuhebwe D, Ediau M, Okuga M, Nalwadda C, et al. The performance of community health workers in the management of multiple childhood infectious diseases in Lira, northern Uganda – a mixed methods cross-sectional study. Glob Health Action [Internet]. 2016 Dec 1;9:33194. Available from: https://doi.org/10.3402/gha.v9.33194

20. Rabbani F, Perveen S, Aftab W, Zahidie A, Sangrasi K, Qazi SA. Health workers' perspectives, knowledge and skills regarding community case management of childhood diarrhoea and pneumonia: A qualitative inquiry for an implementation research project "nigraan" in District Badin, Sindh, Pakistan. BMC Health Serv Res [Internet]. 2016;16:462. Available from: http://dx.doi.org/10.1186/s12913-016-1699-5

21. Izudi J, Anyigu S, Ndungutse D. Adherence to Integrated Management of Childhood Illnesses Guideline in Treating South Sudanese Children with Cough or Difficulty in

For peer review only - http://bmjopen.bmj.com/site/about/guidelines.xhtml

Breathing. Menahem S, editor. Int J Pediatr [Internet]. 2017;2017:5173416. Available from: https://doi.org/10.1155/2017/5173416

22. Miller NP, Amouzou A, Tafesse M, Hazel E, Legesse H, Degefie T, et al. Integrated Community Case Management of Childhood Illness in Ethiopia : Implementation Strength and Quality of Care. Am J Trop Med Hyg. 2014;91(2):424–34.

23. Sinyangwe C, Graham K, Nicholas S, King R, Mukupa S, Källander K, et al. Assessing the quality of care for pneumonia in integrated community case management: A cross-sectional mixed methods study. PLoS One. 2016;11(3):e0152204.

24. Baynes C, Mboya D, Likasi S, Maganga D, Pemba S, Baraka J, et al. Quality of sick child-care delivered by community health workers in Tanzania. Int J Heal Policy Manag [Internet]. 2018;7(12):1097–109. Available from: https://doi.org/10.15171/ijhpm.2018.63

25. Berhanu A, Alemayehu M, Daka K, Binu W, Suleiman M. <p>Utilization of Integrated Community Case Management of Childhood Illnesses at Health Posts in Southern Ethiopia</p>. Pediatr Heal Med Ther. 2020;Volume 11:459–67.

26. Assefa Y, Gelaw YA, Hill PS, Taye BW, Damme W Van. Community health extension program of Ethiopia , 2003 – 2018 : successes and challenges toward universal coverage for primary healthcare services. Global Health [Internet]. 2019;15:24. Available from: https://globalizationandhealth.biomedcentral.com/track/pdf/10.1186/s12992-019-0470-1

27. Legesse H, Degefie T, Hiluf M, Sime K, Tesfaye C, Abebe H, et al. National scale-up of integrated community case management in rural Ethiopia: Implementation and early lessons learned. Ethiop Med J. 2014;52(December):15–26.

For peer review only - http://bmjopen.bmj.com/site/about/guidelines.xhtml

28. Wolde D, Daka, Wordofa MA, Woldie M, Persson Å, Berhanu D. Quality of clinical assessment and management of sick children by Health Extension Workers in four regions of Ethiopia : A cross-sectional survey. PLoS One. 2020;15(9):e0239361.

29. Beyene H, Hailu D, Tadele H, Persson LÅ, Berhanu D. Insufficient referral practices of sick children in Ethiopia shown in a cross-sectional survey. Acta Paediatr Int J Paediatr. 2020;109(9):1867–74.

30. Getachew T, Mekonnen S, Yitayal M, Persson LÅ, Berhanu D. Health Extension Workers' diagnostic accuracy for common childhood illnesses in four regions of Ethiopia: a cross-sectional study. Acta Paediatr Int J Paediatr [Internet]. 2019;108(11):2100–6. Available from: https://onlinelibrary.wiley.com/doi/epdf/10.1111/apa.14888

31. Berhanu D, Okwaraji YB, Belayneh AB, Lemango ET, Agonafer N, Birhanu BG et al. Protocol for the evaluation of a complex intervention aiming at increased utilization of primary child health services in Ethiopia: A before and after study in intervention and comparison areas. BMC Health Serv Res. 2010;20:339.

32. Organization WH. Health facility survey : tool to evaluate the quality of care delivered to sick children attending outpatient facilities [Internet]. Geneva PP - Geneva: World Health Organization; Available from: https://apps.who.int/iris/handle/10665/42643

33. Ministry of Health of Ethiopia. The Integrated Community Case Management (iCCM) Chart Booklet Ethiopia [Internet]. 2014. Available from: http://lifesavingcommodities.org/wp-content/uploads/2014/08/iCCM-English-Chartbooklet-August-2013.pdf

34. Gilroy KE, Callaghan-koru JA, Cardemil C V, Nsona H, Amouzou A, Mtimuni A, et al.

For peer review only

For peer review only - http://bmjopen.bmj.com/site/about/guidelines.xhtml

Quality of sick child care delivered by Health Surveillance Assistants in Malawi. Health Policy Plan. 2013;28:573–85.

35. Kruk RME, Gaga AD, Mbaruku GM, Leslie HH. Content of Care in 15,000 Sick Child Consultations in Nine Lower-Income Countries. Heal Serv Res [Internet]. 2018;12842. Available from: https://doi.org/10.1111/1475-6773.12842).

36. Bagonza J, Kibira SPS, Rutebemberwa E. Performance of community health workers managing malaria, pneumonia and diarrhoea under the community case management programme in central Uganda: A cross sectional study. Malar J. 2014;13(1):1–10.

37. Befekadu A, Yitayal M. Knowledge and practice of health extension workers on drug provision for childhood illness in west Gojjam, Amhara, Northwest Ethiopia. BMC Public Health [Internet]. 2020;20:496. Available from: https://doi.org/10.1186/s12889-020-08602-y

38. Wanduru P, Tetui M, Tuhebwe D, Ediau M, Okuga M, Nalwadda C, et al. The performance of community health workers in the management of multiple childhood infectious diseases in Lira, northern Uganda - A mixed methods cross-sectional study. Glob Health Action. 2016;9(1).

39. Shiroya-Wandabwa M, Kabue M, Kasungami D, Wambua J, Otieno D, Waka C, et al. Coaching community health volunteers in integrated community case management improves the care of sick children under-5: Experience from Bondo, Kenya. Int J Integr Care. 2018;18(4):1–11.

40. Leslie HH, Gage A, Nsona H, Hirschhorn LR, Kruk ME. Training and supervision did not meaningfully improve quality of care for pregnant women or sick children in sub-Saharan

For peer review only

Africa. Health Aff. 2016;35(9):1716–24.

41. Leslie HH, Gage A, Nsona H, Hirschhorn LR, Kruk ME. Training And Supervision Did Not Meaningfully Improve Quality Of Care For Pregnant Women Or Sick Children In Sub-Saharan Africa. Health Aff [Internet]. 2016 Sep 1;35(9):1716–24. Available from: https://doi.org/10.1377/hlthaff.2016.0261

42. Bluestone J, Troncoso E, Fitzgerald L, Nagbe L, Tetteh G, Hellar A, et al. Three Pivots for Improving Health Care Provider Performance. Glob Heal Sci Pract [Internet]. 2022 Feb 28;10(1):e2100625. Available from: http://www.ghspjournal.org/content/10/1/e2100625.abstract

43. Desta BF, Beshir IA, Tefera BB, Argaw MD, Demeke HZ, Kibret MA. Does frequency of supportive supervisory visits influence health service delivery?—Dose and response study. PLoS One [Internet]. 2020 Jun 16;15(6):e0234819. Available from: https://doi.org/10.1371/journal.pone.0234819

For peer review only

For peer review only - http://bmjopen.bmj.com/site/about/guidelines.xhtml

BMJ Open

Know-do gap for sick child care and drivers of knowledge and practice among health extension workers in four regions of Ethiopia

Journal:	*BMJ Open*
Manuscript ID	bmjopen-2022-069698.R1
Article Type:	Original research
Date Submitted by the Author:	10-Apr-2023
Complete List of Authors:	Daka, Dawit; Jimma University, Health Policy and Management<br>Wordofa, Muluemebet; Jimma University, Department of Population & Family Health<br>Woldie, Mirkuzie; Jimma University; Jimma University, Health Policy and Management
**Primary Subject Heading**:	Health services research
Secondary Subject Heading:	Health services research
Keywords:	Community child health < PAEDIATRICS, Quality in health care < HEALTH SERVICES ADMINISTRATION & MANAGEMENT, INFECTIOUS DISEASES, Public health < INFECTIOUS DISEASES

SCHOLARONE™
Manuscripts

I, the Submitting Author has the right to grant and does grant on behalf of all authors of the Work (as defined in the below author licence), an exclusive licence and/or a non-exclusive licence for contributions from authors who are: i) UK Crown employees; ii) where BMJ has agreed a CC-BY licence shall apply, and/or iii) in accordance with the terms applicable for US Federal Government officers or employees acting as part of their official duties; on a worldwide, perpetual, irrevocable, royalty-free basis to BMJ Publishing Group Ltd ("BMJ") its licensees and where the relevant Journal is co-owned by BMJ to the co-owners of the Journal, to publish the Work in this journal and any other BMJ products and to exploit all rights, as set out in our licence.

The Submitting Author accepts and understands that any supply made under these terms is made by BMJ to the Submitting Author unless you are acting as an employee on behalf of your employer or a postgraduate student of an affiliated institution which is paying any applicable article publishing charge ("APC") for Open Access articles. Where the Submitting Author wishes to make the Work available on an Open Access basis (and intends to pay the relevant APC), the terms of reuse of such Open Access shall be governed by a Creative Commons licence – details of these licences and which Creative Commons licence will apply to this Work are set out in our licence referred to above.

Other than as permitted in any relevant BMJ Author's Self Archiving Policies, I confirm this Work has not been accepted for publication elsewhere, is not being considered for publication elsewhere and does not duplicate material already published. I confirm all authors consent to publication of this Work and authorise the granting of this licence.

For peer review only

Know-do gap for sick child care and drivers of knowledge and practice among health extension workers in four regions of Ethiopia

Dawit Wolde Daka[1*], Muluemebet Abera Wordofa[2], and Mirkuzie Woldie[1,3]

[1]Faculty of Public Health, Department of Health Policy and Management, Jimma University, Jimma, Ethiopia

[2]Faculty of Public Health, Department of Population and Family Health, Jimma University, Jimma, Ethiopia

[3] Fenot Project, University of British Columbia, School of Public Health and Population, Addis Ababa, Ethiopia

*Corresponding Author

E-mail: dave86520@gmail.com

https://orcid.org/0000-0001-5465-6345

For peer review only - http://bmjopen.bmj.com/site/about/guidelines.xhtml

Abstract

Background

Though efforts were made to expand community-based sick child healthcare in Ethiopia, the quality of care provided remained low. Improving quality of care requires understanding providers' knowledge of appropriate care and their actual execution of tasks. This study examined the gap between what health extension workers(HEWs) knew and did during the management of sick children in Ethiopia.

Methods

A cross-sectional study was conducted from December 2018 to February 2019 among 150 HEWs in four regions using data from providers' interviews, observations of actual practices, and facility assessments. We compared providers' knowledge and performance of childhood pneumonia and diarrhea management. Know-do gap is difference in proportion between knowledge and actual practice of HEWs. Logistic regression was used to identify predictors of knowledge and actual practice.

Results

The providers' knowledge and actual practices on pneumonia and diarrhea assessment were 38%, and 21.3% respectively. Providers' correct knowledge ranged from 27.8% to 76.0% for signs and symptoms of pneumonia, and 32.0% to 84% for dehydration signs. Their actual practices ranged from 15.1% to 47.3% for pneumonia, and 27.0% to 42.6% for dehydration. Similarly, the correct knowledge and actual practices for pneumonia and dehydration management were 88.3% vs 15.6% and 93.9% vs 51.3%, respectively. There was significant know-do gap in assessments (16.7%, p=0.002) and management of childhood conditions (68.5%, p<0.0001). Mentorships were

For peer review only - http://bmjopen.bmj.com/site/about/guidelines.xhtml

associated with providers' knowledge of clinical management, while medicines availability was associated with their actual management practice.

Conclusions

While knowledge and actual practice for assessment and management of pneumonia and dehydration ranged from very low to high, what is more concerning is the huge know-do gap among HEWs. Our findings suggest that knowledge-based training is necessary but not sufficient for ensuring correct assessment and management of sick children by HEWs. Continuous support through mentorships and the supply of commodities are critically needed.

Keywords: Knowledge, actual practice, Know-do gap, pneumonia, diarrhea, assessment, management, health extension workers, Ethiopia

Strength and limitations

- This is the first study that examined the know-do gap of sick childcare among health extension workers in Ethiopia
- The questionnaires were adapted from validated tools and observations of sick child consultations were performed by clinicians who had received refresher training on integrated community case management (iCCM)
- Though the findings are limited by sample size, the study has covered the four largest regions of the country and is representative of the different settings in rural Ethiopia.
- Moreover, the observation method could be influenced by the Hawthorne effect through masking the actual practice due to a change in the professional behavior of the health extension workers when being observed. However, the findings revealed that the providers have performed less, and this might even be worse in normal situations.

For peer review only - http://bmjopen.bmj.com/site/about/guidelines.xhtml

Introduction

Pneumonia and diarrhea remain the two major childhood illnesses that contributed to the larger portion of under-five mortality worldwide (1,2). Globally, over 1.23 million children died of pneumonia and diarrhea before reaching their fifth birthday in 2018. Seventy percent of all pneumonia and diarrhea deaths in children under the age of five years have occurred in 15 countries of the world alone, and Ethiopia is one among them. In Ethiopia, 44, 692 under-five children's deaths were attributed to pneumonia and diarrhea in 2017 which accounted for 13.6 deaths per 1000 live births. Out of sick children with suspected pneumonia, only 31% were taken to an appropriate care provider and 7% received the recommended antibiotics. Similarly, of those under five children with diarrhea, 30% have received oral rehydration solution (ORS) and 33% were provided with zinc supplements (3,4).

The integrated community case management (iCCM) approach to common childhood illnesses is a key strategy for improving the treatment of potentially life-threatening illnesses in children (5–7). The iCCM guideline recommends that all children 2-59 months of age are first assessed for general danger signs, then for cough or difficult breathing, diarrhea, fever, ear problem, acute malnutrition, and anemia. Then children are classified based on assessment results and treated according to the standard of care(8). Adherence to guidelines by front-line providers is critical to improving the quality of care for sick children and improving treatment outcomes (9). However, studies have shown that providers' adherence to the clinical standards of sick children assessment and management is low in most low- and middle-income settings (10–12) and a significant know-do gap in clinical practices was common among the health care providers(13,14).

For peer review only - http://bmjopen.bmj.com/site/about/guidelines.xhtml

The know-do gap, failure to apply existing knowledge to improve health, has a huge impact on the effort made towards reducing under-five mortalities attributed to poor quality or substandard care. It results in inconsistencies in care and inefficiencies in workflows. Though much is known about the evidence-based interventions related to maternal and child healthcare globally, it is not practiced as intended and the practices vary across the different settings (15). Most of the know-do gap is a result of inadequate skilled human, material, and financial resources(16).

Often providers express that they know about the assessment and management of sick children, but only a few of them actually perform those tasks according to the standard. A study conducted in Ethiopia among health facility level providers revealed that there was a gap between the knowledge of providers and their actual performance in child healthcare. The gap was determined by the provider type and the availability of key equipment, and higher provider knowledge scores were associated with better performance in managing sick children(14). Similarly, a study done in rural India also revealed that there was a large know-do gap in pneumonia and diarrhea management by the healthcare providers (17) and a know-do gap in neonatal care among informal healthcare providers(18). The knowledge and actual practices of providers are related to various factors including background characteristics of providers (sex, age, working experience, level of education), presence of medical equipment and medicines, community support, and the presence of trainings, mentoring, and supportive supervision(11,13,19–21). If community health workers are supported adequately, they can assess and treat sick children according to the protocol in the majority of the cases (22–24).

Narrowing the know-do gap is critical to attaining child survival goals and the targets set under the Global Action Plan for the Prevention and Control of Pneumonia and Diarrhea (GAPPD) in 2025. One of the core interventions outlined in the action plan was the appropriate management of

For peer review only - http://bmjopen.bmj.com/site/about/guidelines.xhtml

sick children who are at risk of serious illnesses or death due to these two diseases (pneumonia and diarrhea). It is aimed to increase treatment coverage for children with suspected pneumonia including care by an appropriate healthcare provider and antibiotics, and treatment coverage for children with diarrhea including treatments with ORS and zinc supplements to 90% (4).

In Ethiopia, the integrated community case management services implementation was started in 2010 as part of the health extension program(22,25). Currently, the program is implemented in more than 18,000 health posts nationally (26). Despite huge investments and high coverage of clinical training (22,27), studies revealed that the quality of care is low in the health posts of Ethiopia in terms of sick children assessment, classification, treatment, and referral practices(28–30). On the other hand, little is known about the know-do gap in sick child assessment and management, and the factors that cause it among health extension workers(HEWs) in Ethiopia. Therefore, this paper aimed to examine the level of agreement between the knowledge the HEWs had about assessment and management and their actual practice of childhood pneumonia and diarrhea.

Methods

Study setting and period

The study was conducted in the health posts of 52 districts across the four populous regions of Ethiopia: Oromia, Amhara, Southern Nations Nationalities and People's (SNNP), and Tigray from December 2018 to February 2019. The four regions and the districts were selected by the Ministry of Health, Regional Health Bureaus, and the implementing partners in 2016 for the planned intervention. This paper was part of the larger Dagu end-line survey, a survey that aimed to estimate the effectiveness of the optimizing health extension program (OHEP) in Ethiopia and the

For peer review only - http://bmjopen.bmj.com/site/about/guidelines.xhtml

baseline survey was conducted from December 2016 to February 2017. The protocol for OHEP evaluation was published elsewhere (31).

Study design and participants

A facility-based cross-sectional study was conducted among health extension workers that fulfilled the inclusion criteria. Two-stage stratified sampling strategy was used to select the study participants from the districts. In the first stage enumeration areas (EAs) were selected from the study districts using the list of EAs from the 2007 Ethiopian Housing and Population Census as the sampling frame. The cumulative population size to clusters across the study areas was calculated and 200 EAs were selected with a probability proportional to population size (PPS). Each EA formed a cluster and each cluster constituted a primary sampling unit. In the second stage, all health posts serving the catchment population of the selected EAs were included in the study. Similarly, all HEWs who were present during the survey period were included in the study.

Measurement

Structured interviews, observation, and health post-assessment questionnaires were used to collect data. The questionnaires were developed based on the World Health Organization (WHO) tool for Integrated Management of Childhood Illnesses (IMCI) sick child quality assessment in primary healthcare facilities(32) and the national iCCM guidelines(33). The health provider's interview questions included items such as background characteristics, iCCM training, supportive supervision, clinical mentorships, and knowledge of assessment and management of sick children 2-59 months. The observation questionnaire captured data on the reason for visits, assessment, classification, treatment, referral, and counseling. The facility assessment tool had sections of

For peer review only - http://bmjopen.bmj.com/site/about/guidelines.xhtml

health posts infrastructure, medical equipment, medicines, and job aids. All the questionnaires were uploaded on tablets (CSPro 7.1) for data collection.

Data collectors with a qualification of bachelor's degree in health science were recruited and involved in data collection. Likewise, supervisors with a health background and qualifications with a bachelor of science and above have participated. Data collectors and supervisors were trained for 10 days on the questionnaires, data collection techniques, how to use tablets, and research ethics. Clinical observations of providers were conducted following interviews and facility assessments. The supervisors have monitored the daily data collection process at the field level and provided support. Moreover, a data manager at the central office monitored data on the server and provided feedback to the field team.

Study variables

The outcome variable was the know-do gap of child care related to pneumonia and diarrhea assessment and management. The providers' knowledge data were obtained from interviews, and the actual practice data were collected through observation by clinicians. Know-do gap was the difference between the knowledge of HEWs about the signs and symptoms and management of pneumonia and diarrhea and their ability to correctly assess those signs and symptoms when children are presented with the illnesses. A provider was regarded as having a know-do gap in the following conditions: (a) if correctly named the signs and symptoms of the illnesses but failed to actually identify them; (b) if unable to correctly named the signs and symptoms of the illnesses but able to identify them, and (c) if unable to correctly named the signs and symptoms of the illnesses as well as unable to correctly identify them. On the other hand, if the provider correctly named the signs and symptoms, and also correctly identified them, then he/she was regarded as

For peer review only - http://bmjopen.bmj.com/site/about/guidelines.xhtml

with no know-do gap. Similarly, the above conditions were applied to the know-do gap of pneumonia and dehydration management by the providers.

A total of 11 knowledge items were used, each having ‘yes=1’ and ‘no=0’ responses. Providers were asked to respond to questions without prompts and the responses were recorded from the pre-defined list. Then the interviewer recorded the responses of the providers. The knowledge questions were: what are the main signs and symptoms of pneumonia in children 2-59 months (3 responses)? When a child presents with signs and symptoms of pneumonia, what initial measures do you take (2 responses)? What are the main signs and symptoms of diarrhea in children 2-59 months (4 responses)? When a child presents with signs and symptoms of diarrhea, what initial measures do you take (2 responses)?. The initial measures taken by providers for pneumonia have included prescribing antibiotics and counseling on medications, and for diarrhea, the actions were providing ORS and zinc, and counseling on medications.

We have constructed the knowledge index based on the Ethiopian university students’ grading system with a few modifications as there was no validated standard to evaluate providers’ knowledge. The Ethiopian university grading system is excellent [85-100%], very good [75 to 85%), good [60-75%), satisfactory [50-60%), unsatisfactory [45,50%), and very poor (below 45%). The providers who have scored 60% and above were categorized as having ‘Good knowledge/knowledgeable=1’, and otherwise ‘poor knowledge/not knowledgeable=0’.

Hence, the HEWs were labeled knowledgeable if they have correctly responded to ≥60% of the knowledge items (3 and more items out of 5 for pneumonia; 4 and more items out of 6 for diarrhea; 7 and more items out of 11 for average knowledge). All scores below 60% were regarded as poor knowledge for the individual and average scores.

For peer review only - http://bmjopen.bmj.com/site/about/guidelines.xhtml

The ability to correctly assess and manage children (the actual practice of providers) for pneumonia and diarrhea index was constructed. The providers who correctly assessed children that presented with respiratory and diarrhea complaints according to the iCCM guidelines were categorized as 'correct practice for assessment=1' and otherwise 'incorrect practice for assessment=0'. Likewise, the providers who correctly treated children who have been classified as having pneumonia or diarrhea at the outset according to the iCCM guidelines were categorized as 'correct practice for treatment=1' and otherwise 'incorrect practice for treatment=0'. The providers were observed consulting more than one case and hence, correct assessment and treatment for both conditions were defined as being able to correctly assess and treat all children according to the iCCM guideline.

The tasks of pneumonia assessment according to the iCCM guidelines were 'ask for the presence of cough or difficult breathing, check chest-indrawing, and listen for stridor'. The tasks for diarrhea assessment were 'ask for the presence of diarrhea, ask the presence of blood in the stool, check child restlessness or irritability, and check whether skin pinch goes back slowly or very slowly'. The task of pneumonia treatment was providing antibiotics to sick children classified as having pneumonia, and the task of diarrhea treatment was providing ORS and zinc to those sick children who were classified as having diarrhea. The providers that correctly assessed children for pneumonia and diarrhea were categorized as 'correct practice for assessment=1' and otherwise 'incorrect practice for assessment=0'. Those providers who treated all children who were classified as having pneumonia, and diarrhea with antibiotics, and ORS and Zinc were labeled as 'correct practice for treatment=1' and the providers who didn't provide recommended treatments in either of the conditions were labeled as 'incorrect practice for treatment=0'.

For peer review only - http://bmjopen.bmj.com/site/about/guidelines.xhtml

The explanatory variables of knowledge and actual practices were provider characteristics (age, working experience as HEWs, training level of HEWs (level-3 and level-4)), iCCM training status, presence of supportive supervision and mentorships, and presence of medicines and supplies. Level-3 HEWs are those who graduated with a certificate after a one-year pre-service training on the 17 packages of the health extension program, and level-4 HEWs are those who have level-3 certificate and got one additional year training at a technical college(34,35). The 17 packages of the health extension program are categorized into four components or areas as (a) Family health services (maternal and child health, family planning, immunization, adolescent reproductive health, nutrition); (b) Disease prevention and control (HIV/AIDS, Tuberculosis, malaria, first aid); (c) Hygiene and environmental sanitation (proper and safe excreta disposal, proper and safe solid and liquid waste management, water supply safety measures, food hygiene and safety measures, healthy home environment, arthropods and rodents control, personal hygiene); and (d) Health education and communication(35).

Data processing and analysis

Primarily, the HEWs module data set and the observation module data set were linked using individual ID and then linked to the health facility assessment data set. The health facility assessment data included information on health post infrastructure, availability of medical equipment, medicines, and job aids. Data values were cleaned for inconsistency and incompleteness. Coding and re-coding of variables were done, and the indices were computed as appropriate.

Descriptive statistics were performed to analyze and describe the characteristics of providers, other explanatory variables, and the outcome variable. A 95% confidence interval was constructed for the outcome variable and some of the explanatory variables. To assess whether there exist

For peer review only - http://bmjopen.bmj.com/site/about/guidelines.xhtml

significant differences between the proportion of providers with the correct knowledge and correct practice, we have conducted two-sample z-tests for differences in proportions. Bivariate logistic regression was used to select candidate explanatory variables for multiple variable logistic regression at p-value<0.2 and multiple variable logistic regression was used to analyze the association between explanatory variables with knowledge, and actual management practice of the providers. The variables considered in this analysis were provider age, years of experience, level of education (level-3 and level-4), iCCM training status, presence of supportive supervision and mentorships, and presence of medicines and supplies. Those variables with a p-value<0.05 were considered statistically significant. All analysis was performed using SPSS version 20 (IBM corporation, New York, USA).

Ethical consideration

Research ethical approval was obtained from the Ethiopian Public Health Institute (protocol number SERO-012-8-2016; 001 August 2016), the London School of Hygiene and Tropical Medicine (protocol number 11235, June 2016), and Jimma University (protocol number IHRPGD/472/2018, August 2018). A support letter was secured from the regional health bureaus and zonal health offices. Informed written consent was taken from providers and for observation, consent was also taken from caregivers. For caregivers who were unable to read and write, witness was presented and signatures appended on the consent form. The confidentiality of providers and patient information was maintained properly through the use of codes and computer-based data were secured with passwords. Data will be used for the intended research purpose and will not be shared with a third party.

Patient and public involvement

For peer review only - http://bmjopen.bmj.com/site/about/guidelines.xhtml

Patient and/or the public were not involved in the design or conduct, or reporting or dissemination plans of this research.

Results

Description of background characteristics

From the total of 274 HEWs interviewed, 150 examined sick children and were included in the know-do gap and actual practice analysis. While, 274 HEWs were used in the knowledge analysis.

Greater than two-thirds (68.2%) of the HEWs were less than or equal to 30 years of age and over half had level-3 training or certificates (56.2%). Six out of ten (61.3%) of the HEWs had work experience of six years and more. The majority (78.8%) of providers had received iCCM training and out of which the larger proportion had received the training within the 12 months preceding the date of the survey (187 or 86.6%). Though the majority of the HEWs had received supervision within the 3 months preceding the survey date (71.9%), only 33.9% had been visited regularly. Supervisions that were focused on the management of pneumonia and diarrhea have been given to greater than two-thirds of the HEWs, and for half (50.3%) the supervision included a component of observing client consultation with the HEW. Furthermore, less than half (46%) of the HEWs participated in the performance review clinical mentoring meetings (PRCMM) that were held 6 months preceding the survey date (Table 1).

Table 1. Background characteristics of the health extension workers in four regions of Ethiopia, December 2018 to February 2019

Variables	n(%)
Age in years	
≤30	187(68.2)
>30	87(31.8)

For peer review only - http://bmjopen.bmj.com/site/about/guidelines.xhtml

Level of Training[a]	
Level-3 (Certificate)	154(56.2)
Level-4 (Diploma)	120(43.8)
Work experience as a HEW in years	
≤5	106(38.7)
6-10	63(23.0)
>10	105(38.3)
iCCM training	
Yes	216(78.8)
No	58(21.2)
Received supportive supervision in the last 3 months	
Yes	197(71.9)
No	77(28.1)
Number of visits (in the last 3 months)	
No visit	77(28.1)
One to two	104(38.0)
Three and more	93(33.9)
Contents of supportive supervision[b]	
Discussing diagnosis and treatment of suspected pneumonia	132(67.0)
Discussing diagnosis and treatment of suspected diarrhea	139(70.6)
Observing record keeping	187(94.9)
Checking the register for consistency and completeness	183(92.9)
Checking supplies including manuals, job aids, and request forms	146(74.1)
Delivering supplies including manuals, job aids, and request forms	123(62.4)
Observing client consultation with providers	99(50.3)
Providing written feedback	131(66.5)
Participated in PRCMM in the last 6 months	
Yes	126(46.0)
No	148(54.0)
Region	
Amhara	130(47.4)
Oromia	71(25.9)
SNNP	37(13.5)
Tigray	36(13.1)

Abbreviations: iCCM, integrated community case management; PRCMM, performance review clinical mentoring meeting; SNNP, southern nations, nationalities, and people

[a]Level of Training: Health extension workers (HEW) level of training categories: Level-3 HEWs are those who graduated with a certificate after a one-year pre-service training on the 17 packages of the Health Extension Program. Level-4 HEWs are those who graduated with a diploma after one year of additional education at a technical college.

[b]Out of the total health extension workers that have received supervision in the last 3 months (N=197)

For peer review only

The HEWs have consulted a total of 685 sick children, with an average of 5 sick children consultations per HEW (standard deviation 1.6). The minimum and maximum consultations were 1 and 6, and the majority 127(84.7%) of the HEWs have consulted three and more sick children. Out of the total sick children who visited the HEWs 628(91.7%) were older than 6 months. Three hundred sixteen (46%) sick children had respiratory complaints and 302(44%) had diarrhea related complaints.

Medical equipment, job aids, and medicines availability

Medical equipment and job aids

A total of 121 (82.3%) providers had thermometers, 115(78.2%) had infant scales, 116(78.9%) had weighing slings, 79(53.7%) had tape measures, 69(46.9%) had blood pressure (BP) apparatus, 98(66.7%) had stethoscopes, 16(10.9%) had watch or clock, and 102(69.4%) had examination couch. Whereas all of the providers reported that they had mid-upper arm circumference (MUAC) tape, which is used to assess malnutrition.

Regarding job aids and tools, 145(98.6%) had iCCM register for 2-59 months, 97(66%) had stock/bin cards, 95(64.6%) had medicine request and resupply forms, and 138(93.9%) had chart booklets.

Medicines

Overall, 115 health extension workers (71.4%) out of 147 had access to amoxicillin on the date of assessment. Amoxicillin was out of stock for 24 health extension workers with an average stock-out day of 37 (SD 31.1). The minimum and maximum days of stock-out were 2 and 95 days, respectively.

For peer review only

For peer review only - http://bmjopen.bmj.com/site/about/guidelines.xhtml

Oral rehydration solution medicines were available to 137 (93.2%) providers and zinc to 125(85%) providers. Oral rehydration solution was out of stock for 6 providers with an average stock outage day of 22 (standard deviation 34.2). The minimum and maximum stock out days were 1 and 90 days, respectively. Whereas, zinc was out of stock for 19 providers with an average stock outage day of 38 (standard deviation 44.9). The minimum and maximum days for zinc were 1 and 180, respectively. Both ORS and zinc medicines were available to 117(79.6%) providers. Paracetamol tablets were available to 94 (63.9%) providers. Paracetamol was out of stock for 31 providers with an average stock outage day of 40 (standard deviation 31.6) with a range of 1-120 days (Table 2).

Table 2. Medical equipment, job aids, and medicine availability at the health posts of providers in four regions of Ethiopia, December 2018 to February 2019 (N=147)

Medical equipment, job aids, and Medicines	n(%)
Medical equipment	
Thermometer	121(82.3)
Infant scale	115(78.2)
Weighing sling	116(78.9)
Tape measures	79(53.7)
Blood pressure apparatus	69(46.9)
Stethoscopes	98(66.7)
Watch or clock (timer)	16(10.9)
Examination couch	102(69.4)
Mid-upper-arm circumference tape	147(100.0)
Basic equipment availability index[a]	44(29.9)
Job aids	
iCCM register (2-59 months old children)	145(98.6)
Stock/bin cards to track medicines	97(66.0)
Medicines request and resupply forms	95(64.6)
Chart booklets	138(93.9)
Medicines	
Amoxicillin	115(78.2)
Oral rehydration solution	137(93.2)
Zinc	125(85.0)
Oral rehydration solution and zinc	117(79.6)
Paracetamol	94(63.9)

For peer review only - http://bmjopen.bmj.com/site/about/guidelines.xhtml

Amoxicillin, oral rehydration solution and zinc	96(65.3)

320 [a] *Basic equipment availability index: Availability of all of the following for the same provider: thermometer, infant*
321 *scale, weighing sling, stethoscope, and BP apparatus*

322 Providers knowledge

323 Out of 274 providers who responded to the interview (Table 3), less than half of them had good
324 knowledge of pneumonia signs and symptoms (35.4%), diarrhea signs and symptoms (20.8%),
325 pneumonia management approaches (24.5%), and diarrhea management approaches (24.1%).
326 Overall, slightly over a quarter (27%) of the providers were knowledgeable about the signs and
327 symptoms, and management approaches of both illnesses. Mean pneumonia and diarrhea signs
328 and symptoms, and the management knowledge score of the HEWs was 48.31% (95% CI: 45.7,
329 50.9) with a standard deviation of 21.5.

330 Table 3. Providers knowledge of childhood pneumonia and diarrhea management in four regions
331 of Ethiopia, December 2018 to February 2019 (N=274)

Knowledge of-	n	%(95% CI)
Pneumonia signs and symptoms		
Good knowledge	97	35.4(29.9-41.2)
Poor knowledge	177	64.6(58.8-70.1)
Pneumonia management approaches		
Good knowledge	67	24.5(19.6-29.8)
Poor knowledge	207	75.5(70.2-80.4)
Pneumonia signs and symptoms and management approaches		
Good knowledge	108	39.4(33.7-45.3)
Poor knowledge	166	60.6(54.7-66.3)
Diarrhea signs and symptoms		
Good knowledge	57	20.8(16.3-25.9)
Poor knowledge	217	79.2(74.1-83.7)

For peer review only - http://bmjopen.bmj.com/site/about/guidelines.xhtml

Diarrhea management approaches		
Good knowledge	66	24.1(19.3-29.4)
Poor knowledge	208	75.9(70.6-80.7)
Diarrhea signs and symptoms and management approaches		
Good knowledge	78	28.5(23.4-34.0)
Poor knowledge	196	71.5(66.0-76.6)
Both illnesses sign and symptoms and management approaches		
Good knowledge	74	27.0(22.0-32.5)
Poor knowledge	200	73.0(67.5-78.0)

Know-do gap in child healthcare

There was a significant difference between the knowledge of the HEWs and their actual practices in the assessment of pneumonia and diarrhea ($p<0.05$ for individual and overall know-do gap proportions as in table 4). Slightly greater than one-third (35.7%) of HEWs had good knowledge of pneumonia signs and symptoms and only one-fifth of them (19.8%) had actually assessed those signs and symptoms. Though three-fourths of providers know cough or fast breathing as the sign and symptoms of pneumonia, only 47.3% of them have actually asked about cough or difficulty breathing. Likewise, 34.9% of providers have described chest-indrawing as a symptom of pneumonia while only 28.6% have assessed chest-indrawing in children.

Six out of ten (61.5%) of the providers have good knowledge of diarrhea signs and symptoms, however only four out of ten (40.2%) were able to actually assess those signs and symptoms in children who presented with diarrhea complaints.

Table 4. Providers know-do gap in the assessment of pneumonia and diarrhea in four regions of Ethiopia, December 2018 to February 2019

For peer review only - http://bmjopen.bmj.com/site/about/guidelines.xhtml

Assessment	Knowledge (n, %)	Practice (n, %)	Gap (%)	Z test p value
Pneumonia				
Cough or fast breathing	114(76.0)	71(47.3)	28.7	p<0.0001
Chest-indrawing[a]	44(34.9)	36(28.6)	6.3	0.20
Stridor[a]	35(27.8)	19(15.1)	12.7	0.006
Pneumonia index[a]	45(35.7)	25(19.8)	15.9	0.005
Diarrhea				
Presence of diarrhea	126(84.0)	62(41.3)	42.7	p<0.0001
Blood in the stool[b]	39(32.0)	52(42.6)	-10.6	0.17
Child restlessness or irritability[b]	39(32.0)	46(37.7)	-5.7	0.26
Pinch the skin of the abdomen[b]	56(45.9)	33(27.0)	18.9	0.001
Diarrhea index[b]	75(61.5)	49(40.2)	21.3	0.001
Overall index[c]	41(38.0)	23(21.3)	16.7	0.002

[a]*Out of the health extension workers that have consulted children who were labeled to have a cough or difficulty breathing (n=126)*
[b] *Out of the health extension workers that have consulted children who were labeled to have diarrhea (n=122)*
[c]*Out of the health extension workers that have consulted children who were labeled to have both cough and diarrhea (n=108)*

Similarly, a significant difference was observed between the knowledge and actual practice of providers regarding the treatment of childhood pneumonia and diarrhea ($p<0.05$). Most of the providers (96.2%) have reported that they knew treatment recommendations for pneumonia and diarrhea, but only a little more than a quarter of them (27.7%) have actually practiced the recommendations for children who were classified as having either pneumonia or diarrhea. On the contrary, a large proportion of providers practiced counseling about medications for children who were provided treatments for pneumonia and diarrhea though only a few of them know it as a component of management (Table 5).

For peer review only - http://bmjopen.bmj.com/site/about/guidelines.xhtml

361 Table 5. Providers know-do gap in the management of pneumonia and diarrhea in four regions of
362 Ethiopia, December 2018 to February 2019

Treatment and counseling	Knowledge (n, %)	Practice (n, %)	Gap (%)	Z test p value
Pneumonia				
Prescribe antibiotics[a]	68(88.3)	12(15.6)	72.7	p<0.0001
Counsel on medications[b]	2(16.7)	10(83.3)	(66.6)	0.001
Diarrhea				
Prescribe ORS and zinc[c]	108(93.9)	59(51.3)	42.6	p<0.0001
Counsel on medications[d]	18(30.5)	30(50.8)	(20.3)	0.03
Treatment for pneumonia and diarrhea[e]	126(96.2)	36(27.7)	68.5	p<0.0001

363 [a]*Out of the health extension workers who have consulted children classified as having pneumonia (N=77)*
364 [b]*Out of the health extension worker who has provided treatment to pneumonia (N=12)*
365 [c]*Out of the health extension workers who have consulted children classified as having diarrhea (N=115)*
366 [d]*Out of the health extension workers who have provided treatment to diarrhea (N=59)*
367 [e] *Out of the health extension workers who have consulted children who were classified as having either pneumonia*
368 *or diarrhea (N=130)*
369

370 Drivers of knowledge and actual practice of providers

371 Drivers of providers' knowledge

372 The majority of the providers who have service experience greater than 5 years (74.3%) as HEWs
373 and who received iCCM training (83.8%) had good knowledge of pneumonia and diarrhea signs
374 and symptoms and management approaches. Moreover, six out of ten of the providers who have
375 received supervision with components of discussing diagnosis and treatment of childhood illnesses
376 and those who participated in the performance review clinical mentoring meetings had good
377 knowledge.

378 In the bivariate analysis, providers' age, service experience, level of education, iCCM training,
379 supportive supervision, and participation on performance review clinical mentoring meetings were
380 identified as candidate variables for multiple variable logistic regression at p-value<0.2. In the
381 multiple variable logistic regression, only the participation in the performance review clinical

For peer review only - http://bmjopen.bmj.com/site/about/guidelines.xhtml

mentoring meetings showed statistically significant associations with the knowledge of the providers. Providers' knowledge of pneumonia and diarrhea management was nearly two times more likely among providers who were participated in the performance review clinical mentoring meetings that was held 6 months preceding the survey period (AOR=2.00, 95% CI: 1.09,3.67) (Table 6).

Table 6. Correlates of providers' knowledge of pneumonia and diarrhea management in four regions of Ethiopia, December 2018 to February 2019

Variables	Knowledge		AOR (95% CI)
	Good(n,%)	Poor(n,%)	
Observations	74	200	
Age in years			
≤30	43(58.1)	144(72.0)	Ref
>30	31(41.9)	56(28.0)	1.41(0.75-2.64)
Service experience as HEWs in years			
≤5	19(25.7)	87(43.5)	Ref
>5	55(74.3)	113(56.5)	1.78(0.81,3.92)
Level of HEWs			
Level-3 (Certificate)	34(45.9)	120(60.0)	Ref
Level-4 (Diploma)	40(54.1)	80(40.0)	1.33(0.74,2.39)
iCCM training			
No	12(16.2)	46(23.0)	Ref
Yes	62(83.8)	154(77.0)	0.71(0.29,1.71)
Supervision with discussion on diagnosis and treatment of pneumonia and diarrhea			
No	29(39.2)	103(51.5)	Ref
Yes	45(60.8)	97(48.5)	1.12(0.60,2.11)
Supervision with a component of observing client consultations			
No	38(51.3)	131(65.5)	Ref
Yes	36(48.7)	69(34.5)	1.36(0.73,2.53)
Being participated in PRCMM			
No	29(39.2)	119(59.5)	Ref
Yes	45(60.8)	81(40.5)	2.00(1.09,3.67)**

*** P<0.05 is considered statistically significant in the multivariate analysis*

Abbreviations: CI, confidence interval; iCCM, integrated community case management; PRCMM, performance review clinical mentoring meeting; HEW, health extension worker; AOR, adjusted odds ratio; Ref, reference category

For peer review only - http://bmjopen.bmj.com/site/about/guidelines.xhtml

For peer review only

Drivers of providers' actual practice

The majority of HEWs aged less than 30 years (77.8%) and those who have access to medicines for childhood conditions (83.3%) had better childhood pneumonia and diarrhea management practice. Moreover, greater than half of the HEWs who received supervision with a component of discussing the management of childhood conditions, and less than half of the providers who participated in the performance review clinical mentoring meetings had good actual management practice.

In the bivariate analysis, providers' age, service experience, level of education, iCCM training, supportive supervision, participation on performance review clinical mentoring meetings, knowledge of childhood pneumonia and diarrhea management, and availability of medicines were identified as candidate variables for multiple variable logistic regression at p-value<0.2. The multiple variable logistic regression analysis revealed that pneumonia and diarrhea medicines availability was associated with the actual management practices of providers. The rest of the variables didn't show associations. Correct management practice was four times more likely in providers who have access to medicines for the treatment of pneumonia and diarrhea compared to those who didn't have access (AOR=4.04, 95% CI: 1.48,11.06) (Table 7).

Table 7. Correlates of the providers' actual management practice of pneumonia and diarrhea in four regions of Ethiopia, December 2018 to February 2019

Variables	Practice		AOR (95% CI)
	Good(n,%)	Poor(n,%)	
Observations	36	94	
Age in years			
≤30	28(77.8)	70(74.5)	Ref
>30	8(22.2)	24(25.5)	0.61(0.22-1.75)
Service experience as HEWs in years			
≤5	14(38.9)	39(41.5)	Ref
>5	22(61.1)	55(58.5)	1.54(0.59-4.02)

For peer review only - http://bmjopen.bmj.com/site/about/guidelines.xhtml

Level of HEWs			
Level-3 (Certificate)	20(55.6)	59(62.8)	Ref
Level-4 (Diploma)	16(44.4)	35(37.2)	1.52(0.62-3.68)
iCCM training			
No	30(83.3)	75(79.8)	Ref
Yes	6(16.7)	19(20.2)	1.45(0.39-5.30)
Supervision with discussion on diagnosis and treatment of pneumonia and diarrhea			
No	15(41.7)	42(44.7)	Ref
Yes	21(58.6)	52(55.3)	1.13(0.44-2.87)
Supervision with a component of observing client consultations			
No	23(63.9)	58(61.7)	Ref
Yes	13(36.1)	36(38.3)	0.91(0.37-2.19)
Being participated in PRCMM			
No	19(52.8)	46(48.9)	Ref
Yes	17(47.2)	48(51.1)	0.60(0.24-1.50)
Knowledge index			
Poor knowledge	25(69.4)	67(71.3)	Ref
Good knowledge	11(30.6)	27(28.7)	1.04(0.41-2.64)
Amoxicillin, ORS and zinc availability[a]			
No	6(16.7)	38(40.4)	Ref
Yes	30(83.3)	53(59.6)	4.04(1.48-11.06)*

*** P<0.05 is considered statistically significant in the multivariate analysis*

Abbreviations: CI, confidence interval; iCCM, integrated community case management; PRCMM, performance review clinical mentoring meeting; HEW, health extension worker; AOR, adjusted odds ratio; Ref, reference category

[a]Out of providers who have assessed for medications availability in the health posts(n=127). Providers who had access to all the three medicines were regarded as 'yes' and otherwise 'no'.

Discussion

The study revealed that a low proportion of the providers had knowledge of the signs and symptoms and management of pneumonia and diarrhea. Similarly, a low proportion of the providers executed the recommended actions for the assessment and management of childhood pneumonia and dehydration. There was a significant difference between the knowledge and actual practice of the providers. This finding is consistent with a result among other health professionals in Ethiopia (14) and frontline health workers in India (13,17).

Slightly greater than one-third (35.7%) of the HEWs had good knowledge of pneumonia signs and symptoms and only one-fifth of them (19.8%) had actually assessed consulting children for those

For peer review only - http://bmjopen.bmj.com/site/about/guidelines.xhtml

For peer review only

signs and symptoms. The widest gap was observed between knowledge and actual practice of providers related to cough or difficulty breathing (76% vs. 47.3%) and stridor (27.8% vs. 15.1%). Besides, the incongruity between the knowledge and actual practices of the providers; the level of concordance with the clinical guidelines have remained low among the frontline providers. This has a huge impact on the clinical care outcomes related to childhood pneumonia. Poor adherence to clinical guidelines by providers in the assessment of sick child was also reported in earlier studies from low-and-middle-income countries (36,37) and in Ethiopia(28–30).

Though six out of ten (61.5%) of the providers had good knowledge of signs and symptoms of diarrhea, only four out of ten (40.2%) actually assessed for those signs and symptoms in children who presented with diarrhea complaints. A wide and significant gap was observed between the knowledge and actual practice of the providers related to overall diarrhea assessment (84% vs. 41.3%) and pinch skin of the abdomen (45.9% vs. 27%). On the contrary, a large proportion of providers have practiced assessing the presence of blood in the stool (42.6% vs. 32%), and child restlessness or irritability (37.7% vs. 32%) without reporting on knowledge of the symptoms at the outset. Though the clinical guidelines recommend assessment of all the signs and symptoms of dehydration, the providers didn't actually practice them (33). This could lead to incorrect diagnosis of sick children with possible dehydration and results in missed opportunity to provide life-saving treatment for those in need of it.

The know-do gap analysis also revealed that there was a significant difference between the knowledge and actual practice of providers. Most of the providers (96.2%) have reported that they know recommended treatment options for pneumonia and diarrhea, but only a quarter of them (27.7%) have actually provided treatment for children who were classified as having either pneumonia or diarrhea. Earlier studies from Ethiopia and Uganda also revealed that community

For peer review only - http://bmjopen.bmj.com/site/about/guidelines.xhtml

health workers had poor knowledge and practice of childhood illnesses management (38–40). On the contrary, a large proportion of providers practiced counseling about medications for children who were provided treatment for pneumonia and diarrhea though only a few of them knew it as a component of sick child care.

Many factors counter play the lack of knowledge and good practice related to the assessment and management of sick children. The quality of clinical care provided to sick children is a function of the providers' knowledge, the effort exerted by the provider to apply the knowledge, and the readiness of facilities to provide quality care in terms of medicines and supplies. In the current study, the providers' knowledge and actual practice of assessment and management of childhood pneumonia and diarrhea were associated with participation in performance review clinical mentoring meeting and the availability of medicines for pneumonia and dehydration treatment at the health posts. A previous study also revealed that clinical coaching is positively associated with good clinical practice among community health workers (41). The odds of good knowledge of pneumonia and diarrhea assessment and management was two times more likely in the providers who participated in the performance review clinical mentoring meeting. While, the odds of better correct practice were four times more likely in providers who had medicines for the treatment of childhood pneumonia and diarrhea (amoxicillin, ORS, and zinc). Other variables including the providers' characteristics (age, work or practice experience, level of training), receiving clinical training, and supportive supervision did not affect knowledge and actual practice of the HEWs. Though most of the providers were trained in iCCM, they scored low in the knowledge and practice for assessment and treatment of childhood pneumonia implying that in-serving trainings are necessary but not sufficient drivers of correct practice(42). In another study, variables including the providers' level of education, work or practice experience, the presence of supervision, and

For peer review only - http://bmjopen.bmj.com/site/about/guidelines.xhtml

guidelines were associated with the community health workers' knowledge of childhood illness management (39,40).

The findings of the present study have policy and practice implications. Clinical training interventions that lacked adequate practical skill component doesn't improve the competency of providers. The lack of refresher training is also a cause for poor performance due to the decay in knowledge. High-quality in-service training coupled with mentoring, supervisions, medicines, and material support are effective to improve the quality of care(42,44,45). Though training had high coverage in the study area, a significant number of providers are short of medicines, supplies, and technical support. Supervisions and mentoring focused on the management of childhood pneumonia and diarrhea were limited in the study area. The supervisors discussed the management of pneumonia and diarrhea in half of their visits (51.8%) and in greater than one-third (38.3%) they observed client consultations by the provider. While less than half (46%) of providers were clinically mentored according to the standard guideline. On the other hand, two-thirds (65.7%) of the providers had access to medicines for pneumonia and diarrhea. In-service training and supervision were not associated with knowledge, and actual practices of providers signaling a look at other approaches that improves quality of training and supervisions.

The study was conducted at the community health posts within the primary healthcare level, the lowest care level in the health system of Ethiopia. At this level the providers are supposed to diagnose and treat moderate cases, and refer severe cases to the health centers. This is the first study that examined the know-do gap in childhood pneumonia and diarrhea care among HEWs in Ethiopia. The know-do gap analysis was based on observations of actual management practices of the providers by experienced and trained health professionals, and interviews on the knowledge assessment components. Though our findings are limited by a small sample size, the study has

For peer review only

For peer review only - http://bmjopen.bmj.com/site/about/guidelines.xhtml

covered the four most populous regions of the country and is representative of the different settings in rural Ethiopia. On average the providers were observed while consulting five sick children, and this might ensure the heterogeneity of cases for both conditions. However, the clinical practices and actions of providers related to severe illnesses was not examined in the present research. The other limitation might be the Hawthorne effect i.e., a change in professional behavior of the HEWs when being observed (46). This might partly mask the actual practice of providers. But, the findings related to actual management practice in both conditions shown that the providers have performed less and in normal management situation this might be even worse.

Conclusions

The study revealed a huge know-do gap in the assessment and management of childhood pneumonia and diarrhea among the HEWs in Ethiopia. The finding affirm that knowledge-based training is necessary but not sufficient to influence the clinical performance (actual practice) of providers. The know-do gap of pneumonia and diarrhea management was determined by the presence of clinical mentoring and performance reviews and the availability of medicines. Therefore, an effort should be made to close the gap between theoretical knowledge and practice. The HEWs should be supported with mentoring, performance reviews, and the supply of medicines and commodities.

Acknowledgments

The authors acknowledge the administrative hierarchies and the research participants in the study area for their cooperation throughout the research process, and the time they devoted to the study in providing required information.

For peer review only

Authors' contributions

All authors made a significant contribution to the work reported, whether, that is, in the conception, study design, execution, acquisition of data, analysis, and interpretation, or in all these areas. DWD wrote the first draft of the manuscript, and MAW and MW revised the manuscript. All authors gave final approval of the version to be published, have agreed on the journal to which the article has been submitted, and agreed to be accountable for all aspects of the work. All authors read and approved the final manuscript.

Funding

This project was funded by Bill & Melinda Gates Foundation with a grant to London School of Hygiene & Tropical Medicine (INV-009691).

Disclaimer

The funder has no role in the study design, collection, management, analysis or interpretation of data.

Data availability

The datasets used and/or analyzed for the current study are not openly available because data are part of the ongoing research project and available from the corresponding author upon reasonable request (E-mail: dave86520@gmail.com or dawit.daka@ju.edu.et)

Competing interests

All authors declare that they have no competing interests.

Patient consent for publication

For peer review only - http://bmjopen.bmj.com/site/about/guidelines.xhtml

Not required

References

1. United Nations Children's Fund (UNICEF). Pneumonia and diarrhoea: Tackling the deadliest diseases for the world's poorest children. United Nations Children's Fund. 2012. 1–86 p. Available from: http://www.unicef.org/eapro/Pneumonia_and_Diarrhoea_Report_2012.pdf

2. United Nations Children's Fund (UNICEF). One is too many: Ending child deaths from pneumonia and diarrhoea. 2016. 25 p. Available from: https://data.unicef.org/wp-content/uploads/2016/11/UNICEF-Pneumonia-Diarrhoea-report2016-web-version_final.pdf

3. IVAC at Johns Hopkins Bloomberg School of Public Health. Pneumonia and Diarrhea Progress Report 2018. 2018. Available from: http://link.springer.com/10.1023/A:1020102612073%0Ahttp://apps.who.int/gb/ebwha/pdf_files/WHA66/A66_R10-en.pdf%0Ahttp://www.who.int/diabetes/country-profiles/nga_en.pdf?ua=1

4. International Vaccine Access Center (IVAC). Pneumonia and Diarrhea Progress Report 2020. John Hopkins Bloomberg School of Public Health. 2020. Available from: https://resourcecentre.savethechildren.net/pdf/ivac_pdpr_2020.pdf/

5. Nanyonjo A, Ssekitooleko J, Counihan H, Makumbi F, Tomson G, Källander K. Impact of an integrated community case management programme on uptake of appropriate diarrhoea and pneumonia treatments in Uganda: A propensity score matching and equity analysis study. Int J Equity Health. 2015;14:74. Available from: http://dx.doi.org/10.1186/s12939-

For peer review only - http://bmjopen.bmj.com/site/about/guidelines.xhtml

015-0202-y

6. Winskill P, Mousa A, Oresanya O, Counihan H, Okell LC, Walker PG. Does integrated community case management (iCCM) target health inequities and treatment delays? Evidence from an analysis of Demographic and Health Surveys data from 21 countries in the period 2010 to 2018. J Glob Health. 2021;11:040113.

7. Young M, Wolfheim C, Marsh DR, Hammamy D. World health organization/United Nations children's fund joint statement on integrated community case management: An equity-focused strategy to improve access to essential treatment services for children. Am J Trop Med Hyg. 2012;87(SUPPL.5):6–10.

8. World Health Organization. Integrated Management of Childhood Illness (IMCI): Chart Booklet. Geneva, Switzerland: World Health Organization. 2014.

9. Kallander K, Burgess DH, Qazi SA. Early identification and treatment of pneumonia: A call to action. Lancet Glob Heal. 2016;4(1):e12–3.

10. HEFDC Group. Integrated community case management (iCCM) in sub-Saharan Africa: Successes and challenges with access, speed and quality. Thematic Review Report. 2018.

11. Bagonza J, Kibira SPS, Rutebemberwa E. Performance of community health workers managing malaria , pneumonia and diarrhoea under the community case management programme in central Uganda : a cross sectional study. Malar J. 2014;13:367.

12. Chinbuah MA, Abbey M, Kager PA, Gyapong M, Nonvignon J, Ashitey P, et al. Assessment of the adherence of community health workers to dosing and referral guidelines for the management of fever in children under 5 years: a study in Dangme West

For peer review only

For peer review only - http://bmjopen.bmj.com/site/about/guidelines.xhtml

District, Ghana. Int Health. 2013;5:148–56.

13. Ray Saraswati L, Baker M, Mishra A, Bhandari P, Rai A, Mishra P, et al. ‘Know-Can’ gap: gap between knowledge and skills related to childhood diarrhoea and pneumonia among frontline workers in rural Uttar Pradesh, India. Trop Med Int Heal. 2020;25(4):454–66.

14. Gage AD, Kruk ME, Girma T, Lemango ET. The know-do gap in sick child care in Ethiopia. PLoS One. 2018;13(12):e0208898.

15. Tielsch JM. The Know, Do, and Quality Gaps in International Maternal and Child Health Care Interventions. JAMA Pediatr. 2015 Apr 1;169(4):313–4. Available from: https://doi.org/10.1001/jamapediatrics.2014.3741

16. Kebede D, Zielinski C, Mbondji PE, Sanou I, Asamoah-odei E, Soumbey-alley EW, et al. Improving the availability, quality and use of health information, research evidence and knowledge to strengthen health systems. World Heal Organ. 2010;(1 2):53–64.

17. Mohanan M, Vera-Hernández M, Das V et al. The Know-Do Gap in Quality of Health Care for Childhood Diarrhea and Pneumonia in Rural India. Jama Pediatr. 2015;169(4):349–57.

18. Mungai IG, Baghel SS, Soni S, Vagela S, Sharma M, Diwan V, et al. Identifying the know-do gap in evidence-based neonatal care practices among informal health care providers—a cross-sectional study from Ujjain, India. BMC Health Serv Res. 2020;20:966.

19. Wanduru P, Tetui M, Tuhebwe D, Ediau M, Okuga M, Nalwadda C, et al. The

For peer review only

For peer review only - http://bmjopen.bmj.com/site/about/guidelines.xhtml

performance of community health workers in the management of multiple childhood infectious diseases in Lira, northern Uganda – a mixed methods cross-sectional study. Glob Health Action. 2016 Dec 1;9:33194. Available from: https://doi.org/10.3402/gha.v9.33194

20. Rabbani F, Perveen S, Aftab W, Zahidie A, Sangrasi K, Qazi SA. Health workers' perspectives, knowledge and skills regarding community case management of childhood diarrhoea and pneumonia: A qualitative inquiry for an implementation research project "nigraan" in District Badin, Sindh, Pakistan. BMC Health Serv Res. 2016;16:462. Available from: http://dx.doi.org/10.1186/s12913-016-1699-5

21. Izudi J, Anyigu S, Ndungutse D. Adherence to Integrated Management of Childhood Illnesses Guideline in Treating South Sudanese Children with Cough or Difficulty in Breathing. Menahem S, editor. Int J Pediatr. 2017;2017:5173416. Available from: https://doi.org/10.1155/2017/5173416

22. Miller NP, Amouzou A, Tafesse M, Hazel E, Legesse H, Degefie T, et al. Integrated Community Case Management of Childhood Illness in Ethiopia : Implementation Strength and Quality of Care. Am J Trop Med Hyg. 2014;91(2):424–34.

23. Sinyangwe C, Graham K, Nicholas S, King R, Mukupa S, Källander K, et al. Assessing the quality of care for pneumonia in integrated community case management: A cross-sectional mixed methods study. PLoS One. 2016;11(3):e0152204.

24. Baynes C, Mboya D, Likasi S, Maganga D, Pemba S, Baraka J, et al. Quality of sick child-care delivered by community health workers in Tanzania. Int J Heal Policy Manag. 2018;7(12):1097–109. Available from: https://doi.org/10.15171/ijhpm.2018.63

For peer review only - http://bmjopen.bmj.com/site/about/guidelines.xhtml

25. Berhanu A, Alemayehu M, Daka K, Binu W, Suleiman M. Utilization of Integrated Community Case Management of Childhood Illnesses at Health Posts in Southern Ethiopia. Pediatr Heal Med Ther. 2020;Volume 11:459–67.

26. Assefa Y, Gelaw YA, Hill PS, Taye BW, Damme W Van. Community health extension program of Ethiopia , 2003 – 2018 : successes and challenges toward universal coverage for primary healthcare services. Global Health. 2019;15:24. Available from: https://globalizationandhealth.biomedcentral.com/track/pdf/10.1186/s12992-019-0470-1

27. Legesse H, Degefie T, Hiluf M, Sime K, Tesfaye C, Abebe H, et al. National scale-up of integrated community case management in rural Ethiopia: Implementation and early lessons learned. Ethiop Med J. 2014;52(December):15–26.

28. Daka DW, Wordofa MA, Woldie M, Persson Å, Berhanu D. Quality of clinical assessment and management of sick children by Health Extension Workers in four regions of Ethiopia : A cross-sectional survey. PLoS One. 2020;15(9):e0239361.

29. Beyene H, Hailu D, Tadele H, Persson LÅ, Berhanu D. Insufficient referral practices of sick children in Ethiopia shown in a cross-sectional survey. Acta Paediatr Int J Paediatr. 2020;109(9):1867–74.

30. Getachew T, Mekonnen S, Yitayal M, Persson LÅ, Berhanu D. Health Extension Workers' diagnostic accuracy for common childhood illnesses in four regions of Ethiopia: a cross-sectional study. Acta Paediatr Int J Paediatr. 2019;108(11):2100–6. Available from: https://onlinelibrary.wiley.com/doi/epdf/10.1111/apa.14888

31. Berhanu D, Okwaraji YB, Belayneh AB, Lemango ET, Agonafer N, Birhanu BG et al. Protocol for the evaluation of a complex intervention aiming at increased utilization of

For peer review only

For peer review only - http://bmjopen.bmj.com/site/about/guidelines.xhtml

primary child health services in Ethiopia: A before and after study in intervention and comparison areas. BMC Health Serv Res. 2010;20:339.

32. World Health Organization. Health facility survey : tool to evaluate the quality of care delivered to sick children attending outpatient facilities. Geneva PP - Geneva: World Health Organization; Available from: https://apps.who.int/iris/handle/10665/42643

33. Ministry of Health of Ethiopia. The Integrated Community Case Management (iCCM) Chart Booklet Ethiopia. 2014. Available from: http://lifesavingcommodities.org/wp-content/uploads/2014/08/iCCM-English-Chartbooklet-August-2013.pdf

34. Tesema AG, Peiris D, Abimbola S, Ajisegiri WS, Narasimhan P, Mulugeta A, et al. Community health extension workers' training and supervision in Ethiopia: Exploring impact and implementation challenges for non-communicable disease service delivery. PLOS Glob Public Heal. 2022 Nov 9;2(11):e0001160. Available from: https://doi.org/10.1371/journal.pgph.0001160

35. Assefa Y, Gelaw YA, Hill PS, Taye BW, Van Damme W. Community health extension program of Ethiopia, 2003–2018: successes and challenges toward universal coverage for primary healthcare services. Global Health. 2019;15(1):24. Available from: https://doi.org/10.1186/s12992-019-0470-1

36. Gilroy KE, Callaghan-koru JA, Cardemil C V, Nsona H, Amouzou A, Mtimuni A, et al. Quality of sick child care delivered by Health Surveillance Assistants in Malawi. Health Policy Plan. 2013;28:573–85.

37. Kruk RME, Gaga AD, Mbaruku GM, Leslie HH. Content of Care in 15,000 Sick Child Consultations in Nine Lower-Income Countries. Heal Serv Res. 2018;12842. Available

For peer review only - http://bmjopen.bmj.com/site/about/guidelines.xhtml

from: https://doi.org/10.1111/1475-6773.12842).

38. Bagonza J, Kibira SPS, Rutebemberwa E. Performance of community health workers managing malaria, pneumonia and diarrhoea under the community case management programme in central Uganda: A cross sectional study. Malar J. 2014;13(1):1–10.

39. Befekadu A, Yitayal M. Knowledge and practice of health extension workers on drug provision for childhood illness in west Gojjam, Amhara, Northwest Ethiopia. BMC Public Health. 2020;20:496. Available from: https://doi.org/10.1186/s12889-020-08602-y

40. Wanduru P, Tetui M, Tuhebwe D, Ediau M, Okuga M, Nalwadda C, et al. The performance of community health workers in the management of multiple childhood infectious diseases in Lira, northern Uganda - A mixed methods cross-sectional study. Glob Health Action. 2016;9(1).

41. Shiroya-Wandabwa M, Kabue M, Kasungami D, Wambua J, Otieno D, Waka C, et al. Coaching community health volunteers in integrated community case management improves the care of sick children under-5: Experience from Bondo, Kenya. Int J Integr Care. 2018;18(4):1–11.

42. Leslie HH, Gage A, Nsona H, Hirschhorn LR, Kruk ME. Training And Supervision Did Not Meaningfully Improve Quality Of Care For Pregnant Women Or Sick Children In Sub-Saharan Africa. Health Aff. 2016 Sep 1;35(9):1716–24. Available from: https://doi.org/10.1377/hlthaff.2016.0261

43. Leslie HH, Gage A, Nsona H, Hirschhorn LR, Kruk ME. Training and supervision did not meaningfully improve quality of care for pregnant women or sick children in sub-Saharan Africa. Health Aff. 2016;35(9):1716–24.

For peer review only

For peer review only - http://bmjopen.bmj.com/site/about/guidelines.xhtml

44. Bluestone J, Troncoso E, Fitzgerald L, Nagbe L, Tetteh G, Hellar A, et al. Three Pivots for Improving Health Care Provider Performance. Glob Heal Sci Pract. 2022 Feb 28;10(1):e2100625. Available from: http://www.ghspjournal.org/content/10/1/e2100625.abstract

45. Desta BF, Beshir IA, Tefera BB, Argaw MD, Demeke HZ, Kibret MA. Does frequency of supportive supervisory visits influence health service delivery?—Dose and response study. PLoS One. 2020 Jun 16;15(6):e0234819. Available from: https://doi.org/10.1371/journal.pone.0234819

46. Oswald D. Handling the Hawthorne effect: The challenges surrounding a participant observer. Rev Soc Stud. 2014 Oct 1;1:53–74.

For peer review only - http://bmjopen.bmj.com/site/about/guidelines.xhtml

STROBE Statement—Checklist of items that should be included in reports of ***cross-sectional studies***

	Item No	Recommendation	Page No
**Title and abstract**	1	(*a*) Indicate the study's design with a commonly used term in the title or the abstract	2
		(*b*) Provide in the abstract an informative and balanced summary of what was done and what was found	2&3
**Introduction**			
Background/rationale	2	Explain the scientific background and rationale for the investigation being reported	3-5
Objectives	3	State specific objectives, including any prespecified hypotheses	5
**Methods**			
Study design	4	Present key elements of study design early in the paper	5
Setting	5	Describe the setting, locations, and relevant dates, including periods of recruitment, exposure, follow-up, and data collection	5-6
Participants	6	(*a*) Give the eligibility criteria, and the sources and methods of selection of participants	6
Variables	7	Clearly define all outcomes, exposures, predictors, potential confounders, and effect modifiers. Give diagnostic criteria, if applicable	7-8
Data sources/ measurement	8*	For each variable of interest, give sources of data and details of methods of assessment (measurement). Describe comparability of assessment methods if there is more than one group	6-7
Bias	9	Describe any efforts to address potential sources of bias	7
Study size	10	Explain how the study size was arrived at	6
Quantitative variables	11	Explain how quantitative variables were handled in the analyses. If applicable, describe which groupings were chosen and why	9
Statistical methods	12	(*a*) Describe all statistical methods, including those used to control for confounding	9
		(*b*) Describe any methods used to examine subgroups and interactions	
		(*c*) Explain how missing data were addressed	
		(*d*) If applicable, describe analytical methods taking account of sampling strategy	
		(*e*) Describe any sensitivity analyses	
**Results**			
Participants	13*	(a) Report numbers of individuals at each stage of study—eg numbers potentially eligible, examined for eligibility, confirmed eligible, included in the study, completing follow-up, and analysed	10
		(b) Give reasons for non-participation at each stage	
		(c) Consider use of a flow diagram	
Descriptive data	14*	(a) Give characteristics of study participants (eg demographic, clinical, social) and information on exposures and potential confounders	10-11
		(b) Indicate number of participants with missing data for each variable of interest	
Outcome data	15*	Report numbers of outcome events or summary measures	13-16
Main results	16	(*a*) Give unadjusted estimates and, if applicable, confounder-adjusted estimates and their precision (eg, 95% confidence interval). Make clear which confounders were adjusted for and why they were included	16-18

For peer review only - http://bmjopen.bmj.com/site/about/guidelines.xhtml

		(*b*) Report category boundaries when continuous variables were categorized	
		(*c*) If relevant, consider translating estimates of relative risk into absolute risk for a meaningful time period	
Other analyses	17	Report other analyses done—eg analyses of subgroups and interactions, and sensitivity analyses	
**Discussion**			
Key results	18	Summarise key results with reference to study objectives	18
Limitations	19	Discuss limitations of the study, taking into account sources of potential bias or imprecision. Discuss both direction and magnitude of any potential bias	20-21
Interpretation	20	Give a cautious overall interpretation of results considering objectives, limitations, multiplicity of analyses, results from similar studies, and other relevant evidence	18-21
Generalisability	21	Discuss the generalisability (external validity) of the study results	18-21
**Other information**			
Funding	22	Give the source of funding and the role of the funders for the present study and, if applicable, for the original study on which the present article is based	22

*Give information separately for exposed and unexposed groups.

Note: An Explanation and Elaboration article discusses each checklist item and gives methodological background and published examples of transparent reporting. The STROBE checklist is best used in conjunction with this article (freely available on the Web sites of PLoS Medicine at http://www.plosmedicine.org/, Annals of Internal Medicine at http://www.annals.org/, and Epidemiology at http://www.epidem.com/). Information on the STROBE Initiative is available at www.strobe-statement.org.

For peer review only

For peer review only - http://bmjopen.bmj.com/site/about/guidelines.xhtml

BMJ Open

Know-do gap for sick child care and drivers of knowledge and practice among health extension workers in four regions of Ethiopia: a cross-sectional study

Journal:	*BMJ Open*
Manuscript ID	bmjopen-2022-069698.R2
Article Type:	Original research
Date Submitted by the Author:	07-Jul-2023
Complete List of Authors:	Daka, Dawit; Jimma University, Health Policy and Management<br>Wordofa, Muluemebet; Jimma University, Department of Population & Family Health<br>Woldie, Mirkuzie; Jimma University; Jimma University, Health Policy and Management
**Primary Subject Heading**:	Health services research
Secondary Subject Heading:	Health services research
Keywords:	Community child health < PAEDIATRICS, Quality in health care < HEALTH SERVICES ADMINISTRATION & MANAGEMENT, INFECTIOUS DISEASES, Public health < INFECTIOUS DISEASES

SCHOLARONE™
Manuscripts

I, the Submitting Author has the right to grant and does grant on behalf of all authors of the Work (as defined in the below author licence), an exclusive licence and/or a non-exclusive licence for contributions from authors who are: i) UK Crown employees; ii) where BMJ has agreed a CC-BY licence shall apply, and/or iii) in accordance with the terms applicable for US Federal Government officers or employees acting as part of their official duties; on a worldwide, perpetual, irrevocable, royalty-free basis to BMJ Publishing Group Ltd ("BMJ") its licensees and where the relevant Journal is co-owned by BMJ to the co-owners of the Journal, to publish the Work in this journal and any other BMJ products and to exploit all rights, as set out in our licence.

The Submitting Author accepts and understands that any supply made under these terms is made by BMJ to the Submitting Author unless you are acting as an employee on behalf of your employer or a postgraduate student of an affiliated institution which is paying any applicable article publishing charge ("APC") for Open Access articles. Where the Submitting Author wishes to make the Work available on an Open Access basis (and intends to pay the relevant APC), the terms of reuse of such Open Access shall be governed by a Creative Commons licence – details of these licences and which Creative Commons licence will apply to this Work are set out in our licence referred to above.

Other than as permitted in any relevant BMJ Author's Self Archiving Policies, I confirm this Work has not been accepted for publication elsewhere, is not being considered for publication elsewhere and does not duplicate material already published. I confirm all authors consent to publication of this Work and authorise the granting of this licence.

For peer review only

Know-do gap for sick child care and drivers of knowledge and practice among health extension workers in four regions of Ethiopia: a cross-sectional study

Dawit Wolde Daka[1*], Muluemebet Abera Wordofa[2], and Mirkuzie Woldie[1,3]

[1]Faculty of Public Health, Department of Health Policy and Management, Jimma University, Jimma, Ethiopia

[2]Faculty of Public Health, Department of Population and Family Health, Jimma University, Jimma, Ethiopia

[3] Fenot Project, University of British Columbia, School of Public Health and Population, Addis Ababa, Ethiopia

*Corresponding Author

E-mail: dawit.daka86520@gmail.com or dave86520@gmail.com

https://orcid.org/0000-0001-5465-6345

For peer review only - http://bmjopen.bmj.com/site/about/guidelines.xhtml

For peer review only

Abstract

Objective: Though efforts were made to expand community-based sick child healthcare in Ethiopia, the quality of care provided remained low. Improving quality of care requires understanding providers' knowledge of appropriate care and their actual execution of tasks. This study examined gap between what health extension workers(HEWs) knew and did during the management of sick children in Ethiopia.

Design: Facility-based cross-sectional study was conducted.

Setting: The study was carried out in 52 districts across four regions in Ethiopia.

Participants: We interviewed 274 HEWs and performed observations of consultations done by 150 HEWs supplemented with facility assessment from December 2018 to February 2019.

Methodology: We compared providers' knowledge and performance in the management of childhood pneumonia and diarrhea. Know-do gap implies the difference in proportion between knowledge and actual practice of HEWs. Logistic regression was used to identify predictors of knowledge and actual practice.

Results: Providers' correct knowledge ranged from 27.8% to 76.0% for signs and symptoms of pneumonia, and 32.0% to 84% for dehydration signs. Their actual practices ranged from 15.1% to 47.3% for pneumonia, and 27.0% to 42.6% for dehydration. The correct knowledge and actual practices for pneumonia and dehydration management were 88.3% vs 15.6% and 93.9% vs 51.3%, respectively. There was significant know-do gap in assessments (16.7%, p=0.002) and management of childhood conditions (68.5%, p<0.0001). Mentorships were associated with providers' knowledge of clinical management, while medicines availability was associated with their actual management practice.

For peer review only - http://bmjopen.bmj.com/site/about/guidelines.xhtml

Conclusions: While knowledge and actual practice for assessment and management of pneumonia and dehydration ranged from very low to high, what is more concerning is the huge know-do gap among HEWs. Our findings suggest that knowledge-based training is necessary but not sufficient for ensuring correct assessment and management of sick children by HEWs. Continuous support through mentorships and the supply of commodities are critically needed.

Keywords: Knowledge, actual practice, know-do gap, pneumonia, diarrhea, assessment, management, health extension workers, Ethiopia

Strength and limitations

- This is the first study that examined the know-do gap of sick childcare among health extension workers in Ethiopia
- The questionnaires were adapted from validated tools and observations of sick child consultations were performed by clinicians who had received refresher training on integrated community case management (iCCM)
- Though the findings are limited by sample size, the study has covered the four largest regions of the country and is representative of the different settings in rural Ethiopia.
- Moreover, the observation method could be influenced by the Hawthorne effect through masking the actual practice due to a change in the professional behavior of the health extension workers when being observed. However, the findings revealed that the providers have performed less, and this might even be worse in normal situations.

For peer review only - http://bmjopen.bmj.com/site/about/guidelines.xhtml

Introduction

Pneumonia and diarrhea remain the two major childhood illnesses that contributed to the larger portion of under-five mortality worldwide (1,2). Globally, over 1.23 million children died of pneumonia and diarrhea before reaching their fifth birthday in 2018. Seventy percent of all pneumonia and diarrhea deaths in children under the age of five years have occurred in 15 countries of the world alone, and Ethiopia is one among them. In Ethiopia, 44, 692 under-five children's deaths were attributed to pneumonia and diarrhea in 2017 which accounted for 13.6 deaths per 1000 live births. Out of sick children with suspected pneumonia, only 31% were taken to an appropriate care provider and 7% received the recommended antibiotics. Similarly, of those under five children with diarrhea, 30% have received oral rehydration solution (ORS) and 33% were provided with zinc supplements (3,4).

The integrated community case management (iCCM) approach to common childhood illnesses is a key strategy for improving the treatment of potentially life-threatening illnesses in children (5–7). The iCCM guideline recommends that all children 2-59 months of age are first assessed for general danger signs, then for cough or difficult breathing, diarrhea, fever, ear problem, acute malnutrition, and anemia. Then children are classified based on assessment results and treated according to the standard of care(8). Adherence to guidelines by front-line providers is critical to improving the quality of care for sick children and improving treatment outcomes (9). However, studies have shown that providers' adherence to the clinical standards of sick children assessment and management is low in most low- and middle-income settings (10–12) and a significant know-do gap in clinical practices was common among the health care providers(13,14).

For peer review only - http://bmjopen.bmj.com/site/about/guidelines.xhtml

The know-do gap, failure to apply existing knowledge to improve health outcome, has a huge impact on the effort made towards reducing under-five mortalities attributed to poor quality or substandard care. It results in inconsistencies in care and inefficiencies in workflows. Though much is known about the evidence-based interventions related to maternal and child healthcare globally, it is not practiced as intended and the practices vary across the different settings (15). Most of the know-do gap is a result of inadequately skilled providers, lack of key equipment and medicines, and financial resources(14,16,17). Other factors impacting on the likelihood of providers practicing what they know include provider background characteristics (sex, age, working experience, level of education), extent of community support, and presence support in the form of regular trainings, mentoring, and supportive supervision(11,13,18–20). On the other hand, if community health workers are supported adequately, they can assess and treat sick children according to the protocol in the majority of the cases (21–23).

Narrowing the know-do gap is critical to attaining child survival goals and the targets set under the Global Action Plan for the Prevention and Control of Pneumonia and Diarrhea (GAPPD) in 2025. One of the core interventions outlined in the action plan was the appropriate management of sick children who are at risk of serious illnesses or death due to these two diseases (pneumonia and diarrhea). It is aimed to increase treatment coverage for children with suspected pneumonia including care by an appropriate healthcare provider and antibiotics, and treatment coverage for children with diarrhea including treatments with ORS and zinc supplements to 90% (4).

In Ethiopia, the integrated community case management services implementation was started in 2010 as part of the health extension program(21,24). Currently, the program is implemented in more than 18,000 health posts nationally(25). Despite huge investments and high coverage of clinical training (21,26), studies revealed that the quality of care is low in the health posts of

For peer review only - http://bmjopen.bmj.com/site/about/guidelines.xhtml

Ethiopia in terms of sick children assessment, classification, treatment, and referral practices(27–29). On the other hand, little is known about the know-do gap in sick child assessment and management, and the drivers among health extension workers(HEWs) in Ethiopia. Therefore, this paper aimed to examine the level of agreement between the knowledge the HEWs had about assessment and management and their actual practice of childhood pneumonia and diarrhea.

Methods

Study setting and period

The study was conducted in the health posts of 52 districts across the four most populous regions of Ethiopia: Oromia, Amhara, Southern Nations Nationalities and People's (SNNP), and Tigray from December 2018 to February 2019. The four regions and the districts were selected by the Ministry of Health, Regional Health Bureaus, and the implementing partners in 2016 for the planned intervention. The intervention districts were selected for having poor primary health service utilization indicators for under five children and the comparison districts were identified based on their similarity in population size and key maternal and child health indicators. This paper was part of the larger Dagu end-line survey, a survey that aimed to estimate the effectiveness of the optimizing health extension program (OHEP) in Ethiopia and the baseline survey was conducted from December 2016 to February 2017. The protocol for the OHEP evaluation was published elsewhere (30).

Study design and participants

A facility-based cross-sectional study was conducted among health extension workers that fulfilled the inclusion criteria. Two-stage stratified sampling strategy was used to select the study participants from the districts. In the first stage enumeration areas (EAs) were selected from the

For peer review only - http://bmjopen.bmj.com/site/about/guidelines.xhtml

study districts using the list of EAs from the 2007 Ethiopian Housing and Population Census as the sampling frame. The cumulative population size to clusters across the study areas was calculated and 200 EAs were selected with a probability proportional to population size (PPS). Each EA formed a cluster and each cluster constituted a primary sampling unit. In the second stage, all health posts serving the catchment population of the selected EAs were included in the study. Similarly, all HEWs who were present during the survey period were included in the study. Moreover, the sick children who visited the selected health posts to seek care from HEWs at the time of data collection were included.

Measurement

Structured interviews, observation, and health post-assessment questionnaires were used to collect data. The questionnaires were developed based on the World Health Organization (WHO) tool for Integrated Management of Childhood Illnesses (IMCI) sick child quality assessment in primary healthcare facilities(31) and the national iCCM guidelines(32). The health provider's interview questions included items such as background characteristics, iCCM training, supportive supervision, clinical mentorships, and knowledge of assessment and management of sick children 2-59 months. The observation questionnaire captured data on the reason for visits, assessment, classification, treatment, referral, and counseling. The facility assessment tool had sections of health posts infrastructure, medical equipment, medicines, and job aids. All the questionnaires were uploaded on tablets (CSPro 7.1) for data collection.

Data collectors with a qualification of bachelor's degree in health science were recruited and involved in data collection. Likewise, supervisors with a health background and qualifications with a bachelor of science and above have participated. Data collectors and supervisors were trained for 10 days on the questionnaires, data collection techniques, how to use tablets, and research

For peer review only - http://bmjopen.bmj.com/site/about/guidelines.xhtml

ethics. Clinical observations of providers were conducted following interviews and facility assessments. The supervisors have monitored the daily data collection process at the field level and provided support. Moreover, a data manager at the central office monitored data on the server and provided feedback to the field team.

Study variables

The outcome variable was the know-do gap of child care related to pneumonia and diarrhea assessment and management. The providers' knowledge data were obtained from interviews, and the actual practice data were collected through observation by clinicians. Know-do gap was the difference between the knowledge of HEWs about the signs and symptoms and management of pneumonia and diarrhea and their ability to correctly assess those signs and symptoms when children are presented with the illnesses. A provider was regarded as having a know-do gap in the following conditions: (a) if correctly named the signs and symptoms of the illnesses but failed to actually identify them; (b) if unable to correctly named the signs and symptoms of the illnesses but able to identify them, and (c) if unable to correctly named the signs and symptoms of the illnesses as well as unable to correctly identify them. On the other hand, if the provider correctly named the signs and symptoms, and also correctly identified them, then he/she was regarded as with no know-do gap. Similarly, the above conditions were applied to the know-do gap of pneumonia and dehydration management by the providers.

A total of 11 knowledge items were used, each having 'yes=1' and 'no=0' responses. Providers were asked to respond to questions without prompts and the responses were recorded from the pre-defined list. Then the interviewer recorded the responses of the providers. The knowledge questions were: what are the main signs and symptoms of pneumonia in children 2-59 months (3 responses)? When a child presents with signs and symptoms of pneumonia, what initial measures

For peer review only - http://bmjopen.bmj.com/site/about/guidelines.xhtml

do you take (2 responses)? What are the main signs and symptoms of diarrhea in children 2-59 months (4 responses)? When a child presents with signs and symptoms of diarrhea, what initial measures do you take (2 responses)?. The initial measures taken by providers for pneumonia have included prescribing antibiotics and counseling on medications, and for diarrhea, the actions were providing ORS and zinc, and counseling on medications.

We have constructed the knowledge index based on the Ethiopian university students' grading system with a few modifications as there was no validated standard to evaluate providers' knowledge. The Ethiopian university grading system is excellent [85-100%], very good [75 to 85%), good [60-75%), satisfactory [50-60%), unsatisfactory [45,50%), and very poor (below 45%). The providers who have scored 60% and above were categorized as having 'Good knowledge/knowledgeable=1', and otherwise 'poor knowledge/not knowledgeable=0'.

Hence, the HEWs were labeled knowledgeable if they have correctly responded to ≥60% of the knowledge items (3 and more items out of 5 for pneumonia; 4 and more items out of 6 for diarrhea; 7 and more items out of 11 for average knowledge). All scores below 60% were regarded as poor knowledge for the individual and average scores.

The ability to correctly assess and manage children (the actual practice of providers) for pneumonia and diarrhea index was constructed. The providers who correctly assessed children that presented with respiratory and diarrhea complaints according to the iCCM guidelines were categorized as 'correct practice for assessment=1' and otherwise 'incorrect practice for assessment=0'. Likewise, the providers who correctly treated children who have been classified as having pneumonia or diarrhea at the outset according to the iCCM guidelines were categorized as 'correct practice for treatment=1' and otherwise 'incorrect practice for treatment=0'. The providers were observed while consulting more than one sick children and hence, correct assessment and treatment for both

For peer review only

For peer review only - http://bmjopen.bmj.com/site/about/guidelines.xhtml

conditions were defined as being able to correctly assess and treat all children according to the iCCM guideline.

The tasks of pneumonia assessment according to the iCCM guidelines were ‘ask for the presence of cough or difficult breathing, check chest-indrawing, and listen for stridor’. The tasks for diarrhea assessment were ‘ask for the presence of diarrhea, ask the presence of blood in the stool, check child restlessness or irritability, and check whether skin pinch goes back slowly or very slowly’. The task of pneumonia treatment was providing antibiotics to sick children classified as having pneumonia, and the task of diarrhea treatment was providing ORS and zinc to those sick children who were classified as having diarrhea. The providers that correctly assessed children for pneumonia and diarrhea were categorized as ‘correct practice for assessment=1’ and otherwise ‘incorrect practice for assessment=0’. Those providers who treated all children who were classified as having pneumonia, and diarrhea with antibiotics, and ORS and Zinc were labeled as ‘correct practice for treatment=1’ and the providers who didn’t provide recommended treatments in either of the conditions were labeled as ‘incorrect practice for treatment=0’.

The explanatory variables of knowledge and actual practices were provider characteristics (age, working experience as HEWs, training level of HEWs (level-3 and level-4)), iCCM training status, presence of supportive supervision and mentorships, and presence of medicines and supplies. Level-3 HEWs are those who graduated with a certificate after a one-year pre-service training on the 17 packages of the health extension program, and level-4 HEWs are those who have level-3 certificate and got one additional year training at a technical college(25,33). The 17 packages of the health extension program are categorized into four components or areas as (a) Family health services (maternal and child health, family planning, immunization, adolescent reproductive health, nutrition); (b) Disease prevention and control (HIV/AIDS, Tuberculosis, malaria, first aid);

For peer review only

For peer review only - http://bmjopen.bmj.com/site/about/guidelines.xhtml

(c) Hygiene and environmental sanitation (proper and safe excreta disposal, proper and safe solid and liquid waste management, water supply safety measures, food hygiene and safety measures, healthy home environment, arthropods and rodents control, personal hygiene); and (d) Health education and communication(25).

Data processing and analysis

Primarily, the HEWs module data set and the observation module data set were linked using individual ID and then linked to the health facility assessment data set. The health facility assessment data included information on health post infrastructure, availability of medical equipment, medicines, and job aids. Data values were cleaned for inconsistency and incompleteness. Coding and re-coding of variables were done, and the indices were computed as appropriate. The know-do gap analysis was conducted for those HEWs who have been observed while consulting sick children, and analysis for knowledge about sick child management was conducted for all the HEWs included in the study. Further details on the approach and outcome of this analysis is provided in the results section.

Descriptive statistics were performed to analyze and describe the characteristics of providers, other explanatory variables, and the outcome variable. A 95% confidence interval was constructed for the outcome variable and some of the explanatory variables. To assess whether there exist significant differences between the proportion of providers with the correct knowledge and correct practice, we have conducted two-sample z-tests for differences in proportions. Bivariate logistic regression was used to select candidate explanatory variables for multiple variable logistic regression at $p\text{-value}<0.2$ and multiple variable logistic regression was used to analyze the association between explanatory variables with knowledge, and actual management practice of the providers. The variables considered in this analysis were provider age, years of experience, level

For peer review only - http://bmjopen.bmj.com/site/about/guidelines.xhtml

of education (level-3 and level-4), iCCM training status, presence of supportive supervision and mentorships, and presence of medicines and supplies. Those variables with a p-value<0.05 were considered statistically significant. All analysis was performed using SPSS version 20 (IBM corporation, New York, USA).

Ethical consideration

Research ethical approval was obtained from the Ethiopian Public Health Institute (protocol number SERO-012-8-2016; 001 August 2016), the London School of Hygiene and Tropical Medicine (protocol number 11235, June 2016), and Jimma University (protocol number IHRPGD/472/2018, August 2018). A support letter was secured from the regional health bureaus and zonal health offices. Informed written consent was taken from providers and for observation, consent was also taken from caregivers. For caregivers who were unable to read and write, witness was presented and signatures appended on the consent form. The confidentiality of providers and patient information was maintained properly through the use of codes and computer-based data were secured with passwords. Data will be used for the intended research purpose and will not be shared with a third party.

Patient and public involvement

Patient and/or the public were not involved in the design or conduct, or reporting or dissemination plans of this research.

Results

Description of background characteristics

From the total of 274 HEWs interviewed, 150 examined sick children and were included in the know-do gap and actual practice analysis. While 274 HEWs were used in the knowledge analysis.

For peer review only - http://bmjopen.bmj.com/site/about/guidelines.xhtml

The majority (78.8%) of providers had received iCCM training and out of which the larger proportion had received the training within the 12 months preceding the date of the survey (187 or 86.6%). Though the majority of the HEWs had received supervision within the 3 months preceding the survey date (71.9%), only 33.9% had been visited regularly. Furthermore, less than half (46%) of the HEWs participated in the performance review clinical mentoring meetings (PRCMM) that were held 6 months preceding the survey date. The details for the background characteristics of the HEWs included in the study is provided in Table 1.

Table 1. Background characteristics of the health extension workers in four regions of Ethiopia, December 2018 to February 2019

Variables	n(%)
Age in years	
≤30	187(68.2)
>30	87(31.8)
Level of Training[a]	
Level-3 (Certificate)	154(56.2)
Level-4 (Diploma)	120(43.8)
Work experience as a HEW in years	
≤5	106(38.7)
6-10	63(23.0)
>10	105(38.3)
iCCM training	
Yes	216(78.8)
No	58(21.2)
Received supportive supervision in the last 3 months	
Yes	197(71.9)
No	77(28.1)
Number of visits (in the last 3 months)	
No visit	77(28.1)
One to two	104(38.0)
Three and more	93(33.9)
Contents of supportive supervision[b]	
Discussing diagnosis and treatment of suspected pneumonia	132(67.0)

For peer review only - http://bmjopen.bmj.com/site/about/guidelines.xhtml

Discussing diagnosis and treatment of suspected diarrhea	139(70.6)
Observing record keeping	187(94.9)
Checking the register for consistency and completeness	183(92.9)
Checking supplies including manuals, job aids, and request forms	146(74.1)
Delivering supplies including manuals, job aids, and request forms	123(62.4)
Observing client consultation with providers	99(50.3)
Providing written feedback	131(66.5)
Participated in PRCMM in the last 6 months	
Yes	126(46.0)
No	148(54.0)
Region	
Amhara	130(47.4)
Oromia	71(25.9)
SNNP	37(13.5)
Tigray	36(13.1)

Abbreviations: iCCM, integrated community case management; PRCMM, performance review clinical mentoring meeting; SNNP, southern nations, nationalities, and people
[a]Level of Training: Health extension workers (HEW) level of training categories: Level-3 HEWs are those who graduated with a certificate after a one-year pre-service training on the 17 packages of the Health Extension Program. Level-4 HEWs are those who graduated with a diploma after one year of additional education at a technical college.
[b]Out of the total health extension workers that have received supervision in the last 3 months (N=197)

The HEWs have consulted a total of 685 sick children, with an average of 5 sick children consultations per HEW (standard deviation 1.6). The minimum and maximum consultations were 1 and 6 respectively, and the majority, 127(84.7%), of the HEWs have consulted three or more sick children. Out of the total sick children who visited the HEWs, 628(91.7%) were older than 6 months. Three hundred sixteen (46%) sick children had respiratory complaints and 302(44%) had diarrhea related complaints.

Medical equipment, job aids, and medicines availability

A total of 121 (82.3%) providers had thermometers, 115(78.2%) had infant scales, 116(78.9%) had weighing slings, 79(53.7%) had tape measures, 69(46.9%) had blood pressure (BP) apparatus, 98(66.7%) had stethoscopes, 16(10.9%) had watch or clock, and 102(69.4%) had examination couch. Whereas all of the providers reported that they had mid-upper arm circumference (MUAC)

For peer review only - http://bmjopen.bmj.com/site/about/guidelines.xhtml

tape, which is used to assess malnutrition. Regarding job aids and tools, 145(98.6%) had iCCM register for 2-59 months old children, and 138(93.9%) had chart booklets.

Overall, 115 HEWs (71.4%) out of 147 had access to amoxicillin on the date of assessment. Oral rehydration solution medicines were available to 137 (93.2%) providers and zinc to 125(85%) providers. Both ORS and zinc medicines were available to 117(79.6%) providers (S1 Table).

Providers knowledge

Out of 274 providers who responded to the interview (S2 Table), less than half of them had good knowledge of pneumonia signs and symptoms (35.4%), diarrhea signs and symptoms (20.8%), pneumonia management approaches (24.5%), and diarrhea management approaches (24.1%). Overall, slightly over a quarter (27%) of the providers were knowledgeable about the signs and symptoms, and management approaches of both illnesses. Mean pneumonia and diarrhea signs and symptoms, and the management knowledge score of the HEWs was 48.31% (95% CI: 45.7, 50.9) with a standard deviation of 21.5.

Know-do gap in child healthcare

There was a significant difference between the knowledge of the HEWs and their actual practices in the assessment of pneumonia and diarrhea ($p<0.05$ for individual and overall know-do gap proportions as in table 2). Slightly greater than one-third (35.7%) of HEWs had good knowledge of pneumonia signs and symptoms and only one-fifth of them (19.8%) had actually assessed those signs and symptoms. Though three-fourths of providers know cough or fast breathing as the sign and symptoms of pneumonia, only 47.3% of them have actually asked about cough or difficulty breathing. Likewise, 34.9% of providers have described chest-indrawing as a symptom of pneumonia while only 28.6% have assessed chest-indrawing in children.

For peer review only - http://bmjopen.bmj.com/site/about/guidelines.xhtml

Six out of ten (61.5%) of the providers have good knowledge of diarrhea signs and symptoms, however only four out of ten (40.2%) were able to actually assess those signs and symptoms in children who presented with diarrhea complaints.

Table 2. Providers know-do gap in the assessment of pneumonia and diarrhea in four regions of Ethiopia, December 2018 to February 2019

Assessment	Knowledge (n, %)	Practice (n, %)	Gap (%)	Z test p value
Pneumonia				
Cough or fast breathing	114(76.0)	71(47.3)	28.7	p<0.0001
Chest-indrawing[a]	44(34.9)	36(28.6)	6.3	0.20
Stridor[a]	35(27.8)	19(15.1)	12.7	0.006
Pneumonia index[a]	45(35.7)	25(19.8)	15.9	0.005
Diarrhea				
Presence of diarrhea	126(84.0)	62(41.3)	42.7	p<0.0001
Blood in the stool[b]	39(32.0)	52(42.6)	-10.6	0.17
Child restlessness or irritability[b]	39(32.0)	46(37.7)	-5.7	0.26
Pinch the skin of the abdomen[b]	56(45.9)	33(27.0)	18.9	0.001
Diarrhea index[b]	75(61.5)	49(40.2)	21.3	0.001
Overall index[c]	41(38.0)	23(21.3)	16.7	0.002

[a]Out of the health extension workers that have consulted children who were labeled to have a cough or difficulty breathing (n=126)
[b] Out of the health extension workers that have consulted children who were labeled to have diarrhea (n=122)
[c]Out of the health extension workers that have consulted children who were labeled to have both cough and diarrhea (n=108)

Similarly, a significant difference was observed between the knowledge and actual practice of providers regarding the treatment of childhood pneumonia and diarrhea (p<0.05). Most of the providers (96.2%) have reported that they knew treatment recommendations for pneumonia and

For peer review only

For peer review only - http://bmjopen.bmj.com/site/about/guidelines.xhtml

diarrhea, but only a little more than a quarter of them (27.7%) have actually practiced the recommendations for children who were classified as having either pneumonia or diarrhea. On the contrary, a large proportion of providers practiced counseling about medications for children who were provided treatments for pneumonia and diarrhea though only a few of them know it as a component of management (Table 3).

Table 3. Providers know-do gap in the management of pneumonia and diarrhea in four regions of Ethiopia, December 2018 to February 2019

Treatment and counseling	Knowledge (n, %)	Practice (n, %)	Gap (%)	Z test p value
Pneumonia				
Prescribe antibiotics[a]	68(88.3)	12(15.6)	72.7	p<0.0001
Counsel on medications[b]	2(16.7)	10(83.3)	(66.6)	0.001
Diarrhea				
Prescribe ORS and zinc[c]	108(93.9)	59(51.3)	42.6	p<0.0001
Counsel on medications[d]	18(30.5)	30(50.8)	(20.3)	0.03
Treatment for pneumonia and diarrhea[e]	126(96.2)	36(27.7)	68.5	p<0.0001

[a]*Out of the health extension workers who have consulted children classified as having pneumonia (N=77)*
[b]*Out of the health extension worker who has provided treatment to pneumonia (N=12)*
[c]*Out of the health extension workers who have consulted children classified as having diarrhea (N=115)*
[d]*Out of the health extension workers who have provided treatment to diarrhea (N=59)*
[e] *Out of the health extension workers who have consulted children who were classified as having either pneumonia or diarrhea (N=130)*

Drivers of knowledge and actual practice of providers

Drivers of providers' knowledge

The majority of the providers who have service experience greater than 5 years (74.3%) as HEWs and who received iCCM training (83.8%) had good knowledge of pneumonia and diarrhea signs and symptoms and management approaches. Moreover, six out of ten of the providers who have received supervision with components of discussing diagnosis and treatment of childhood illnesses and those who participated in the performance review clinical mentoring meetings had good knowledge.

For peer review only - http://bmjopen.bmj.com/site/about/guidelines.xhtml

For peer review only

In the bivariate analysis, providers' age, service experience, level of education, iCCM training, supportive supervision, and participation on performance review clinical mentoring meetings were identified as candidate variables for multiple variable logistic regression at p-value<0.2. In the multiple variable logistic regression, only the participation in the performance review clinical mentoring meetings showed statistically significant associations with the knowledge of the providers. Providers' knowledge of pneumonia and diarrhea management was nearly two times more likely among providers who were participated in the performance review clinical mentoring meetings that was held 6 months preceding the survey period (AOR=2.00, 95% CI: 1.09,3.67) (Table 4).

Table 4. Correlates of providers' knowledge of pneumonia and diarrhea management in four regions of Ethiopia, December 2018 to February 2019

Variables	Knowledge		AOR (95% CI)
	Good(n,%)	Poor(n,%)	
Observations	74	200	
Age in years			
≤30	43(58.1)	144(72.0)	Ref
>30	31(41.9)	56(28.0)	1.41(0.75-2.64)
Service experience as HEWs in years			
≤5	19(25.7)	87(43.5)	Ref
>5	55(74.3)	113(56.5)	1.78(0.81,3.92)
Level of HEWs			
Level-3 (Certificate)	34(45.9)	120(60.0)	Ref
Level-4 (Diploma)	40(54.1)	80(40.0)	1.33(0.74,2.39)
iCCM training			
No	12(16.2)	46(23.0)	Ref
Yes	62(83.8)	154(77.0)	0.71(0.29,1.71)
Supervision with discussion on diagnosis and treatment of pneumonia and diarrhea			
No	29(39.2)	103(51.5)	Ref
Yes	45(60.8)	97(48.5)	1.12(0.60,2.11)
Supervision with a component of observing client consultations			
No	38(51.3)	131(65.5)	Ref
Yes	36(48.7)	69(34.5)	1.36(0.73,2.53)
Being participated in PRCMM			
No	29(39.2)	119(59.5)	Ref

For peer review only - http://bmjopen.bmj.com/site/about/guidelines.xhtml

Yes	45(60.8)	81(40.5)	2.00(1.09,3.67)**

*** $P<0.05$ is considered statistically significant in the multivariate analysis*

Abbreviations: CI, confidence interval; iCCM, integrated community case management; PRCMM, performance review clinical mentoring meeting; HEW, health extension worker; AOR, adjusted odds ratio; Ref, reference category

Drivers of providers' actual practice

The majority of HEWs aged less than 30 years (77.8%) and those who have access to medicines for childhood conditions (83.3%) had better childhood pneumonia and diarrhea management practice. Moreover, greater than half of the HEWs who received supervision with a component of discussing the management of childhood conditions, and less than half of the providers who participated in the performance review clinical mentoring meetings had good actual management practice.

In the bivariate analysis, providers' age, service experience, level of education, iCCM training, supportive supervision, participation on performance review clinical mentoring meetings, knowledge of childhood pneumonia and diarrhea management, and availability of medicines were identified as candidate variables for multiple variable logistic regression at p-value<0.2. The multiple variable logistic regression analysis revealed that pneumonia and diarrhea medicines availability was associated with the actual management practices of providers. The rest of the variables didn't show associations. Correct management practice was four times more likely in providers who have access to medicines for the treatment of pneumonia and diarrhea compared to those who didn't have access (AOR=4.04, 95% CI: 1.48,11.06) (Table 5).

Table 5. Correlates of the providers' actual management practice of pneumonia and diarrhea in four regions of Ethiopia, December 2018 to February 2019

Variables	Practice		AOR (95% CI)
	Good(n,%)	Poor(n,%)	
Observations	36	94	
Age in years			
≤30	28(77.8)	70(74.5)	Ref

For peer review only

>30	8(22.2)	24(25.5)	0.61(0.22-1.75)
Service experience as HEWs in years			
≤5	14(38.9)	39(41.5)	Ref
>5	22(61.1)	55(58.5)	1.54(0.59-4.02)
Level of HEWs			
Level-3 (Certificate)	20(55.6)	59(62.8)	Ref
Level-4 (Diploma)	16(44.4)	35(37.2)	1.52(0.62-3.68)
iCCM training			
No	30(83.3)	75(79.8)	Ref
Yes	6(16.7)	19(20.2)	1.45(0.39-5.30)
Supervision with discussion on diagnosis and treatment of pneumonia and diarrhea			
No	15(41.7)	42(44.7)	Ref
Yes	21(58.6)	52(55.3)	1.13(0.44-2.87)
Supervision with a component of observing client consultations			
No	23(63.9)	58(61.7)	Ref
Yes	13(36.1)	36(38.3)	0.91(0.37-2.19)
Being participated in PRCMM			
No	19(52.8)	46(48.9)	Ref
Yes	17(47.2)	48(51.1)	0.60(0.24-1.50)
Knowledge index			
Poor knowledge	25(69.4)	67(71.3)	Ref
Good knowledge	11(30.6)	27(28.7)	1.04(0.41-2.64)
Amoxicillin, ORS and zinc availability[a]			
No	6(16.7)	38(40.4)	Ref
Yes	30(83.3)	53(59.6)	4.04(1.48-11.06)*

384 *** P<0.05 is considered statistically significant in the multivariate analysis*
385 *Abbreviations: CI, confidence interval; iCCM, integrated community case management; PRCMM, performance*
386 *review clinical mentoring meeting; HEW, health extension worker; AOR, adjusted odds ratio; Ref, reference category*
387 *[a]Out of providers who have assessed for medications availability in the health posts(n=127). Providers who had*
388 *access to all the three medicines were regarded as 'yes' and otherwise 'no'.*

389 **Discussion**

390 The study revealed that a low proportion of the providers had knowledge of the signs and
391 symptoms and management of pneumonia and diarrhea. Similarly, a low proportion of the
392 providers executed the recommended actions for the assessment and management of childhood
393 pneumonia and dehydration. There was a significant difference between the knowledge and actual
394 practice of the providers. This finding is consistent with a result among other health professionals
395 in Ethiopia (14) and frontline health workers in India (13,16).

For peer review only - http://bmjopen.bmj.com/site/about/guidelines.xhtml

Slightly greater than one-third (35.7%) of the HEWs had good knowledge of pneumonia signs and symptoms and only one-fifth of them (19.8%) had actually assessed consulting children for those signs and symptoms. The widest gap was observed between knowledge and actual practice of providers related to cough or difficulty breathing (76% vs. 47.3%) and stridor (27.8% vs. 15.1%). Besides, the incongruity between the knowledge and actual practices of the providers; the level of concordance with the clinical guidelines have remained low among the frontline providers. This has a huge impact on the clinical care outcomes related to childhood pneumonia. Poor adherence to clinical guidelines by providers in the assessment of sick child was also reported in earlier studies from low-and-middle-income countries (34,35) and in Ethiopia(27–29).

Though six out of ten (61.5%) of the providers had good knowledge of signs and symptoms of diarrhea, only four out of ten (40.2%) actually assessed those signs and symptoms in children who presented with diarrhea complaints. A wide and significant gap was observed between the knowledge and actual practice of the providers related to overall diarrhea assessment (84% vs. 41.3%) and pinch skin of the abdomen (45.9% vs. 27%). On the contrary, a large proportion of providers have practiced assessing the presence of blood in the stool (42.6% vs. 32%), and child restlessness or irritability (37.7% vs. 32%) without reporting on knowledge of the symptoms at the outset. Though the clinical guidelines recommend assessment of all the signs and symptoms of dehydration, the providers didn't actually practice them (32). This could lead to incorrect diagnosis of sick children with possible dehydration and results in missed opportunity to provide life-saving treatment for those in need of it.

The know-do gap analysis also revealed that there was a significant difference between the knowledge and actual practice of providers. Most of the providers (96.2%) have reported that they know recommended treatment options for pneumonia and diarrhea, but only a quarter of them

For peer review only - http://bmjopen.bmj.com/site/about/guidelines.xhtml

(27.7%) have actually provided treatment for children who were classified as having either pneumonia or diarrhea. Earlier studies from Ethiopia and Uganda also revealed that community health workers had poor knowledge and practice of childhood illnesses management (36–38). Contrarily, a large proportion of providers practiced counseling about medications for children who were provided treatment for pneumonia and diarrhea though only a few of them knew it as a component of sick child care.

Many factors counter play the lack of knowledge and good practice related to the assessment and management of sick children. The quality of clinical care provided to sick children is a function of the providers' knowledge, the effort exerted by the provider to apply the knowledge, and the readiness of health facilities to provide quality care in terms of medicines and supplies. In the current study, the providers' knowledge and actual practice of assessment and management of childhood pneumonia and diarrhea were associated with participation in performance review clinical mentoring meeting and the availability of medicines for pneumonia and dehydration treatment at the health posts. A previous study also revealed that clinical coaching is positively associated with good clinical practice among community health workers (39). The odds of good knowledge of pneumonia and diarrhea assessment and management was two times more likely in the providers who participated in the performance review clinical mentoring meeting. While, the odds of better correct practice were four times more likely in providers who had medicines for the treatment of childhood pneumonia and diarrhea (amoxicillin, ORS, and zinc). Other variables including the providers' characteristics (age, work or practice experience, level of training), receiving clinical training, and supportive supervision did not affect knowledge and actual practice of the HEWs. Though most of the providers were trained in iCCM, they scored low in the knowledge and practice for assessment and treatment of childhood pneumonia implying that in-

For peer review only

For peer review only - http://bmjopen.bmj.com/site/about/guidelines.xhtml

serving trainings are necessary but not sufficient drivers of correct practice(40). In another study, variables including the providers' level of education, work or practice experience, the presence of supervision, and guidelines were associated with the community health workers' knowledge of childhood illness management (37,38).

The findings of the present study have policy and practice implications. Clinical training interventions that lacked adequate practical skill component doesn't improve the competency of providers. The lack of refresher training is also a possible cause for poor performance due to the decay in knowledge. High-quality in-service training combined with mentoring, supervisions, medicines, and material support are effective to improve the quality of care(40–42). Training had high coverage in the study area, but significant number of providers are short of iCCM medicines, supplies, and technical support. Supervisions and mentoring focused on the management of childhood pneumonia and diarrhea were limited. The supervisors discussed the management of pneumonia and diarrhea in half of their visits (51.8%) and in greater than one-third (38.3%) they observed client consultations by the provider. Less than half (46%) of providers were clinically mentored according to the standard guideline, and two-thirds (65.7%) of them had access to medicines for pneumonia and diarrhea. In the study area, in-service training and supervision were not associated with knowledge, and actual practices of providers signaling a look at other approaches that improves their quality.

The study was conducted in the community health posts within the primary healthcare level, the lowest care level in the health system of Ethiopia. At this level the providers are supposed to diagnose and treat moderate cases, and refer severe cases to the health centers. This is the first study that examined the know-do gap in childhood pneumonia and diarrhea care among HEWs in Ethiopia. The know-do gap analysis was based on observations of actual management practices

For peer review only - http://bmjopen.bmj.com/site/about/guidelines.xhtml

of the providers by experienced and trained health professionals, and interviews on the knowledge assessment components. Though our findings are limited by a small sample size, the study has covered the four most populous regions of the country and is representative of the different settings in rural Ethiopia. On average the providers were observed while consulting five sick children, and this might ensure the heterogeneity of cases for both conditions. However, the clinical practices and actions of providers related to severe illnesses was not examined in the present research. The other limitation might be the Hawthorne effect i.e., a change in professional behavior of the HEWs when being observed (43). This might partly mask the actual practice of providers. But, the findings related to actual management practice in both conditions shown that the providers have performed less and in normal management situation this might be even worse.

Conclusions

The study revealed a huge know-do gap in the assessment and management of childhood pneumonia and diarrhea among the HEWs in Ethiopia. The finding affirm that knowledge-based training is necessary but not sufficient to influence the clinical performance (actual practice) of providers. The know-do gap of pneumonia and diarrhea management was determined by the presence of clinical mentoring and performance reviews and the availability of medicines. Therefore, an effort should be made to close the gap between theoretical knowledge and practice. The HEWs should be supported with mentoring, performance reviews, and the supply of medicines and commodities.

For peer review only

Acknowledgments

The authors acknowledge the administrative hierarchies and the research participants in the study area for their cooperation throughout the research process, and the time they devoted to the study in providing required information.

Authors' contributions

All authors made a significant contribution to the work reported, whether, that is, in the conception, study design, execution, acquisition of data, analysis, and interpretation, or in all these areas. DWD wrote the first draft of the manuscript, and MAW and MW revised the manuscript. All authors gave final approval of the version to be published, have agreed on the journal to which the article has been submitted, and agreed to be accountable for all aspects of the work. All authors read and approved the final manuscript.

Funding

This project was funded by Bill & Melinda Gates Foundation(INV-009691).

Disclaimer

The funder has no role in the study design, collection, management, analysis or interpretation of data.

Data availability

The datasets used and/or analyzed for the current study are not openly available because data are part of the ongoing research project and available from the corresponding author upon reasonable request (E-mail: dawit.daka86520@gmail.com; or dawit.daka@ju.edu.et).

For peer review only

For peer review only - http://bmjopen.bmj.com/site/about/guidelines.xhtml

Competing interests

All authors declare that they have no competing interests.

Patient consent for publication

Not required

References

1. United Nations Children's Fund (UNICEF). Pneumonia and diarrhoea: Tackling the deadliest diseases for the world's poorest children. United Nations Children's Fund. 2012. 1–86 p. Available from: http://www.unicef.org/eapro/Pneumonia _and_ Diarrhoea_ Report_2012.pdf

2. United Nations Children's Fund (UNICEF). One is too many: Ending child deaths from pneumonia and diarrhoea. 2016. 25 p. Available from: https://data.unicef.org/wp-content/uploads/2016/11/UNICEF-Pneumonia-Diarrhoea-report2016-web-version_final.pdf

3. IVAC at Johns Hopkins Bloomberg School of Public Health. Pneumonia and Diarrhea Progress Report 2018. 2018. Available from: http://link.springer.com/10.1023/A:1020102612073%0Ahttp://apps.who.int/gb/ebwha/pdf_files/WHA66/A66_R10-en.pdf%0Ahttp://www.who.int/diabetes/country-profiles/nga_en.pdf?ua=1

4. International Vaccine Access Center (IVAC). Pneumonia and Diarrhea Progress Report 2020. John Hopkins Bloomberg School of Public Health. 2020. Available from: https://resourcecentre.savethechildren.net/pdf/ivac_pdpr_2020.pdf/

5. Nanyonjo A, Ssekitooleko J, Counihan H, Makumbi F, Tomson G, Källander K. Impact of

For peer review only - http://bmjopen.bmj.com/site/about/guidelines.xhtml

an integrated community case management programme on uptake of appropriate diarrhoea and pneumonia treatments in Uganda: A propensity score matching and equity analysis study. Int J Equity Health. 2015;14:74. Available from: http://dx.doi.org/10.1186/s12939-015-0202-y

6. Winskill P, Mousa A, Oresanya O, Counihan H, Okell LC, Walker PG. Does integrated community case management (iCCM) target health inequities and treatment delays? Evidence from an analysis of Demographic and Health Surveys data from 21 countries in the period 2010 to 2018. J Glob Health. 2021;11:040113.

7. Young M, Wolfheim C, Marsh DR, Hammamy D. World health organization/United Nations children's fund joint statement on integrated community case management: An equity-focused strategy to improve access to essential treatment services for children. Am J Trop Med Hyg. 2012;87(SUPPL.5):6–10.

8. World Health Organization. Integrated Management of Childhood Illness (IMCI): Chart Booklet. Geneva, Switzerland: World Health Organization. 2014.

9. Kallander K, Burgess DH, Qazi SA. Early identification and treatment of pneumonia: A call to action. Lancet Glob Heal. 2016;4(1):e12–3.

10. HEFDC Group. Integrated community case management (iCCM) in sub-Saharan Africa: Successes and challenges with access, speed and quality. Thematic Review Report. 2018.

11. Bagonza J, Kibira SPS, Rutebemberwa E. Performance of community health workers managing malaria , pneumonia and diarrhoea under the community case management programme in central Uganda : a cross sectional study. Malar J. 2014;13:367.

For peer review only - http://bmjopen.bmj.com/site/about/guidelines.xhtml

12. Chinbuah MA, Abbey M, Kager PA, Gyapong M, Nonvignon J, Ashitey P, et al. Assessment of the adherence of community health workers to dosing and referral guidelines for the management of fever in children under 5 years: a study in Dangme West District, Ghana. Int Health. 2013;5:148–56.

13. Ray Saraswati L, Baker M, Mishra A, Bhandari P, Rai A, Mishra P, et al. ‘Know-Can’ gap: gap between knowledge and skills related to childhood diarrhoea and pneumonia among frontline workers in rural Uttar Pradesh, India. Trop Med Int Heal. 2020;25(4):454–66.

14. Gage AD, Kruk ME, Girma T, Lemango ET. The know-do gap in sick child care in Ethiopia. PLoS One. 2018;13(12):e0208898.

15. Tielsch JM. The Know, Do, and Quality Gaps in International Maternal and Child Health Care Interventions. JAMA Pediatr. 2015 Apr 1;169(4):313–4. Available from: https://doi.org/10.1001/jamapediatrics.2014.3741

16. Mohanan M, Vera-Hernández M, Das V et al. The Know-Do Gap in Quality of Health Care for Childhood Diarrhea and Pneumonia in Rural India. Jama Pediatr. 2015;169(4):349–57.

17. Mungai IG, Baghel SS, Soni S, Vagela S, Sharma M, Diwan V, et al. Identifying the know-do gap in evidence-based neonatal care practices among informal health care providers—a cross-sectional study from Ujjain, India. BMC Health Serv Res. 2020;20:966.

18. Wanduru P, Tetui M, Tuhebwe D, Ediau M, Okuga M, Nalwadda C, et al. The performance of community health workers in the management of multiple childhood

For peer review only

For peer review only - http://bmjopen.bmj.com/site/about/guidelines.xhtml

infectious diseases in Lira, northern Uganda – a mixed methods cross-sectional study. Glob Health Action. 2016 Dec 1;9:33194. Available from: https://doi.org/10.3402/gha.v9.33194

19. Rabbani F, Perveen S, Aftab W, Zahidie A, Sangrasi K, Qazi SA. Health workers' perspectives, knowledge and skills regarding community case management of childhood diarrhoea and pneumonia: A qualitative inquiry for an implementation research project "nigraan" in District Badin, Sindh, Pakistan. BMC Health Serv Res. 2016;16:462. Available from: http://dx.doi.org/10.1186/s12913-016-1699-5

20. Izudi J, Anyigu S, Ndungutse D. Adherence to Integrated Management of Childhood Illnesses Guideline in Treating South Sudanese Children with Cough or Difficulty in Breathing. Menahem S, editor. Int J Pediatr. 2017;2017:5173416. Available from: https://doi.org/10.1155/2017/5173416

21. Miller NP, Amouzou A, Tafesse M, Hazel E, Legesse H, Degefie T, et al. Integrated Community Case Management of Childhood Illness in Ethiopia : Implementation Strength and Quality of Care. Am J Trop Med Hyg. 2014;91(2):424–34.

22. Sinyangwe C, Graham K, Nicholas S, King R, Mukupa S, Källander K, et al. Assessing the quality of care for pneumonia in integrated community case management: A cross-sectional mixed methods study. PLoS One. 2016;11(3):e0152204.

23. Baynes C, Mboya D, Likasi S, Maganga D, Pemba S, Baraka J, et al. Quality of sick child-care delivered by community health workers in Tanzania. Int J Heal Policy Manag. 2018;7(12):1097–109. Available from: https://doi.org/10.15171/ijhpm.2018.63

24. Berhanu A, Alemayehu M, Daka K, Binu W, Suleiman M. Utilization of Integrated

For peer review only

For peer review only - http://bmjopen.bmj.com/site/about/guidelines.xhtml

Community Case Management of Childhood Illnesses at Health Posts in Southern Ethiopia. Pediatr Heal Med Ther. 2020;Volume 11:459–67.

25. Assefa Y, Gelaw YA, Hill PS, Taye BW, Van Damme W. Community health extension program of Ethiopia, 2003–2018: successes and challenges toward universal coverage for primary healthcare services. Global Health. 2019;15(1):24. Available from: https://doi.org/10.1186/s12992-019-0470-1

26. Legesse H, Degefie T, Hiluf M, Sime K, Tesfaye C, Abebe H, et al. National scale-up of integrated community case management in rural Ethiopia: Implementation and early lessons learned. Ethiop Med J. 2014;52(December):15–26.

27. Wolde D, Daka, Wordofa MA, Woldie M, Persson Å, Berhanu D. Quality of clinical assessment and management of sick children by Health Extension Workers in four regions of Ethiopia : A cross-sectional survey. PLoS One. 2020;15(9):e0239361.

28. Beyene H, Hailu D, Tadele H, Persson LÅ, Berhanu D. Insufficient referral practices of sick children in Ethiopia shown in a cross-sectional survey. Acta Paediatr Int J Paediatr. 2020;109(9):1867–74.

29. Getachew T, Mekonnen S, Yitayal M, Persson LÅ, Berhanu D. Health Extension Workers' diagnostic accuracy for common childhood illnesses in four regions of Ethiopia: a cross-sectional study. Acta Paediatr Int J Paediatr. 2019;108(11):2100–6. Available from: https://onlinelibrary.wiley.com/doi/epdf/10.1111/apa.14888

30. Berhanu D, Okwaraji YB, Belayneh AB, Lemango ET, Agonafer N, Birhanu BG et al. Protocol for the evaluation of a complex intervention aiming at increased utilization of primary child health services in Ethiopia: A before and after study in intervention and

For peer review only

For peer review only - http://bmjopen.bmj.com/site/about/guidelines.xhtml

comparison areas. BMC Health Serv Res. 2010;20:339.

31. World Health Organization. Health facility survey : tool to evaluate the quality of care delivered to sick children attending outpatient facilities. Geneva PP - Geneva: World Health Organization; Available from: https://apps.who.int/iris/handle/10665/42643

32. Ministry of Health of Ethiopia. The Integrated Community Case Management (iCCM) Chart Booklet Ethiopia. 2014. Available from: http://lifesavingcommodities.org/wp-content/uploads/2014/08/iCCM-English-Chartbooklet-August-2013.pdf

33. Tesema AG, Peiris D, Abimbola S, Ajisegiri WS, Narasimhan P, Mulugeta A, et al. Community health extension workers' training and supervision in Ethiopia: Exploring impact and implementation challenges for non-communicable disease service delivery. PLOS Glob Public Heal. 2022 Nov 9;2(11):e0001160. Available from: https://doi.org/10.1371/journal.pgph.0001160

34. Gilroy KE, Callaghan-koru JA, Cardemil C V, Nsona H, Amouzou A, Mtimuni A, et al. Quality of sick child care delivered by Health Surveillance Assistants in Malawi. Health Policy Plan. 2013;28:573–85.

35. Kruk RME, Gaga AD, Mbaruku GM, Leslie HH. Content of Care in 15,000 Sick Child Consultations in Nine Lower-Income Countries. Heal Serv Res. 2018;12842. Available from: https://doi.org/10.1111/1475-6773.12842).

36. Bagonza J, Kibira SPS, Rutebemberwa E. Performance of community health workers managing malaria, pneumonia and diarrhoea under the community case management programme in central Uganda: A cross sectional study. Malar J. 2014;13(1):1–10.

For peer review only - http://bmjopen.bmj.com/site/about/guidelines.xhtml

37. Befekadu A, Yitayal M. Knowledge and practice of health extension workers on drug provision for childhood illness in west Gojjam, Amhara, Northwest Ethiopia. BMC Public Health [Internet]. 2020;20:496. Available from: https://doi.org/10.1186/s12889-020-08602-y

38. Wanduru P, Tetui M, Tuhebwe D, Ediau M, Okuga M, Nalwadda C, et al. The performance of community health workers in the management of multiple childhood infectious diseases in Lira, northern Uganda - A mixed methods cross-sectional study. Glob Health Action. 2016;9(1).

39. Shiroya-Wandabwa M, Kabue M, Kasungami D, Wambua J, Otieno D, Waka C, et al. Coaching community health volunteers in integrated community case management improves the care of sick children under-5: Experience from Bondo, Kenya. Int J Integr Care. 2018;18(4):1–11.

40. Leslie HH, Gage A, Nsona H, Hirschhorn LR, Kruk ME. Training And Supervision Did Not Meaningfully Improve Quality Of Care For Pregnant Women Or Sick Children In Sub-Saharan Africa. Health Aff. 2016 Sep 1;35(9):1716–24. Available from: https://doi.org/10.1377/hlthaff.2016.0261

41. Bluestone J, Troncoso E, Fitzgerald L, Nagbe L, Tetteh G, Hellar A, et al. Three Pivots for Improving Health Care Provider Performance. Glob Heal Sci Pract. 2022 Feb 28;10(1):e2100625. Available from: http://www.ghspjournal.org/content/10/1/e2100625.abstract

42. Desta BF, Beshir IA, Tefera BB, Argaw MD, Demeke HZ, Kibret MA. Does frequency of supportive supervisory visits influence health service delivery?—Dose and response

For peer review only

For peer review only - http://bmjopen.bmj.com/site/about/guidelines.xhtml

study. PLoS One. 2020 Jun 16;15(6):e0234819. Available from: https://doi.org/10.1371/journal.pone.0234819

43. Oswald D. Handling the Hawthorne effect: The challenges surrounding a participant observer. Rev Soc Stud. 2014 Oct 1;1:53–74.

For peer review only

S1 Table. Medical equipment, job aids, and medicine availability at the health posts of providers in four regions of Ethiopia, December 2018 to February 2019 (N=147)

Medical equipment, job aids, and Medicines	n(%)
Medical equipment	
Thermometer	121(82.3)
Infant scale	115(78.2)
Weighing sling	116(78.9)
Tape measures	79(53.7)
Blood pressure apparatus	69(46.9)
Stethoscopes	98(66.7)
Watch or clock (timer)	16(10.9)
Examination couch	102(69.4)
Mid-upper-arm circumference tape	147(100.0)
Basic equipment availability index[a]	44(29.9)
Job aids	
iCCM register (2-59 months old children)	145(98.6)
Stock/bin cards to track medicines	97(66.0)
Medicines request and resupply forms	95(64.6)
Chart booklets	138(93.9)
Medicines	
Amoxicillin	115(78.2)
Oral rehydration solution	137(93.2)
Zinc	125(85.0)
Oral rehydration solution and zinc	117(79.6)
Paracetamol	94(63.9)
Amoxicillin, oral rehydration solution and zinc	96(65.3)

[a] *Basic equipment availability index: Availability of all of the following for the same provider: thermometer, infant scale, weighing sling, stethoscope, and BP apparatus*

For peer review only

S2 Table. Providers knowledge of childhood pneumonia and diarrhea management in four regions of Ethiopia, December 2018 to February 2019 (N=274)

Knowledge of-	n	%(95% CI)
Pneumonia signs and symptoms		
Good knowledge	97	35.4(29.9-41.2)
Poor knowledge	177	64.6(58.8-70.1)
Pneumonia management approaches		
Good knowledge	67	24.5(19.6-29.8)
Poor knowledge	207	75.5(70.2-80.4)
Pneumonia signs and symptoms and management approaches		
Good knowledge	108	39.4(33.7-45.3)
Poor knowledge	166	60.6(54.7-66.3)
Diarrhea signs and symptoms		
Good knowledge	57	20.8(16.3-25.9)
Poor knowledge	217	79.2(74.1-83.7)
Diarrhea management approaches		
Good knowledge	66	24.1(19.3-29.4)
Poor knowledge	208	75.9(70.6-80.7)
Diarrhea signs and symptoms and management approaches		
Good knowledge	78	28.5(23.4-34.0)
Poor knowledge	196	71.5(66.0-76.6)
Both illnesses sign and symptoms and management approaches		
Good knowledge	74	27.0(22.0-32.5)
Poor knowledge	200	73.0(67.5-78.0)

For peer review only

STROBE Statement—Checklist of items that should be included in reports of ***cross-sectional studies***

	Item No	Recommendation	Page No
**Title and abstract**	1	(*a*) Indicate the study's design with a commonly used term in the title or the abstract	2
		(*b*) Provide in the abstract an informative and balanced summary of what was done and what was found	2&3
**Introduction**			
Background/rationale	2	Explain the scientific background and rationale for the investigation being reported	3-5
Objectives	3	State specific objectives, including any prespecified hypotheses	5
**Methods**			
Study design	4	Present key elements of study design early in the paper	5
Setting	5	Describe the setting, locations, and relevant dates, including periods of recruitment, exposure, follow-up, and data collection	5-6
Participants	6	(*a*) Give the eligibility criteria, and the sources and methods of selection of participants	6
Variables	7	Clearly define all outcomes, exposures, predictors, potential confounders, and effect modifiers. Give diagnostic criteria, if applicable	7-8
Data sources/ measurement	8*	For each variable of interest, give sources of data and details of methods of assessment (measurement). Describe comparability of assessment methods if there is more than one group	6-7
Bias	9	Describe any efforts to address potential sources of bias	7
Study size	10	Explain how the study size was arrived at	6
Quantitative variables	11	Explain how quantitative variables were handled in the analyses. If applicable, describe which groupings were chosen and why	9
Statistical methods	12	(*a*) Describe all statistical methods, including those used to control for confounding	9
		(*b*) Describe any methods used to examine subgroups and interactions	
		(*c*) Explain how missing data were addressed	
		(*d*) If applicable, describe analytical methods taking account of sampling strategy	
		(*e*) Describe any sensitivity analyses	
**Results**			
Participants	13*	(a) Report numbers of individuals at each stage of study—eg numbers potentially eligible, examined for eligibility, confirmed eligible, included in the study, completing follow-up, and analysed	10
		(b) Give reasons for non-participation at each stage	
		(c) Consider use of a flow diagram	
Descriptive data	14*	(a) Give characteristics of study participants (eg demographic, clinical, social) and information on exposures and potential confounders	10-11
		(b) Indicate number of participants with missing data for each variable of interest	
Outcome data	15*	Report numbers of outcome events or summary measures	13-16
Main results	16	(*a*) Give unadjusted estimates and, if applicable, confounder-adjusted estimates and their precision (eg, 95% confidence interval). Make clear which confounders were adjusted for and why they were included	16-18

For peer review only

		(*b*) Report category boundaries when continuous variables were categorized	
		(*c*) If relevant, consider translating estimates of relative risk into absolute risk for a meaningful time period	
Other analyses	17	Report other analyses done—eg analyses of subgroups and interactions, and sensitivity analyses	
**Discussion**			
Key results	18	Summarise key results with reference to study objectives	18
Limitations	19	Discuss limitations of the study, taking into account sources of potential bias or imprecision. Discuss both direction and magnitude of any potential bias	20-21
Interpretation	20	Give a cautious overall interpretation of results considering objectives, limitations, multiplicity of analyses, results from similar studies, and other relevant evidence	18-21
Generalisability	21	Discuss the generalisability (external validity) of the study results	18-21
**Other information**			
Funding	22	Give the source of funding and the role of the funders for the present study and, if applicable, for the original study on which the present article is based	22

*Give information separately for exposed and unexposed groups.

Note: An Explanation and Elaboration article discusses each checklist item and gives methodological background and published examples of transparent reporting. The STROBE checklist is best used in conjunction with this article (freely available on the Web sites of PLoS Medicine at http://www.plosmedicine.org/, Annals of Internal Medicine at http://www.annals.org/, and Epidemiology at http://www.epidem.com/). Information on the STROBE Initiative is available at www.strobe-statement.org.

For peer review only

BMJ Open

Know-do gap for sick child care and drivers of knowledge and practice among health extension workers in four regions of Ethiopia: a cross-sectional study

Journal:	*BMJ Open*
Manuscript ID	bmjopen-2022-069698.R3
Article Type:	Original research
Date Submitted by the Author:	10-Aug-2023
Complete List of Authors:	Daka, Dawit; Jimma University, Health Policy and Management<br>Wordofa, Muluemebet; Jimma University, Department of Population & Family Health<br>Woldie, Mirkuzie; Jimma University, Health Policy and Management
**Primary Subject Heading**:	Health services research
Secondary Subject Heading:	Health services research
Keywords:	Community child health < PAEDIATRICS, Quality in health care < HEALTH SERVICES ADMINISTRATION & MANAGEMENT, INFECTIOUS DISEASES, Public health < INFECTIOUS DISEASES

SCHOLARONE™ Manuscripts

I, the Submitting Author has the right to grant and does grant on behalf of all authors of the Work (as defined in the below author licence), an exclusive licence and/or a non-exclusive licence for contributions from authors who are: i) UK Crown employees; ii) where BMJ has agreed a CC-BY licence shall apply, and/or iii) in accordance with the terms applicable for US Federal Government officers or employees acting as part of their official duties; on a worldwide, perpetual, irrevocable, royalty-free basis to BMJ Publishing Group Ltd ("BMJ") its licensees and where the relevant Journal is co-owned by BMJ to the co-owners of the Journal, to publish the Work in this journal and any other BMJ products and to exploit all rights, as set out in our licence.

The Submitting Author accepts and understands that any supply made under these terms is made by BMJ to the Submitting Author unless you are acting as an employee on behalf of your employer or a postgraduate student of an affiliated institution which is paying any applicable article publishing charge ("APC") for Open Access articles. Where the Submitting Author wishes to make the Work available on an Open Access basis (and intends to pay the relevant APC), the terms of reuse of such Open Access shall be governed by a Creative Commons licence – details of these licences and which Creative Commons licence will apply to this Work are set out in our licence referred to above.

Other than as permitted in any relevant BMJ Author's Self Archiving Policies, I confirm this Work has not been accepted for publication elsewhere, is not being considered for publication elsewhere and does not duplicate material already published. I confirm all authors consent to publication of this Work and authorise the granting of this licence.

For peer review only

Know-do gap for sick child care and drivers of knowledge and practice among health extension workers in four regions of Ethiopia: a cross-sectional study

Dawit Wolde Daka[1*], Muluemebet Abera Wordofa[2], and Mirkuzie Woldie[3]

[1]Faculty of Public Health, Department of Health Policy and Management, Jimma University, Jimma, Ethiopia

[2]Faculty of Public Health, Department of Population and Family Health, Jimma University, Jimma, Ethiopia

[3] Adjunct Professor, Faculty of Public Health, Department of Health Policy and Management, Jimma University, Jimma, Ethiopia

*Corresponding Author

E-mail: dawit.daka86520@gmail.com or dave86520@gmail.com

https://orcid.org/0000-0001-5465-6345

For peer review only

Abstract

Objective: Though efforts were made to expand community-based sick child healthcare in Ethiopia, the quality of care provided remained low. Improving quality of care requires understanding providers' knowledge of appropriate care and their actual execution of tasks. This study examined gap between what health extension workers(HEWs) knew and did during the management of sick children in Ethiopia.

Design: Facility-based cross-sectional study was conducted.

Setting: The study was carried out in 52 districts across four regions in Ethiopia.

Participants: We interviewed 274 HEWs and performed observations of consultations done by 150 HEWs supplemented with facility assessment from December 2018 to February 2019.

Outcome: We compared providers' knowledge and performance in the management of childhood pneumonia and diarrhea. Know-do gap implies the difference in proportion between knowledge and actual practice of HEWs. Logistic regression was used to identify predictors of knowledge and actual practice.

Results: Providers' correct knowledge ranged from 27.8% to 76.0% for signs and symptoms of pneumonia, and 32.0% to 84% for dehydration signs. Their actual practices ranged from 15.1% to 47.3% for pneumonia, and 27.0% to 42.6% for dehydration. The correct knowledge and actual practices for pneumonia and dehydration management were 88.3% vs 15.6% and 93.9% vs 51.3%, respectively. There was significant know-do gap in assessments (16.7%, p=0.002) and management of childhood conditions (68.5%, p<0.0001). Mentorships were associated with providers' knowledge of clinical management, while medicines availability was associated with their actual management practice.

For peer review only - http://bmjopen.bmj.com/site/about/guidelines.xhtml

Conclusions: While knowledge and actual practice for assessment and management of pneumonia and dehydration ranged from very low to high, what is more concerning is the huge know-do gap among HEWs. Our findings suggest that knowledge-based training is necessary but not sufficient for ensuring correct assessment and management of sick children by HEWs. Continuous support through mentorships and the supply of commodities are critically needed.

Keywords: Knowledge, actual practice, know-do gap, pneumonia, diarrhea, assessment, management, health extension workers, Ethiopia

Strengths and limitations of this study

- The study used a validated tool that was tested in various low-and-middle-income settings for the evaluation of the quality of sick child care.
- Observations of sick child consultations were performed by clinicians who had received refresher training on integrated community case management (iCCM).
- Though sample size was small for the know-do gap analysis, the study has covered the four largest regions of the country.
- The observation method used in this study may have led to the Hawthorne effect possibly masking the actual practice due to a change in the professional behavior of the health extension workers observed.

Introduction

Pneumonia and diarrhea remain the two major childhood illnesses that contributed to the larger portion of under-five mortality worldwide (1,2). Globally, over 1.23 million children died of pneumonia and diarrhea before reaching their fifth birthday in 2018. Seventy percent of all pneumonia and diarrhea deaths in children under the age of five years have occurred in 15 countries

For peer review only - http://bmjopen.bmj.com/site/about/guidelines.xhtml

of the world alone, and Ethiopia is one among them. In Ethiopia, 44, 692 under-five children's deaths were attributed to pneumonia and diarrhea in 2017 which accounted for 13.6 deaths per 1000 live births. Out of sick children with suspected pneumonia, only 31% were taken to an appropriate care provider and 7% received the recommended antibiotics. Similarly, of those under five children with diarrhea, 30% have received oral rehydration solution (ORS) and 33% were provided with zinc supplements (3,4).

The integrated community case management (iCCM) approach to common childhood illnesses is a key strategy for improving the treatment of potentially life-threatening illnesses in children (5–7). The iCCM guideline recommends that all children 2-59 months of age are first assessed for general danger signs, then for cough or difficult breathing, diarrhea, fever, ear problem, acute malnutrition, and anemia. Then children are classified based on assessment results and treated according to the standard of care(8). Adherence to guidelines by front-line providers is critical to improving the quality of care for sick children and improving treatment outcomes (9). However, studies have shown that providers' adherence to the clinical standards of sick children assessment and management is low in most low- and middle-income settings (10–12) and a significant know-do gap in clinical practices was common among the health care providers(13,14).

The know-do gap, failure to apply existing knowledge to improve health outcome, has a huge impact on the effort made towards reducing under-five mortalities attributed to poor quality or substandard care. It results in inconsistencies in care and inefficiencies in workflows. Though much is known about the evidence-based interventions related to maternal and child healthcare globally, it is not practiced as intended and the practices vary across the different settings (15). Most of the know-do gap is a result of inadequately skilled providers, lack of key equipment and medicines, and financial resources(14,16,17). Other factors impacting on the likelihood of

For peer review only - http://bmjopen.bmj.com/site/about/guidelines.xhtml

providers practicing what they know include provider background characteristics (sex, age, working experience, level of education), extent of community support, and presence support in the form of regular trainings, mentoring, and supportive supervision(11,13,18–20). On the other hand, if community health workers are supported adequately, they can assess and treat sick children according to the protocol in the majority of the cases (21–23).

Narrowing the know-do gap is critical to attaining child survival goals and the targets set under the Global Action Plan for the Prevention and Control of Pneumonia and Diarrhea (GAPPD) in 2025. One of the core interventions outlined in the action plan was the appropriate management of sick children who are at risk of serious illnesses or death due to these two diseases (pneumonia and diarrhea). It is aimed to increase treatment coverage for children with suspected pneumonia including care by an appropriate healthcare provider and antibiotics, and treatment coverage for children with diarrhea including treatments with ORS and zinc supplements to 90% (4).

In Ethiopia, the integrated community case management services implementation was started in 2010 as part of the health extension program(21,24). Currently, the program is implemented in more than 18,000 health posts nationally(25). Despite huge investments and high coverage of clinical training (21,26), studies revealed that the quality of care is low in the health posts of Ethiopia in terms of sick children assessment, classification, treatment, and referral practices(27–29). On the other hand, little is known about the know-do gap in sick child assessment and management, and the drivers among health extension workers(HEWs) in Ethiopia. Therefore, this paper aimed to examine the level of agreement between the knowledge the HEWs had about assessment and management and their actual practice of childhood pneumonia and diarrhea.

For peer review only - http://bmjopen.bmj.com/site/about/guidelines.xhtml

Methods

Study setting and period

The study was conducted in the health posts of 52 districts across the four most populous regions of Ethiopia: Oromia, Amhara, Southern Nations Nationalities and People's (SNNP), and Tigray from December 2018 to February 2019. The four regions and the districts were selected by the Ministry of Health, Regional Health Bureaus, and the implementing partners in 2016 for the planned intervention. The intervention districts were selected for having poor primary health service utilization indicators for under five children and the comparison districts were identified based on their similarity in population size and key maternal and child health indicators. This paper was part of the larger Dagu end-line survey, a survey that aimed to estimate the effectiveness of the optimizing health extension program (OHEP) in Ethiopia and the baseline survey was conducted from December 2016 to February 2017. The protocol for the OHEP evaluation was published elsewhere (30).

Study design and participants

A facility-based cross-sectional study was conducted among health extension workers that fulfilled the inclusion criteria. Two-stage stratified sampling strategy was used to select the study participants from the districts. In the first stage enumeration areas (EAs) were selected from the study districts using the list of EAs from the 2007 Ethiopian Housing and Population Census as the sampling frame. The cumulative population size to clusters across the study areas was calculated and 200 EAs were selected with a probability proportional to population size (PPS). Each EA formed a cluster and each cluster constituted a primary sampling unit. In the second stage, all health posts serving the catchment population of the selected EAs were included in the study.

For peer review only - http://bmjopen.bmj.com/site/about/guidelines.xhtml

Similarly, all HEWs who were present during the survey period were included in the study. Moreover, the sick children who visited the selected health posts to seek care from HEWs at the time of data collection were included.

Measurement

Structured interviews, observation, and health post-assessment questionnaires were used to collect data. The questionnaires were developed based on the World Health Organization (WHO) tool for Integrated Management of Childhood Illnesses (IMCI) sick child quality assessment in primary healthcare facilities(31) and the national iCCM guidelines(32). The health provider's interview questions included items such as background characteristics, iCCM training, supportive supervision, clinical mentorships, and knowledge of assessment and management of sick children 2-59 months. The observation questionnaire captured data on the reason for visits, assessment, classification, treatment, referral, and counseling. The facility assessment tool had sections of health posts infrastructure, medical equipment, medicines, and job aids. All the questionnaires were uploaded on tablets (CSPro 7.1) for data collection.

Data collectors with a qualification of bachelor's degree in health science were recruited and involved in data collection. Likewise, supervisors with a health background and qualifications with a bachelor of science and above have participated. Data collectors and supervisors were trained for 10 days on the questionnaires, data collection techniques, how to use tablets, and research ethics. Clinical observations of providers were conducted following interviews and facility assessments. The supervisors have monitored the daily data collection process at the field level and provided support. Moreover, a data manager at the central office monitored data on the server and provided feedback to the field team.

For peer review only - http://bmjopen.bmj.com/site/about/guidelines.xhtml

Study variables

The outcome variable was the know-do gap of child care related to pneumonia and diarrhea assessment and management. The providers' knowledge data were obtained from interviews, and the actual practice data were collected through observation by clinicians. Know-do gap was the difference between the knowledge of HEWs about the signs and symptoms and management of pneumonia and diarrhea and their ability to correctly assess those signs and symptoms when children are presented with the illnesses. A provider was regarded as having a know-do gap in the following conditions: (a) if correctly named the signs and symptoms of the illnesses but failed to actually identify them; (b) if unable to correctly named the signs and symptoms of the illnesses but able to identify them, and (c) if unable to correctly named the signs and symptoms of the illnesses as well as unable to correctly identify them. On the other hand, if the provider correctly named the signs and symptoms, and also correctly identified them, then he/she was regarded as with no know-do gap. Similarly, the above conditions were applied to the know-do gap of pneumonia and dehydration management by the providers.

A total of 11 knowledge items were used, each having 'yes=1' and 'no=0' responses. Providers were asked to respond to questions without prompts and the responses were recorded from the pre-defined list. Then the interviewer recorded the responses of the providers. The knowledge questions were: what are the main signs and symptoms of pneumonia in children 2-59 months (3 responses)? When a child presents with signs and symptoms of pneumonia, what initial measures do you take (2 responses)? What are the main signs and symptoms of diarrhea in children 2-59 months (4 responses)? When a child presents with signs and symptoms of diarrhea, what initial measures do you take (2 responses)?. The initial measures taken by providers for pneumonia have

For peer review only - http://bmjopen.bmj.com/site/about/guidelines.xhtml

included prescribing antibiotics and counseling on medications, and for diarrhea, the actions were providing ORS and zinc, and counseling on medications.

We have constructed the knowledge index based on the Ethiopian university students' grading system with a few modifications as there was no validated standard to evaluate providers' knowledge. The Ethiopian university grading system is excellent [85-100%], very good [75 to 85%), good [60-75%), satisfactory [50-60%), unsatisfactory [45,50%), and very poor (below 45%). The providers who have scored 60% and above were categorized as having 'Good knowledge/knowledgeable=1', and otherwise 'poor knowledge/not knowledgeable=0'.

Hence, the HEWs were labeled knowledgeable if they have correctly responded to ≥60% of the knowledge items (3 and more items out of 5 for pneumonia; 4 and more items out of 6 for diarrhea; 7 and more items out of 11 for average knowledge). All scores below 60% were regarded as poor knowledge for the individual and average scores.

The ability to correctly assess and manage children (the actual practice of providers) for pneumonia and diarrhea index was constructed. The providers who correctly assessed children that presented with respiratory and diarrhea complaints according to the iCCM guidelines were categorized as 'correct practice for assessment=1' and otherwise 'incorrect practice for assessment=0'. Likewise, the providers who correctly treated children who have been classified as having pneumonia or diarrhea at the outset according to the iCCM guidelines were categorized as 'correct practice for treatment=1' and otherwise 'incorrect practice for treatment=0'. The providers were observed while consulting more than one sick children and hence, correct assessment and treatment for both conditions were defined as being able to correctly assess and treat all children according to the iCCM guideline.

For peer review only - http://bmjopen.bmj.com/site/about/guidelines.xhtml

The tasks of pneumonia assessment according to the iCCM guidelines were ‘ask for the presence of cough or difficult breathing, check chest-indrawing, and listen for stridor’. The tasks for diarrhea assessment were ‘ask for the presence of diarrhea, ask the presence of blood in the stool, check child restlessness or irritability, and check whether skin pinch goes back slowly or very slowly’. The task of pneumonia treatment was providing antibiotics to sick children classified as having pneumonia, and the task of diarrhea treatment was providing ORS and zinc to those sick children who were classified as having diarrhea. The providers that correctly assessed children for pneumonia and diarrhea were categorized as ‘correct practice for assessment=1’ and otherwise ‘incorrect practice for assessment=0’. Those providers who treated all children who were classified as having pneumonia, and diarrhea with antibiotics, and ORS and Zinc were labeled as ‘correct practice for treatment=1’ and the providers who didn’t provide recommended treatments in either of the conditions were labeled as ‘incorrect practice for treatment=0’.

The explanatory variables of knowledge and actual practices were provider characteristics (age, working experience as HEWs, training level of HEWs (level-3 and level-4)), iCCM training status, presence of supportive supervision and mentorships, and presence of medicines and supplies. Level-3 HEWs are those who graduated with a certificate after a one-year pre-service training on the 17 packages of the health extension program, and level-4 HEWs are those who have level-3 certificate and got one additional year training at a technical college(25,33). The 17 packages of the health extension program are categorized into four components or areas as (a) Family health services (maternal and child health, family planning, immunization, adolescent reproductive health, nutrition); (b) Disease prevention and control (HIV/AIDS, Tuberculosis, malaria, first aid); (c) Hygiene and environmental sanitation (proper and safe excreta disposal, proper and safe solid and liquid waste management, water supply safety measures, food hygiene and safety measures,

For peer review only - http://bmjopen.bmj.com/site/about/guidelines.xhtml

healthy home environment, arthropods and rodents control, personal hygiene); and (d) Health education and communication(25).

Data processing and analysis

Primarily, the HEWs module data set and the observation module data set were linked using individual ID and then linked to the health facility assessment data set. The health facility assessment data included information on health post infrastructure, availability of medical equipment, medicines, and job aids. Data values were cleaned for inconsistency and incompleteness. Coding and re-coding of variables were done, and the indices were computed as appropriate. The know-do gap analysis was conducted for those HEWs who have been observed while consulting sick children, and analysis for knowledge about sick child management was conducted for all the HEWs included in the study. The knowledge analysis was conducted on 274 HEWs, and from these the know-do gap analysis was performed with 150 HEWs who have consulted sick children on the date of the survey. Due to the smaller sample size, we were unable to do regression for know-do gap and preferred to perform a separate model for knowledge and practice of the HEWs who did sick child consultations.

Descriptive statistics were performed to analyze and describe the characteristics of providers, other explanatory variables, and the outcome variable. A 95% confidence interval was constructed for the outcome variable and some of the explanatory variables. To assess whether there exist significant differences between the proportion of providers with the correct knowledge and correct practice, we have conducted two-sample z-tests for differences in proportions. Bivariate logistic regression was used to select candidate explanatory variables for multiple variable logistic regression at p-value<0.2 and multiple variable logistic regression was used to analyze the association between explanatory variables with knowledge, and actual management practice of the

For peer review only - http://bmjopen.bmj.com/site/about/guidelines.xhtml

providers. The variables considered in this analysis were provider age, years of experience, level of education (level-3 and level-4), iCCM training status, presence of supportive supervision and mentorships, and presence of medicines and supplies. Those variables with a p-value<0.05 were considered statistically significant. All analysis was performed using SPSS version 20 (IBM corporation, New York, USA).

Ethical consideration

Research ethical approval was obtained from the Ethiopian Public Health Institute (protocol number SERO-012-8-2016; 001 August 2016), the London School of Hygiene and Tropical Medicine (protocol number 11235, June 2016), and Jimma University (protocol number IHRPGD/472/2018, August 2018). A support letter was secured from the regional health bureaus and zonal health offices. Informed written consent was taken from providers and for observation, consent was also taken from caregivers. For caregivers who were unable to read and write, witness was presented and signatures appended on the consent form. The confidentiality of providers and patient information was maintained properly through the use of codes and computer-based data were secured with passwords. Data will be used for the intended research purpose and will not be shared with a third party.

Patient and public involvement

Patient and/or the public were not involved in the design or conduct, or reporting or dissemination plans of this research.

For peer review only

For peer review only - http://bmjopen.bmj.com/site/about/guidelines.xhtml

Results

Description of background characteristics

From the total of 274 HEWs interviewed, 150 examined sick children and were included in the know-do gap and actual practice analysis. While 274 HEWs were used in the knowledge analysis. The majority (78.8%) of providers had received iCCM training and out of which the larger proportion had received the training within the 12 months preceding the date of the survey (187 or 86.6%). Though the majority of the HEWs had received supervision within the 3 months preceding the survey date (71.9%), only 33.9% had been visited regularly. Furthermore, less than half (46%) of the HEWs participated in the performance review clinical mentoring meetings (PRCMM) that were held 6 months preceding the survey date. The details for the background characteristics of the HEWs included in the study is provided in Table 1.

Table 1. Background characteristics of the health extension workers in four regions of Ethiopia, December 2018 to February 2019

Variables	n(%)
Age in years	
≤30	187(68.2)
>30	87(31.8)
Level of Training[a]	
Level-3 (Certificate)	154(56.2)
Level-4 (Diploma)	120(43.8)
Work experience as a HEW in years	
≤5	106(38.7)
6-10	63(23.0)
>10	105(38.3)
iCCM training	
Yes	216(78.8)
No	58(21.2)
Received supportive supervision in the last 3 months	
Yes	197(71.9)

For peer review only - http://bmjopen.bmj.com/site/about/guidelines.xhtml

No	77(28.1)
Number of visits (in the last 3 months)	
No visit	77(28.1)
One to two	104(38.0)
Three and more	93(33.9)
Contents of supportive supervision[b]	
Discussing diagnosis and treatment of suspected pneumonia	132(67.0)
Discussing diagnosis and treatment of suspected diarrhea	139(70.6)
Observing record keeping	187(94.9)
Checking the register for consistency and completeness	183(92.9)
Checking supplies including manuals, job aids, and request forms	146(74.1)
Delivering supplies including manuals, job aids, and request forms	123(62.4)
Observing client consultation with providers	99(50.3)
Providing written feedback	131(66.5)
Participated in PRCMM in the last 6 months	
Yes	126(46.0)
No	148(54.0)
Region	
Amhara	130(47.4)
Oromia	71(25.9)
SNNP	37(13.5)
Tigray	36(13.1)

Abbreviations: iCCM, integrated community case management; PRCMM, performance review clinical mentoring meeting; SNNP, southern nations, nationalities, and people
[a]Level of Training: Health extension workers (HEW) level of training categories: Level-3 HEWs are those who graduated with a certificate after a one-year pre-service training on the 17 packages of the Health Extension Program. Level-4 HEWs are those who graduated with a diploma after one year of additional education at a technical college.
[b]Out of the total health extension workers that have received supervision in the last 3 months (N=197)

The HEWs have consulted a total of 685 sick children, with an average of 5 sick children consultations per HEW (standard deviation 1.6). The minimum and maximum consultations were 1 and 6 respectively, and the majority, 127(84.7%), of the HEWs have consulted three or more sick children. Out of the total sick children who visited the HEWs, 628(91.7%) were older than 6 months. Three hundred sixteen (46%) sick children had respiratory complaints and 302(44%) had diarrhea related complaints.

For peer review only - http://bmjopen.bmj.com/site/about/guidelines.xhtml

Medical equipment, job aids, and medicines availability

A total of 121 (82.3%) providers had thermometers, 115(78.2%) had infant scales, 116(78.9%) had weighing slings, 79(53.7%) had tape measures, 69(46.9%) had blood pressure (BP) apparatus, 98(66.7%) had stethoscopes, 16(10.9%) had watch or clock, and 102(69.4%) had examination couch. Whereas all of the providers reported that they had mid-upper arm circumference (MUAC) tape, which is used to assess malnutrition. Regarding job aids and tools, 145(98.6%) had iCCM register for 2-59 months old children, and 138(93.9%) had chart booklets.

Overall, 115 HEWs (71.4%) out of 147 had access to amoxicillin on the date of assessment. Oral rehydration solution medicines were available to 137 (93.2%) providers and zinc to 125(85%) providers. Both ORS and zinc medicines were available to 117(79.6%) providers (S1 Table).

Providers knowledge

Out of 274 providers who responded to the interview (S2 Table), less than half of them had good knowledge of pneumonia signs and symptoms (35.4%), diarrhea signs and symptoms (20.8%), pneumonia management approaches (24.5%), and diarrhea management approaches (24.1%). Overall, slightly over a quarter (27%) of the providers were knowledgeable about the signs and symptoms, and management approaches of both illnesses. Mean pneumonia and diarrhea signs and symptoms, and the management knowledge score of the HEWs was 48.31% (95% CI: 45.7, 50.9) with a standard deviation of 21.5.

Know-do gap in child healthcare

There was a significant difference between the knowledge of the HEWs and their actual practices in the assessment of pneumonia and diarrhea ($p<0.05$ for individual and overall know-do gap proportions as in table 2). Slightly greater than one-third (35.7%) of HEWs had good knowledge of pneumonia signs and symptoms and only one-fifth of them (19.8%) had actually assessed those

For peer review only - http://bmjopen.bmj.com/site/about/guidelines.xhtml

signs and symptoms. Though three-fourths of providers know cough or fast breathing as the sign and symptoms of pneumonia, only 47.3% of them have actually asked about cough or difficulty breathing. Likewise, 34.9% of providers have described chest-indrawing as a symptom of pneumonia while only 28.6% have assessed chest-indrawing in children.

Six out of ten (61.5%) of the providers have good knowledge of diarrhea signs and symptoms, however only four out of ten (40.2%) were able to actually assess those signs and symptoms in children who presented with diarrhea complaints.

Table 2. Providers know-do gap in the assessment of pneumonia and diarrhea in four regions of Ethiopia, December 2018 to February 2019

Assessment	Knowledge (n, %)	Practice (n, %)	Gap (%)	Z test p value
Pneumonia				
Cough or fast breathing	114(76.0)	71(47.3)	28.7	p<0.0001
Chest-indrawing[a]	44(34.9)	36(28.6)	6.3	0.20
Stridor[a]	35(27.8)	19(15.1)	12.7	0.006
Pneumonia index[a]	45(35.7)	25(19.8)	15.9	0.005
Diarrhea				
Presence of diarrhea	126(84.0)	62(41.3)	42.7	p<0.0001
Blood in the stool[b]	39(32.0)	52(42.6)	-10.6	0.17
Child restlessness or irritability[b]	39(32.0)	46(37.7)	-5.7	0.26
Pinch the skin of the abdomen[b]	56(45.9)	33(27.0)	18.9	0.001
Diarrhea index[b]	75(61.5)	49(40.2)	21.3	0.001
Overall index[c]	41(38.0)	23(21.3)	16.7	0.002

[a]*Out of the health extension workers that have consulted children who were labeled to have a cough or difficulty breathing (n=126)*

[b] *Out of the health extension workers that have consulted children who were labeled to have diarrhea (n=122)*

[c]*Out of the health extension workers that have consulted children who were labeled to have both cough and diarrhea (n=108)*

For peer review only - http://bmjopen.bmj.com/site/about/guidelines.xhtml

For peer review only

Similarly, a significant difference was observed between the knowledge and actual practice of providers regarding the treatment of childhood pneumonia and diarrhea ($p<0.05$). Most of the providers (96.2%) have reported that they knew treatment recommendations for pneumonia and diarrhea, but only a little more than a quarter of them (27.7%) have actually practiced the recommendations for children who were classified as having either pneumonia or diarrhea. On the contrary, a large proportion of providers practiced counseling about medications for children who were provided treatments for pneumonia and diarrhea though only a few of them know it as a component of management (Table 3).

Table 3. Providers know-do gap in the management of pneumonia and diarrhea in four regions of Ethiopia, December 2018 to February 2019

Treatment and counseling	Knowledge (n, %)	Practice (n, %)	Gap (%)	Z test p value
Pneumonia				
Prescribe antibiotics[a]	68(88.3)	12(15.6)	72.7	p<0.0001
Counsel on medications[b]	2(16.7)	10(83.3)	(66.6)	0.001
Diarrhea				
Prescribe ORS and zinc[c]	108(93.9)	59(51.3)	42.6	p<0.0001
Counsel on medications[d]	18(30.5)	30(50.8)	(20.3)	0.03
Treatment for pneumonia and diarrhea[e]	126(96.2)	36(27.7)	68.5	p<0.0001

[a]*Out of the health extension workers who have consulted children classified as having pneumonia (N=77)*
[b]*Out of the health extension worker who has provided treatment to pneumonia (N=12)*
[c]*Out of the health extension workers who have consulted children classified as having diarrhea (N=115)*
[d]*Out of the health extension workers who have provided treatment to diarrhea (N=59)*
[e] *Out of the health extension workers who have consulted children who were classified as having either pneumonia or diarrhea (N=130)*

Drivers of knowledge and actual practice of providers

Drivers of providers' knowledge

The majority of the providers who have service experience greater than 5 years (74.3%) as HEWs and who received iCCM training (83.8%) had good knowledge of pneumonia and diarrhea signs and symptoms and management approaches. Moreover, six out of ten of the providers who have

For peer review only - http://bmjopen.bmj.com/site/about/guidelines.xhtml

received supervision with components of discussing diagnosis and treatment of childhood illnesses and those who participated in the performance review clinical mentoring meetings had good knowledge.

In the bivariate analysis, providers' age, service experience, level of education, iCCM training, supportive supervision, and participation on performance review clinical mentoring meetings were identified as candidate variables for multiple variable logistic regression at p-value<0.2. In the multiple variable logistic regression, only the participation in the performance review clinical mentoring meetings showed statistically significant associations with the knowledge of the providers. Providers' knowledge of pneumonia and diarrhea management was nearly two times more likely among providers who were participated in the performance review clinical mentoring meetings that was held 6 months preceding the survey period (AOR=2.00, 95% CI: 1.09,3.67) (Table 4).

Table 4. Correlates of providers' knowledge of pneumonia and diarrhea management in four regions of Ethiopia, December 2018 to February 2019

Variables	Knowledge		AOR (95% CI)
	Good(n,%)	Poor(n,%)	
Observations	74	200	
Age in years			
≤30	43(58.1)	144(72.0)	Ref
>30	31(41.9)	56(28.0)	1.41(0.75-2.64)
Service experience as HEWs in years			
≤5	19(25.7)	87(43.5)	Ref
>5	55(74.3)	113(56.5)	1.78(0.81,3.92)
Level of HEWs			
Level-3 (Certificate)	34(45.9)	120(60.0)	Ref
Level-4 (Diploma)	40(54.1)	80(40.0)	1.33(0.74,2.39)
iCCM training			
No	12(16.2)	46(23.0)	Ref
Yes	62(83.8)	154(77.0)	0.71(0.29,1.71)
Supervision with discussion on diagnosis and treatment of pneumonia and diarrhea			
No	29(39.2)	103(51.5)	Ref

For peer review only - http://bmjopen.bmj.com/site/about/guidelines.xhtml

Yes	45(60.8)	97(48.5)	1.12(0.60,2.11)
Supervision with a component of observing client consultations			
No	38(51.3)	131(65.5)	Ref
Yes	36(48.7)	69(34.5)	1.36(0.73,2.53)
Being participated in PRCMM			
No	29(39.2)	119(59.5)	Ref
Yes	45(60.8)	81(40.5)	2.00(1.09,3.67)**

*** P<0.05 is considered statistically significant in the multivariate analysis*

Abbreviations: CI, confidence interval; iCCM, integrated community case management; PRCMM, performance review clinical mentoring meeting; HEW, health extension worker; AOR, adjusted odds ratio; Ref, reference category

Drivers of providers' actual practice

The majority of HEWs aged less than 30 years (77.8%) and those who have access to medicines for childhood conditions (83.3%) had better childhood pneumonia and diarrhea management practice. Moreover, greater than half of the HEWs who received supervision with a component of discussing the management of childhood conditions, and less than half of the providers who participated in the performance review clinical mentoring meetings had good actual management practice.

In the bivariate analysis, providers' age, service experience, level of education, iCCM training, supportive supervision, participation on performance review clinical mentoring meetings, knowledge of childhood pneumonia and diarrhea management, and availability of medicines were identified as candidate variables for multiple variable logistic regression at p-value<0.2. The multiple variable logistic regression analysis revealed that pneumonia and diarrhea medicines availability was associated with the actual management practices of providers. The rest of the variables didn't show associations. Correct management practice was four times more likely in providers who have access to medicines for the treatment of pneumonia and diarrhea compared to those who didn't have access (AOR=4.04, 95% CI: 1.48,11.06) (Table 5).

For peer review only - http://bmjopen.bmj.com/site/about/guidelines.xhtml

Table 5. Correlates of the providers' actual management practice of pneumonia and diarrhea in four regions of Ethiopia, December 2018 to February 2019

Variables	Practice		AOR (95% CI)
	Good(n,%)	Poor(n,%)	
Observations	36	94	
Age in years			
≤30	28(77.8)	70(74.5)	Ref
>30	8(22.2)	24(25.5)	0.61(0.22-1.75)
Service experience as HEWs in years			
≤5	14(38.9)	39(41.5)	Ref
>5	22(61.1)	55(58.5)	1.54(0.59-4.02)
Level of HEWs			
Level-3 (Certificate)	20(55.6)	59(62.8)	Ref
Level-4 (Diploma)	16(44.4)	35(37.2)	1.52(0.62-3.68)
iCCM training			
No	30(83.3)	75(79.8)	Ref
Yes	6(16.7)	19(20.2)	1.45(0.39-5.30)
Supervision with discussion on diagnosis and treatment of pneumonia and diarrhea			
No	15(41.7)	42(44.7)	Ref
Yes	21(58.6)	52(55.3)	1.13(0.44-2.87)
Supervision with a component of observing client consultations			
No	23(63.9)	58(61.7)	Ref
Yes	13(36.1)	36(38.3)	0.91(0.37-2.19)
Being participated in PRCMM			
No	19(52.8)	46(48.9)	Ref
Yes	17(47.2)	48(51.1)	0.60(0.24-1.50)
Knowledge index			
Poor knowledge	25(69.4)	67(71.3)	Ref
Good knowledge	11(30.6)	27(28.7)	1.04(0.41-2.64)
Amoxicillin, ORS and zinc availability[a]			
No	6(16.7)	38(40.4)	Ref
Yes	30(83.3)	53(59.6)	4.04(1.48-11.06)*

*** $P<0.05$ is considered statistically significant in the multivariate analysis*
Abbreviations: CI, confidence interval; iCCM, integrated community case management; PRCMM, performance review clinical mentoring meeting; HEW, health extension worker; AOR, adjusted odds ratio; Ref, reference category
[a]Out of providers who have assessed for medications availability in the health posts(n=127). Providers who had access to all the three medicines were regarded as 'yes' and otherwise 'no'.

Discussion

The study revealed that a low proportion of the providers had knowledge of the signs and symptoms and management of pneumonia and diarrhea. Similarly, a low proportion of the

For peer review only

providers executed the recommended actions for the assessment and management of childhood pneumonia and dehydration. There was a significant difference between the knowledge and actual practice of the providers. This finding is consistent with a result among other health professionals in Ethiopia (14) and frontline health workers in India (13,16).

Slightly greater than one-third (35.7%) of the HEWs had good knowledge of pneumonia signs and symptoms and only one-fifth of them (19.8%) had actually assessed consulting children for those signs and symptoms. The widest gap was observed between knowledge and actual practice of providers related to cough or difficulty breathing (76% vs. 47.3%) and stridor (27.8% vs. 15.1%). Besides, the incongruity between the knowledge and actual practices of the providers; the level of concordance with the clinical guidelines have remained low among the frontline providers. This has a huge impact on the clinical care outcomes related to childhood pneumonia. Poor adherence to clinical guidelines by providers in the assessment of sick child was also reported in earlier studies from low-and-middle-income countries (34,35) and in Ethiopia(27–29).

Though six out of ten (61.5%) of the providers had good knowledge of signs and symptoms of diarrhea, only four out of ten (40.2%) actually assessed those signs and symptoms in children who presented with diarrhea complaints. A wide and significant gap was observed between the knowledge and actual practice of the providers related to overall diarrhea assessment (84% vs. 41.3%) and pinch skin of the abdomen (45.9% vs. 27%). On the contrary, a large proportion of providers have practiced assessing the presence of blood in the stool (42.6% vs. 32%), and child restlessness or irritability (37.7% vs. 32%) without reporting on knowledge of the symptoms at the outset. Though the clinical guidelines recommend assessment of all the signs and symptoms of dehydration, the providers didn't actually practice them (32). This could lead to incorrect diagnosis

For peer review only - http://bmjopen.bmj.com/site/about/guidelines.xhtml

of sick children with possible dehydration and results in missed opportunity to provide life-saving treatment for those in need of it.

The know-do gap analysis also revealed that there was a significant difference between the knowledge and actual practice of providers. Most of the providers (96.2%) have reported that they know recommended treatment options for pneumonia and diarrhea, but only a quarter of them (27.7%) have actually provided treatment for children who were classified as having either pneumonia or diarrhea. Earlier studies from Ethiopia and Uganda also revealed that community health workers had poor knowledge and practice of childhood illnesses management (36–38). Contrarily, a large proportion of providers practiced counseling about medications for children who were provided treatment for pneumonia and diarrhea though only a few of them knew it as a component of sick child care.

Many factors counter play the lack of knowledge and good practice related to the assessment and management of sick children. The quality of clinical care provided to sick children is a function of the providers' knowledge, the effort exerted by the provider to apply the knowledge, and the readiness of health facilities to provide quality care in terms of medicines and supplies. In the current study, the providers' knowledge and actual practice of assessment and management of childhood pneumonia and diarrhea were associated with participation in performance review clinical mentoring meeting and the availability of medicines for pneumonia and dehydration treatment at the health posts. A previous study also revealed that clinical coaching is positively associated with good clinical practice among community health workers (39). The odds of good knowledge of pneumonia and diarrhea assessment and management was two times more likely in the providers who participated in the performance review clinical mentoring meeting. While, the odds of better correct practice were four times more likely in providers who had medicines for the

For peer review only - http://bmjopen.bmj.com/site/about/guidelines.xhtml

treatment of childhood pneumonia and diarrhea (amoxicillin, ORS, and zinc). Other variables including the providers' characteristics (age, work or practice experience, level of training), receiving clinical training, and supportive supervision did not affect knowledge and actual practice of the HEWs. Though most of the providers were trained in iCCM, they scored low in the knowledge and practice for assessment and treatment of childhood pneumonia implying that in-serving trainings are necessary but not sufficient drivers of correct practice(40). In another study, variables including the providers' level of education, work or practice experience, the presence of supervision, and guidelines were associated with the community health workers' knowledge of childhood illness management (37,38).

The findings of the present study have policy and practice implications. Clinical training interventions that lacked adequate practical skill component doesn't improve the competency of providers. The lack of refresher training is also a possible cause for poor performance due to the decay in knowledge. High-quality in-service training combined with mentoring, supervisions, medicines, and material support are effective to improve the quality of care(40–42). Training had high coverage in the study area, but significant number of providers are short of iCCM medicines, supplies, and technical support. Supervisions and mentoring focused on the management of childhood pneumonia and diarrhea were limited. The supervisors discussed the management of pneumonia and diarrhea in half of their visits (51.8%) and in greater than one-third (38.3%) they observed client consultations by the provider. Less than half (46%) of providers were clinically mentored according to the standard guideline, and two-thirds (65.7%) of them had access to medicines for pneumonia and diarrhea. In the study area, in-service training and supervision were not associated with knowledge, and actual practices of providers signaling a look at other approaches that improves their quality.

For peer review only - http://bmjopen.bmj.com/site/about/guidelines.xhtml

The study was conducted in the community health posts within the primary healthcare level, the lowest care level in the health system of Ethiopia. At this level the providers are supposed to diagnose and treat moderate cases, and refer severe cases to the health centers. This is the first study that examined the know-do gap in childhood pneumonia and diarrhea care among HEWs in Ethiopia. The know-do gap analysis was based on observations of actual management practices of the providers by experienced and trained health professionals, and interviews on the knowledge assessment components. Though our findings are limited by a small sample size, the study has covered the four most populous regions of the country and is representative of the different settings in rural Ethiopia. The small sample size might have caused underestimated standard errors. On average the providers were observed while consulting five sick children, and this might ensure the heterogeneity of cases for both conditions. However, the clinical practices and actions of providers related to severe illnesses was not examined in the present research. The other limitation might be the Hawthorne effect i.e., a change in professional behavior of the HEWs when being observed (43). This might partly mask the actual practice of providers. But, the findings related to actual management practice in both conditions shown that the providers have performed less and in normal management situation this might be even worse.

Conclusions

The study revealed a huge know-do gap in the assessment and management of childhood pneumonia and diarrhea among the HEWs in Ethiopia. The finding affirm that knowledge-based training is necessary but not sufficient to influence the clinical performance (actual practice) of providers. The know-do gap of pneumonia and diarrhea management was determined by the presence of clinical mentoring and performance reviews and the availability of medicines. Therefore, an effort should be made to close the gap between theoretical knowledge and practice.

For peer review only - http://bmjopen.bmj.com/site/about/guidelines.xhtml

The HEWs should be supported with mentoring, performance reviews, and the supply of medicines and commodities.

Acknowledgments

The authors acknowledge the administrative hierarchies and the research participants in the study area for their cooperation throughout the research process, and the time they devoted to the study in providing required information.

Authors' contributions

All authors made a significant contribution to the work reported, whether, that is, in the conception, study design, execution, acquisition of data, analysis, and interpretation, or in all these areas. DWD wrote the first draft of the manuscript, and MAW and MW revised the manuscript. All authors gave final approval of the version to be published, have agreed on the journal to which the article has been submitted, and agreed to be accountable for all aspects of the work. All authors read and approved the final manuscript.

Funding

This project was funded by Bill & Melinda Gates Foundation(INV-009691).

Disclaimer

The funder has no role in the study design, collection, management, analysis or interpretation of data.

For peer review only

Data availability

The datasets used and/or analyzed for the current study are not openly available because data are part of the ongoing research project and available from the corresponding author upon reasonable request (E-mail: dawit.daka86520@gmail.com; or dawit.daka@ju.edu.et).

Competing interests

All authors declare that they have no competing interests.

Patient consent for publication

Not required

References

1. United Nations Children's Fund (UNICEF). Pneumonia and diarrhoea: Tackling the deadliest diseases for the world's poorest children. United Nations Children's Fund. 2012. 1–86 p. Available from: http://www.unicef.org/eapro/Pneumonia _and_ Diarrhoea_ Report_2012.pdf

2. United Nations Children's Fund (UNICEF). One is too many: Ending child deaths from pneumonia and diarrhoea. 2016. 25 p. Available from: https://data.unicef.org/wp-content/uploads/2016/11/UNICEF-Pneumonia-Diarrhoea-report2016-web-version_final.pdf

3. IVAC at Johns Hopkins Bloomberg School of Public Health. Pneumonia and Diarrhea Progress Report 2018. 2018. Available from: http://link.springer.com/10.1023/A:1020102612073%0Ahttp://apps.who.int/gb/ebwha/pdf_files/WHA66/A66_R10-en.pdf%0Ahttp://www.who.int/diabetes/country-profiles/nga_en.pdf?ua=1

For peer review only - http://bmjopen.bmj.com/site/about/guidelines.xhtml

4. International Vaccine Access Center (IVAC). Pneumonia and Diarrhea Progress Report 2020. John Hopkins Bloomberg School of Public Health. 2020. Available from: https://resourcecentre.savethechildren.net/pdf/ivac_pdpr_2020.pdf/

5. Nanyonjo A, Ssekitooleko J, Counihan H, Makumbi F, Tomson G, Källander K. Impact of an integrated community case management programme on uptake of appropriate diarrhoea and pneumonia treatments in Uganda: A propensity score matching and equity analysis study. Int J Equity Health. 2015;14:74. Available from: http://dx.doi.org/10.1186/s12939-015-0202-y

6. Winskill P, Mousa A, Oresanya O, Counihan H, Okell LC, Walker PG. Does integrated community case management (iCCM) target health inequities and treatment delays? Evidence from an analysis of Demographic and Health Surveys data from 21 countries in the period 2010 to 2018. J Glob Health. 2021;11:040113.

7. Young M, Wolfheim C, Marsh DR, Hammamy D. World health organization/United Nations children's fund joint statement on integrated community case management: An equity-focused strategy to improve access to essential treatment services for children. Am J Trop Med Hyg. 2012;87(SUPPL.5):6–10.

8. World Health Organization. Integrated Management of Childhood Illness (IMCI): Chart Booklet. Geneva, Switzerland: World Health Organization. 2014.

9. Kallander K, Burgess DH, Qazi SA. Early identification and treatment of pneumonia: A call to action. Lancet Glob Heal. 2016;4(1):e12–3.

10. HEFDC Group. Integrated community case management (iCCM) in sub-Saharan Africa: Successes and challenges with access, speed and quality. Thematic Review Report. 2018.

For peer review only - http://bmjopen.bmj.com/site/about/guidelines.xhtml

11. Bagonza J, Kibira SPS, Rutebemberwa E. Performance of community health workers managing malaria , pneumonia and diarrhoea under the community case management programme in central Uganda : a cross sectional study. Malar J. 2014;13:367.

12. Chinbuah MA, Abbey M, Kager PA, Gyapong M, Nonvignon J, Ashitey P, et al. Assessment of the adherence of community health workers to dosing and referral guidelines for the management of fever in children under 5 years: a study in Dangme West District, Ghana. Int Health. 2013;5:148–56.

13. Ray Saraswati L, Baker M, Mishra A, Bhandari P, Rai A, Mishra P, et al. 'Know-Can' gap: gap between knowledge and skills related to childhood diarrhoea and pneumonia among frontline workers in rural Uttar Pradesh, India. Trop Med Int Heal. 2020;25(4):454–66.

14. Gage AD, Kruk ME, Girma T, Lemango ET. The know-do gap in sick child care in Ethiopia. PLoS One. 2018;13(12):e0208898.

15. Tielsch JM. The Know, Do, and Quality Gaps in International Maternal and Child Health Care Interventions. JAMA Pediatr. 2015 Apr 1;169(4):313–4. Available from: https://doi.org/10.1001/jamapediatrics.2014.3741

16. Mohanan M, Vera-Hernández M, Das V et al. The Know-Do Gap in Quality of Health Care for Childhood Diarrhea and Pneumonia in Rural India. Jama Pediatr. 2015;169(4):349–57.

17. Mungai IG, Baghel SS, Soni S, Vagela S, Sharma M, Diwan V, et al. Identifying the know-do gap in evidence-based neonatal care practices among informal health care providers—a cross-sectional study from Ujjain, India. BMC Health Serv Res.

For peer review only - http://bmjopen.bmj.com/site/about/guidelines.xhtml

2020;20:966.

18. Wanduru P, Tetui M, Tuhebwe D, Ediau M, Okuga M, Nalwadda C, et al. The performance of community health workers in the management of multiple childhood infectious diseases in Lira, northern Uganda – a mixed methods cross-sectional study. Glob Health Action. 2016 Dec 1;9:33194. Available from: https://doi.org/10.3402/gha.v9.33194

19. Rabbani F, Perveen S, Aftab W, Zahidie A, Sangrasi K, Qazi SA. Health workers' perspectives, knowledge and skills regarding community case management of childhood diarrhoea and pneumonia: A qualitative inquiry for an implementation research project "nigraan" in District Badin, Sindh, Pakistan. BMC Health Serv Res. 2016;16:462. Available from: http://dx.doi.org/10.1186/s12913-016-1699-5

20. Izudi J, Anyigu S, Ndungutse D. Adherence to Integrated Management of Childhood Illnesses Guideline in Treating South Sudanese Children with Cough or Difficulty in Breathing. Menahem S, editor. Int J Pediatr. 2017;2017:5173416. Available from: https://doi.org/10.1155/2017/5173416

21. Miller NP, Amouzou A, Tafesse M, Hazel E, Legesse H, Degefie T, et al. Integrated Community Case Management of Childhood Illness in Ethiopia : Implementation Strength and Quality of Care. Am J Trop Med Hyg. 2014;91(2):424–34.

22. Sinyangwe C, Graham K, Nicholas S, King R, Mukupa S, Källander K, et al. Assessing the quality of care for pneumonia in integrated community case management: A cross-sectional mixed methods study. PLoS One. 2016;11(3):e0152204.

23. Baynes C, Mboya D, Likasi S, Maganga D, Pemba S, Baraka J, et al. Quality of sick

For peer review only - http://bmjopen.bmj.com/site/about/guidelines.xhtml

child-care delivered by community health workers in Tanzania. Int J Heal Policy Manag. 2018;7(12):1097–109. Available from: https://doi.org/10.15171/ijhpm.2018.63

24. Berhanu A, Alemayehu M, Daka K, Binu W, Suleiman M. Utilization of Integrated Community Case Management of Childhood Illnesses at Health Posts in Southern Ethiopia. Pediatr Heal Med Ther. 2020;Volume 11:459–67.

25. Assefa Y, Gelaw YA, Hill PS, Taye BW, Van Damme W. Community health extension program of Ethiopia, 2003–2018: successes and challenges toward universal coverage for primary healthcare services. Global Health. 2019;15(1):24. Available from: https://doi.org/10.1186/s12992-019-0470-1

26. Legesse H, Degefie T, Hiluf M, Sime K, Tesfaye C, Abebe H, et al. National scale-up of integrated community case management in rural Ethiopia: Implementation and early lessons learned. Ethiop Med J. 2014;52(December):15–26.

27. Wolde D, Daka, Wordofa MA, Woldie M, Persson Å, Berhanu D. Quality of clinical assessment and management of sick children by Health Extension Workers in four regions of Ethiopia : A cross-sectional survey. PLoS One. 2020;15(9):e0239361.

28. Beyene H, Hailu D, Tadele H, Persson LÅ, Berhanu D. Insufficient referral practices of sick children in Ethiopia shown in a cross-sectional survey. Acta Paediatr Int J Paediatr. 2020;109(9):1867–74.

29. Getachew T, Mekonnen S, Yitayal M, Persson LÅ, Berhanu D. Health Extension Workers' diagnostic accuracy for common childhood illnesses in four regions of Ethiopia: a cross-sectional study. Acta Paediatr Int J Paediatr. 2019;108(11):2100–6. Available from: https://onlinelibrary.wiley.com/doi/epdf/10.1111/apa.14888

For peer review only - http://bmjopen.bmj.com/site/about/guidelines.xhtml

30. Berhanu D, Okwaraji YB, Belayneh AB, Lemango ET, Agonafer N, Birhanu BG et al. Protocol for the evaluation of a complex intervention aiming at increased utilization of primary child health services in Ethiopia: A before and after study in intervention and comparison areas. BMC Health Serv Res. 2010;20:339.

31. World Health Organization. Health facility survey : tool to evaluate the quality of care delivered to sick children attending outpatient facilities. Geneva PP - Geneva: World Health Organization; Available from: https://apps.who.int/iris/handle/10665/42643

32. Ministry of Health of Ethiopia. The Integrated Community Case Management (iCCM) Chart Booklet Ethiopia. 2014. Available from: http://lifesavingcommodities.org/wp-content/uploads/2014/08/iCCM-English-Chartbooklet-August-2013.pdf

33. Tesema AG, Peiris D, Abimbola S, Ajisegiri WS, Narasimhan P, Mulugeta A, et al. Community health extension workers' training and supervision in Ethiopia: Exploring impact and implementation challenges for non-communicable disease service delivery. PLOS Glob Public Heal. 2022 Nov 9;2(11):e0001160. Available from: https://doi.org/10.1371/journal.pgph.0001160

34. Gilroy KE, Callaghan-koru JA, Cardemil C V, Nsona H, Amouzou A, Mtimuni A, et al. Quality of sick child care delivered by Health Surveillance Assistants in Malawi. Health Policy Plan. 2013;28:573–85.

35. Kruk RME, Gaga AD, Mbaruku GM, Leslie HH. Content of Care in 15,000 Sick Child Consultations in Nine Lower-Income Countries. Heal Serv Res. 2018;12842. Available from: https://doi.org/10.1111/1475-6773.12842).

36. Bagonza J, Kibira SPS, Rutebemberwa E. Performance of community health workers

For peer review only

For peer review only - http://bmjopen.bmj.com/site/about/guidelines.xhtml

managing malaria, pneumonia and diarrhoea under the community case management programme in central Uganda: A cross sectional study. Malar J. 2014;13(1):1–10.

37. Befekadu A, Yitayal M. Knowledge and practice of health extension workers on drug provision for childhood illness in west Gojjam, Amhara, Northwest Ethiopia. BMC Public Health [Internet]. 2020;20:496. Available from: https://doi.org/10.1186/s12889-020-08602-y

38. Wanduru P, Tetui M, Tuhebwe D, Ediau M, Okuga M, Nalwadda C, et al. The performance of community health workers in the management of multiple childhood infectious diseases in Lira, northern Uganda - A mixed methods cross-sectional study. Glob Health Action. 2016;9(1).

39. Shiroya-Wandabwa M, Kabue M, Kasungami D, Wambua J, Otieno D, Waka C, et al. Coaching community health volunteers in integrated community case management improves the care of sick children under-5: Experience from Bondo, Kenya. Int J Integr Care. 2018;18(4):1–11.

40. Leslie HH, Gage A, Nsona H, Hirschhorn LR, Kruk ME. Training And Supervision Did Not Meaningfully Improve Quality Of Care For Pregnant Women Or Sick Children In Sub-Saharan Africa. Health Aff. 2016 Sep 1;35(9):1716–24. Available from: https://doi.org/10.1377/hlthaff.2016.0261

41. Bluestone J, Troncoso E, Fitzgerald L, Nagbe L, Tetteh G, Hellar A, et al. Three Pivots for Improving Health Care Provider Performance. Glob Heal Sci Pract. 2022 Feb 28;10(1):e2100625. Available from: http://www.ghspjournal.org/content/10/1/e2100625.abstract

For peer review only

For peer review only - http://bmjopen.bmj.com/site/about/guidelines.xhtml

42. Desta BF, Beshir IA, Tefera BB, Argaw MD, Demeke HZ, Kibret MA. Does frequency of supportive supervisory visits influence health service delivery?—Dose and response study. PLoS One. 2020 Jun 16;15(6):e0234819. Available from: https://doi.org/10.1371/journal.pone.0234819

43. Oswald D. Handling the Hawthorne effect: The challenges surrounding a participant observer. Rev Soc Stud. 2014 Oct 1;1:53–74.

For peer review only

S1 Table. Medical equipment, job aids, and medicine availability at the health posts of providers in four regions of Ethiopia, December 2018 to February 2019 (N=147)

Medical equipment, job aids, and Medicines	n(%)
Medical equipment	
Thermometer	121(82.3)
Infant scale	115(78.2)
Weighing sling	116(78.9)
Tape measures	79(53.7)
Blood pressure apparatus	69(46.9)
Stethoscopes	98(66.7)
Watch or clock (timer)	16(10.9)
Examination couch	102(69.4)
Mid-upper-arm circumference tape	147(100.0)
Basic equipment availability index[a]	44(29.9)
Job aids	
iCCM register (2-59 months old children)	145(98.6)
Stock/bin cards to track medicines	97(66.0)
Medicines request and resupply forms	95(64.6)
Chart booklets	138(93.9)
Medicines	
Amoxicillin	115(78.2)
Oral rehydration solution	137(93.2)
Zinc	125(85.0)
Oral rehydration solution and zinc	117(79.6)
Paracetamol	94(63.9)
Amoxicillin, oral rehydration solution and zinc	96(65.3)

[a] *Basic equipment availability index: Availability of all of the following for the same provider: thermometer, infant scale, weighing sling, stethoscope, and BP apparatus*

For peer review only

S2 Table. Providers knowledge of childhood pneumonia and diarrhea management in four regions of Ethiopia, December 2018 to February 2019 (N=274)

Knowledge of-	n	%(95% CI)
Pneumonia signs and symptoms		
Good knowledge	97	35.4(29.9-41.2)
Poor knowledge	177	64.6(58.8-70.1)
Pneumonia management approaches		
Good knowledge	67	24.5(19.6-29.8)
Poor knowledge	207	75.5(70.2-80.4)
Pneumonia signs and symptoms and management approaches		
Good knowledge	108	39.4(33.7-45.3)
Poor knowledge	166	60.6(54.7-66.3)
Diarrhea signs and symptoms		
Good knowledge	57	20.8(16.3-25.9)
Poor knowledge	217	79.2(74.1-83.7)
Diarrhea management approaches		
Good knowledge	66	24.1(19.3-29.4)
Poor knowledge	208	75.9(70.6-80.7)
Diarrhea signs and symptoms and management approaches		
Good knowledge	78	28.5(23.4-34.0)
Poor knowledge	196	71.5(66.0-76.6)
Both illnesses sign and symptoms and management approaches		
Good knowledge	74	27.0(22.0-32.5)
Poor knowledge	200	73.0(67.5-78.0)

For peer review only

STROBE Statement—Checklist of items that should be included in reports of ***cross-sectional studies***

	Item No	Recommendation	Page No
**Title and abstract**	1	(*a*) Indicate the study's design with a commonly used term in the title or the abstract	2
		(*b*) Provide in the abstract an informative and balanced summary of what was done and what was found	2&3
**Introduction**			
Background/rationale	2	Explain the scientific background and rationale for the investigation being reported	3-5
Objectives	3	State specific objectives, including any prespecified hypotheses	5
**Methods**			
Study design	4	Present key elements of study design early in the paper	5
Setting	5	Describe the setting, locations, and relevant dates, including periods of recruitment, exposure, follow-up, and data collection	5-6
Participants	6	(*a*) Give the eligibility criteria, and the sources and methods of selection of participants	6
Variables	7	Clearly define all outcomes, exposures, predictors, potential confounders, and effect modifiers. Give diagnostic criteria, if applicable	7-8
Data sources/ measurement	8*	For each variable of interest, give sources of data and details of methods of assessment (measurement). Describe comparability of assessment methods if there is more than one group	6-7
Bias	9	Describe any efforts to address potential sources of bias	7
Study size	10	Explain how the study size was arrived at	6
Quantitative variables	11	Explain how quantitative variables were handled in the analyses. If applicable, describe which groupings were chosen and why	9
Statistical methods	12	(*a*) Describe all statistical methods, including those used to control for confounding	9
		(*b*) Describe any methods used to examine subgroups and interactions	
		(*c*) Explain how missing data were addressed	
		(*d*) If applicable, describe analytical methods taking account of sampling strategy	
		(*e*) Describe any sensitivity analyses	
**Results**			
Participants	13*	(a) Report numbers of individuals at each stage of study—eg numbers potentially eligible, examined for eligibility, confirmed eligible, included in the study, completing follow-up, and analysed	10
		(b) Give reasons for non-participation at each stage	
		(c) Consider use of a flow diagram	
Descriptive data	14*	(a) Give characteristics of study participants (eg demographic, clinical, social) and information on exposures and potential confounders	10-11
		(b) Indicate number of participants with missing data for each variable of interest	
Outcome data	15*	Report numbers of outcome events or summary measures	13-16
Main results	16	(*a*) Give unadjusted estimates and, if applicable, confounder-adjusted estimates and their precision (eg, 95% confidence interval). Make clear which confounders were adjusted for and why they were included	16-18

For peer review only

		(*b*) Report category boundaries when continuous variables were categorized	
		(*c*) If relevant, consider translating estimates of relative risk into absolute risk for a meaningful time period	
Other analyses	17	Report other analyses done—eg analyses of subgroups and interactions, and sensitivity analyses	
**Discussion**			
Key results	18	Summarise key results with reference to study objectives	18
Limitations	19	Discuss limitations of the study, taking into account sources of potential bias or imprecision. Discuss both direction and magnitude of any potential bias	20-21
Interpretation	20	Give a cautious overall interpretation of results considering objectives, limitations, multiplicity of analyses, results from similar studies, and other relevant evidence	18-21
Generalisability	21	Discuss the generalisability (external validity) of the study results	18-21
**Other information**			
Funding	22	Give the source of funding and the role of the funders for the present study and, if applicable, for the original study on which the present article is based	22

*Give information separately for exposed and unexposed groups.

Note: An Explanation and Elaboration article discusses each checklist item and gives methodological background and published examples of transparent reporting. The STROBE checklist is best used in conjunction with this article (freely available on the Web sites of PLoS Medicine at http://www.plosmedicine.org/, Annals of Internal Medicine at http://www.annals.org/, and Epidemiology at http://www.epidem.com/). Information on the STROBE Initiative is available at www.strobe-statement.org.

For peer review only
